# Genome-wide detection of human variants that disrupt intronic branchpoints

**DOI:** 10.1073/pnas.2211194119

**Published:** 2022-10-28

**Authors:** Peng Zhang, Quentin Philippot, Weicheng Ren, Wei-Te Lei, Juan Li, Peter D. Stenson, Pere Soler Palacín, Roger Colobran, Bertrand Boisson, Shen-Ying Zhang, Anne Puel, Qiang Pan-Hammarström, Qian Zhang, David N. Cooper, Laurent Abel, Jean-Laurent Casanova

**Affiliations:** ^a^St. Giles Laboratory of Human Genetics of Infectious Diseases, Rockefeller Branch, The Rockefeller University, New York, NY 10065;; ^b^Laboratory of Human Genetics of Infectious Diseases, Necker Branch, INSERM UMR1163, 75015 Paris, France;; ^c^Paris Cité University, Imagine Institute, 75015 Paris, France;; ^d^Department of Biosciences and Nutrition, Karolinska Institutet, 14183 Huddinge, Sweden;; ^e^Institute of Medical Genetics, School of Medicine, Cardiff University, Cardiff CF14 4XN, United Kingdom;; ^f^Infection in Immunocompromised Pediatric Patients Research Group, Vall d’Hebron Research Institute, Vall d’Hebron Barcelona Hospital Campus, Vall d’Hebron University Hospital, 08035 Barcelona, Spain;; ^g^Pediatric Infectious Diseases and Immunodeficiencies Unit, Vall d’Hebron University Hospital, Vall d’Hebron Research Institute, Vall d’Hebron Barcelona Hospital Campus, Autonomous University of Barcelona, 08035 Barcelona, Spain;; ^h^Jeffrey Modell Diagnostic and Research Center for Primary Immunodeficiencies, 08035 Barcelona, Spain;; ^i^Diagnostic Immunology Group, Vall d’Hebron Research Institute, Vall d’Hebron Barcelona Hospital Campus, Vall d’Hebron University Hospital, 08035 Barcelona, Spain;; ^j^Immunology Division, Genetics Department, Vall d’Hebron University Hospital, Vall d’Hebron Barcelona Hospital Campus, Autonomous University of Barcelona, 08035 Barcelona, Spain;; ^k^HHMI, New York, NY 10065

**Keywords:** branchpoint, splicing, intronic variant, disease genetics, software

## Abstract

Pre-messenger RNA splicing is initiated with the recognition of a single-nucleotide intronic branchpoint (BP) within a BP motif by spliceosome elements. Forty-eight rare variants in 43 human genes have been reported to alter splicing and cause disease by disrupting BP. However, until now, no computational approach was available to efficiently detect such variants in massively parallel sequencing data. We established a comprehensive human genome-wide BP database by integrating existing BP data and generating new BP data from RNA sequencing of lariat debranching enzyme DBR1-mutated patients and from machine-learning predictions. We characterized multiple features of BP in major and minor introns and found that BP and BP-2 (two nucleotides upstream of BP) positions exhibit a lower rate of variation in human populations and higher evolutionary conservation than the intronic background, while being comparable to the exonic background. We developed BPHunter as a genome-wide computational approach to systematically and efficiently detect intronic variants that may disrupt BP recognition. BPHunter retrospectively identified 40 of the 48 known pathogenic BP variants, in which we summarized a strategy for prioritizing BP variant candidates. The remaining eight variants all create AG-dinucleotides between the BP and acceptor site, which is the likely reason for missplicing. We demonstrated the practical utility of BPHunter prospectively by using it to identify a novel germline heterozygous BP variant of *STAT2* in a patient with critical COVID-19 pneumonia and a novel somatic intronic 59-nucleotide deletion of *ITPKB* in a lymphoma patient, both of which were validated experimentally. BPHunter is publicly available from https://hgidsoft.rockefeller.edu/BPHunter and https://github.com/casanova-lab/BPHunter.

## Significance

The search for candidate variants underlying human disease in massive parallel sequencing data typically focuses on coding regions and essential splice sites, mostly ignoring noncoding variants. The RNA spliceosome recognizes intronic branchpoint (BP) motifs at the beginning of splicing and operates mostly within introns to define the exon–intron boundaries; however, BP variants have been paid little attention. We established a comprehensive genome-wide database and knowledgebase of BP and developed BPHunter for systematic and informative genome-wide detection of intronic variants that may disrupt BP and splicing, together with an effective strategy for prioritizing BP variant candidates. BPHunter not only constitutes an important resource for understanding BP, but should also drive discovery of BP variants in human genetic diseases and traits.

**Fig. 1.**
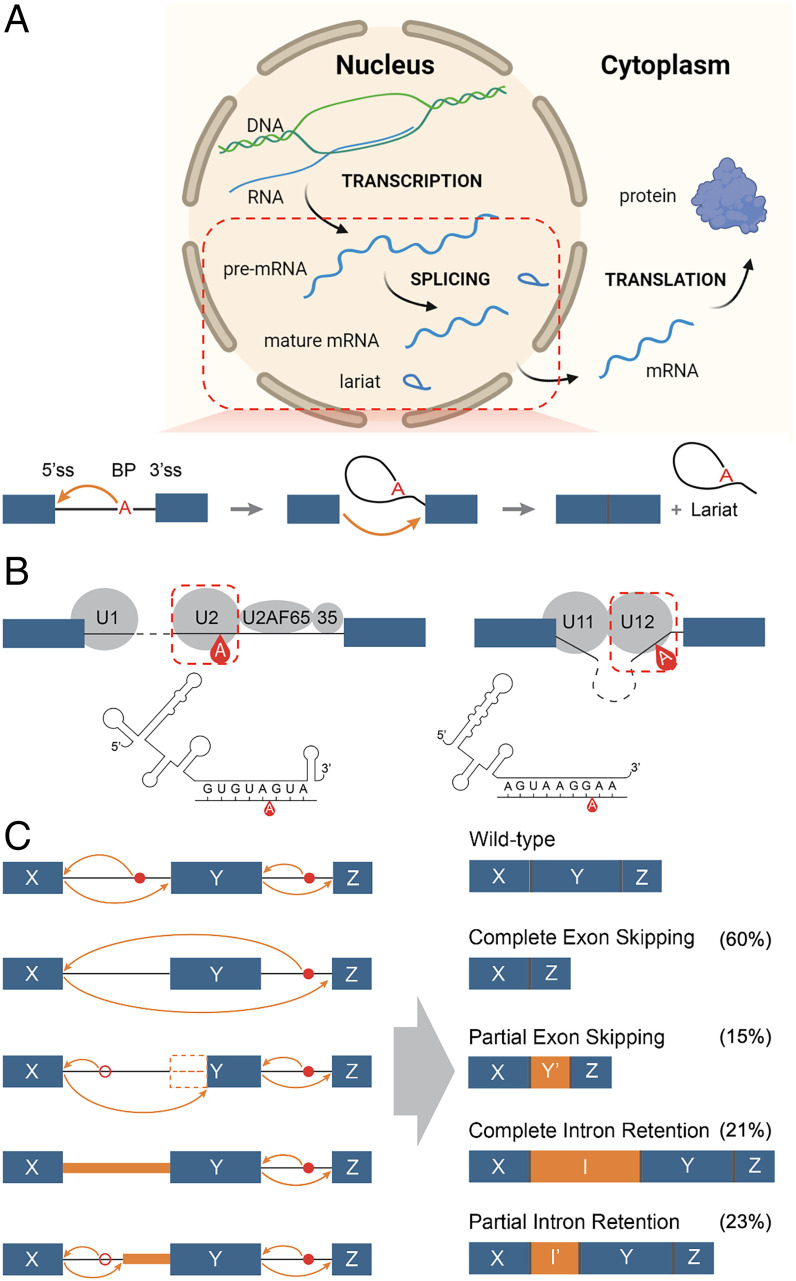
Schematics of a splicing event, the two types of spliceosome, and typical consequences of a BP variant. (*A*) Schematic of the biological processes of transcription, splicing, and translation from DNA to pre-mRNA to mature mRNA and protein. In pre-mRNA splicing, the BP is first recognized, two exons are then joined, and the intervening intron is finally released as a circular lariat. (*B*) Schematic of the major (U2-type, on the left) and minor (U12-type, on the right) spliceosomes, with an illustration of the interaction between pre-mRNA sequence and U2/U12 snRNA. (*C*) Schematic of the potential molecular consequences of a BP variant, including complete/partial exon skipping and complete/partial intron retention. The percentage in parentheses refer to the observed fraction of each category from the published pathogenic BP variants (note that one variant could result in more than one missplicing consequence).

Pre-mRNA (messenger RNA) splicing is a necessary step for protein-coding gene expression in eukaryotic cells. The biochemical process is initiated by the recognition of the branchpoint (BP) in an intron, followed by the identification and ligation of the 5′ splice site (5′ss = donor site) and 3′ splice site (3′ss = acceptor site) to join two exons and the removal of the intervening intron as a circular lariat, to yield a mature mRNA of coding sequence for protein translation (Fig. 1*A*). Splicing takes place in the nucleus and is regulated by an array of *cis*-acting elements (the RNA sequences with their splicing codes) and *trans*-acting elements (proteins and small nuclear RNAs (snRNAs) that bind to the *cis*-acting elements), which together constitute a garden around the splice sites promoting the formation of the complex and dynamic spliceosome (1–3). When splicing is initiated, the BP motif is recognized by a spliceosomal snRNA, with the cooperation of splicing factors (4, 5). BP recognition is achieved differently between the two types of spliceosome (Fig. 1*B*): the major (U2-type, in >99% of human introns) and the minor (U12-type). The main difference between these spliceosomes is the use of different snRNAs. The major spliceosome recruits U1, U2, U4, U5, and U6 snRNAs, in which the U1 and U2 snRNAs recognize 5′ss and BP independently (1). In the interaction between BP and U2 snRNA, the BP nucleotide bulges out to bind into a pocket formed by SF3B1 and PHF5A (6), and its flanking sequences base-pair with U2 snRNA (5, 7), while U2 snRNA is stabilized by SF3B6 (6). The minor spliceosome recruits U11, U12, U4atac, U5, and U6atac snRNAs, in which the U11 and U12 snRNAs first form a di-snRNA complex before recognizing the 5′ss and BP simultaneously (7, 8).

BP constitutes a single-nucleotide *cis*-acting element within the intron. It is typically an adenine and generally located [−20, −40] (from −20 to −40) nucleotides (nt) away from the 3′ss. The BP motif is thought to follow the consensus sequence YTNAY (4) (A denotes the BP site, Y = C/T, *n* = any nucleotide). There are also distal BP deeper inside introns: recursive splicing within the intron for multistep intron removal (9), stem-loop RNA structures bring distal BP closer to 3′ss (10), stochastic splice site selection leads to kinetic variation in intron removal (11) (*SI Appendix*, Fig. S1). So far, considerable efforts have been made to identify BP positions, including a lariat sequencing method to study BP in *Saccharomyces cerevisiae* (12), a spliceosome profiling method to sequence RNA copurifying with spliceosomal complex in *Schizosaccharomyces pombe* (13), and analyzing large-scale experimental data in humans [RNA sequencing (RNA-seq) (5, 14–17) and iCLIP (18)]. To locate BP positions from RNA-seq data, the sequencing reads from the circular lariats that traverse the junctions between 5′ss and BP are identified by aligning to the intronic sequences of 5′ss and 3′ss, respectively. Since lariats are transient and only exist at low abundance, their detection is technically difficult, hence large-scale data are required for identifying BP positions. Spliceosome iCLIP experimentation identifies cross-links of spliceosomal factors on pre-mRNA sequences at nucleotide-level resolution to pinpoint BP positions (18). Some machine-learning models have also been developed to predict BP positions based on one or two experimentally identified BP datasets (19–21). However, these individual BP studies have yet to be integrated into a comprehensive database and knowledgebase of human BP.

The functional role of splicing makes it a critical process for the correct assembly of gene products, while its complexity renders it vulnerable to deleterious variants that can perturb any part of the splicing machinery. A variant may affect a *cis*- or *trans*-element, leading to altered splicing with alternative 5′ss/3′ss, exon skipping, or intron retention, any of which could result in the synthesis of a defective gene product (22, 23). As previously reported (24–26), at least 10% of disease-causing variants are associated with missplicing [of which ∼65% are in essential exonic/intronic splice sites, ∼25% are in the remainder of the intron, and ∼10% are in the remainder of the exon (25)]; this may, however, be a serious underestimate (24) owing to our rather limited ability to detect variants that impair different aspects of the spliceosome. Currently, the search for candidate pathogenic variants in massively parallel sequence (MPS) data generally focuses on nonsynonymous variants located within coding sequences or variants in essential splice sites, while mostly ignoring synonymous coding exonic variants and noncoding intronic variants. Data from RNA-seq or other studies of mRNA occasionally lead to the detection of intronic or exonic variants that disrupt splicing (27). However, the spliceosome operates mostly within intronic regions to define the exon–intron boundaries and hence the coding sequences. It follows that introns probably harbor a substantially larger number of pathogenic variants than has so far been appreciated (28). The BP, involved in the initiation of splicing through its interactions with spliceosome elements, constitutes a key vulnerability of splicing by virtue of its potential variants.

Employing the Human Gene Mutation Database (HGMD) Professional version (25) in concert with an in-depth literature review, we identified 48 BP variants reported in 43 genes underlying 33 human diseases whose pathogenicity has been supported by accompanying experimental evidence. These BP variants resulted in complete/partial exon skipping or intron retention, or a combination of these consequences (Fig. 1*C*). The misspliced mRNAs may be rapidly degraded by nonsense-mediated decay (NMD), resulting in loss of expression, and the translated truncated proteins could exhibit loss of function, both being potentially disease-causing. However, since the first report of a disease-causing BP variant in the *L1CAM* gene in a family with X-linked hydrocephalus in 1992 (29), the discovery rate of pathogenic BP variants appears to have been rather low (only 48 variants discovered in the last 30 y). This is probably because 1) genome-wide knowledge of BP has not been systematically integrated and characterized, 2) genome-wide detection software has not been available for the efficient identification of BP variants from MPS data, and 3) in-depth understanding of all reported pathogenic BP variants has not been analyzed, which could potentiate the strategic prioritization of candidate variants that could functionally disrupt BP. We have therefore aimed to fill these gaps both for biological and medical reasons.

## Results

### Collection of Experimentally Identified BP Data.

We first collated five datasets of experimentally identified BP (eBP), based on RNA-seq and iCLIP data: eBP_Mercer (59,359 BP from total RNA-seq) (5), eBP_Taggart (27,795 BP from total RNA-seq) (15), eBP_Pineda (138,313 BP from total RNA-seq) (16), eBP_Talhouarne (240 BP from cytoplasmic RNA-seq) (17), and eBP_Briese (43,630 BP from iCLIP) (18) (Table 1). In addition to the published BP datasets, we revisited our RNA-seq data derived from *DBR1*-mutated patients with brainstem viral infection (30). *DBR1* encodes the only known lariat debranching enzyme, and the patients with *DBR1* variants, who were characterized by a residual DBR1 activity of 3 to 10%, have been reported to exhibit an elevated cellular level of lariats (30). We designed a computational program to detect BP positions from RNA-seq reads (*Methods*), which identified 8,682 BP sites (eBP_BPHunter) from 15 RNA-seq samples from the fibroblasts of three *DBR1*-mutated patients (*SI Appendix*, Fig. S2*A* and Table S1). In these six eBP datasets, the most abundant BP nucleotides were eBP_Mercer (79% A), eBP_Taggart (68% A), eBP_Pineda (75% A), eBP_Talhouarne (84% C), eBP_Briese (100% A), and eBP_BPHunter (46% A) (Table 1). This showed that adenine-BP were the most frequent in lariats from total RNA-seq, whereas cytosine-BP were the most frequent in the stable lariats in cytoplasm albeit in very low amounts (17). *DBR1*-mutated patients had 22% stable cytosine-BP. The iCLIP-identified BP were invariably adenine. In total, we obtained 210,986 unique eBP.

**Table 1.** A comprehensive collection of eBP and cBP, including the additional BP data generated in this study (eBP_BPHunter and cBP_BPHunter), followed by consensus-guided positional adjustment

BP dataset	No. of raw BP	A, %	C, %	G, %	T, %	No. of adj. BP	A, %	C, %	G, %	T, %	Δ no. of BP
eBP_Mercer	59,359	78.5	8.5	4.7	8.4	54,531	84.9	8.0	3.5	3.6	−4,828
eBP_Taggart	27,795	67.8	15.3	5.5	11.4	27,635	73.7	13.1	4.1	9.1	−160
eBP_Pineda	138,313	75.0	7.6	9.5	7.9	133,652	77.0	7.3	8.9	6.9	−4,661
eBP_Talhouarne	240	4.6	84.2	4.6	6.7	240	8.3	81.7	3.3	6.7	0
eBP_Briese	43,630	100.0	0.0	0.0	0.0	43,630	100.0	0.0	0.0	0.0	0
eBP_BPHunter	8,682	45.9	22.4	18.2	13.5	7,990	56.6	21.8	15.7	5.8	−692
eBP	210,986	73.4	9.1	8.5	9.1	198,256	76.9	8.6	7.7	6.9	−12,730
cBP_BPP	232,828	98.8	1.1	0.0	0.2	232,820	98.8	1.1	0.0	0.2	−8
cBP_Branchpointer	352,322	98.8	0.5	0.0	0.7	325,304	99.5	0.6	0.0	0.0	−27,018
cBP_LaBranchoR	203,914	98.3	1.6	0.0	0.0	203,907	98.3	1.6	0.0	0.0	−7
cBP_BPHunter	20,718	100.0	0.0	0.0	0.0	20,718	100.0	0.0	0.0	0.0	0
cBP	479,366	97.9	1.5	0.0	0.6	451,215	98.3	1.6	0.0	0.1	−28,151
Total BP						546,559	90.40	4.25	2.78	2.57	
mBP						102,912	99.15	0.83	0.01	0.01	

### Collection of Computationally Predicted BP Data.

We also collated three datasets of computationally predicted BP (cBP): cBP_BPP (232,828 BP) (19), cBP_Branchpointer (352,322 BP) (20), and cBP_LaBranchoR (203,914 BP) (21) (Table 1). These studies trained machine-learning models based on one/two eBP datasets and performed predictions in the region [−21, −34], [−18, −44] and [−1, −70] nt of 3′ss respectively. These cBP datasets supplemented the BP positions that were not detected from experimental data and hence increased the BP coverage across the human genome. These computational methods overlapped the predictions in [−21, −34] nt of 3′ss, with less attention being paid to in the region closer to 3′ss, which is generally considered to comprise polypyrimidine tracts (PPT). However, the use of a hard distance cutoff for PPT or the use of an algorithm to estimate PPT to skip BP prediction could miss potentially genuine BP sites. For this reason, we postulated that there could be additional BP candidates in the region closer to 3′ss. Therefore, we developed a high-precision machine-learning model: a set of three algorithms (gradient boost machine, random forest, and logistic regression) that are tuned for high precision and integrated with majority voting for the final prediction (*Methods*). These three models were independently trained on the 198,256 consensus-guided position-adjusted eBP positions (discussed in the next section; *SI Appendix*, Fig. S2 *B* and *C*) versus 1 million random intronic/exonic positions and then optimized by tuning parameters and thresholds through cross-validation (*SI Appendix*, Fig. S2*D*). We used the model to predict BP in [−3, −40] nt of 3′ss, and it yielded 20,718 BP sites (cBP_BPHunter). In these four cBP datasets, the most abundant BP nucleotides were cBP_BPP (99% A), cBP_Branchpointer (99% A), cBP_LaBranchoR (98% A), and cBP_BPHunter (100% A) (Table 1), showing that the prediction models favored adenine-BP. In total, we obtained 479,366 unique cBP. Please note that cBP does not denote cytosine-BP. When we mention the nucleotides of BP, we fully spell out the nucleotide (e.g., adenine-BP, cytosine-BP).

### Consensus-Guided Positional Adjustment of BP and Integration of BP Datasets.

**Fig. 2.**
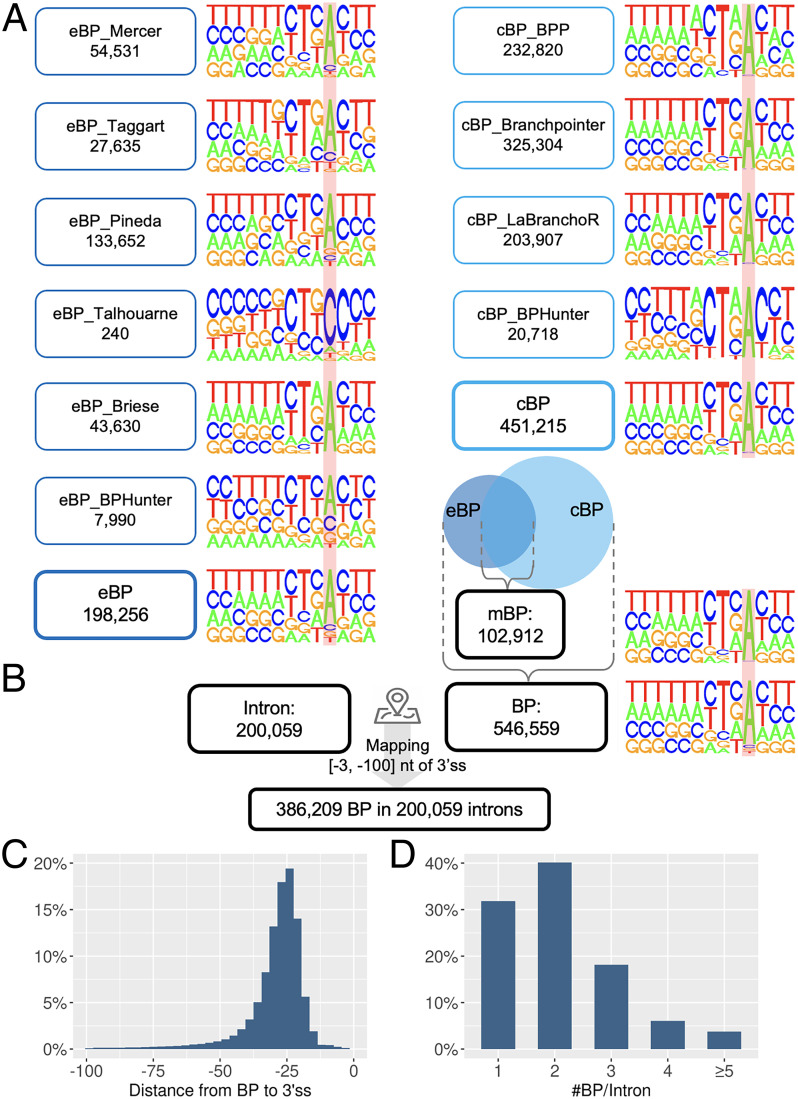
Integration of all BP datasets and mapping of BP onto introns. (*A*) The nucleotide composition displays the motif [−9, +3] of BP, where the locations of BP are marked with a red background. (*B*) BP were mapped to 3′-proximal intron. (*C*) Distance from the mapped BP to their corresponding 3′ss. (*D*) The number of BP mapped to each 3′-proximal intron.

We tested the established BP consensus sequence YTNAY (4) in eBP but found only 23.5% eBP matched this pattern precisely. Considering the noncanonical 2′-to-5′ linkage between 5′ss and BP, and the possibility of mutations being introduced by reverse transcriptase when traversing the 5′ss-BP junction in RNA-seq (5, 15), we anticipated that a number of eBP may have been mislocated in the raw dataset (*SI Appendix*, Fig. S2*B*), which could also have led to some mislocated cBP predictions. We therefore screened a window of [−2, +2] nt from each BP position for consensus sequence matching and adjusted the raw BP position to its closest neighbor that perfectly matched YTNAY (*SI Appendix*, Fig. S2*C*). As a result, about 6% of raw BP positions were adjusted (*SI Appendix*, Table S2), and multiple neighboring raw BP positions were merged into a single adjusted position, yielding 198,256 eBP (Δ = −12,730) and 451,215 cBP (Δ = −28,151) after adjustment (Table 1). By integrating eBP and cBP, we assembled a comprehensive collection of 546,559 BP in the human genome, with 102,912 mutually shared BP (mBP) (Fig. 2*A*, Table 1*, SI Appendix*, Fig. S3 and Table S3, and Dataset S1). This BP database contained 90.4% adenine-BP, 4.25% cytosine-BP, 2.78% guanine-BP, and 2.57% thymine-BP. We further decomposed adenine-BP by adopting increasingly relaxed consensus sequences: YTNAY (35.8%), YTNA (53.65%), TNA (74.32%), and YNA (77.24%) (*SI Appendix*, Fig. S4). We then examined the region [−50, +20] nt of BP for motif enrichment and reported 14 enriched motifs (*P* < 0.05, occurrence >5%) in the 50-nt upstream region of BP, which could represent *cis*-acting elements with a role in facilitating splicing (*SI Appendix*, Fig. S5).

### Mapping BP to Introns.

We identified 200,059 unique introns from 41,975 transcripts of 17,372 protein-coding genes and classified introns into 199,393 (99.7%) major and 666 (0.3%) minor types (*SI Appendix*, Fig. S6 and *Methods*). Because the major and minor spliceosomes regulate splicing differently (7) (Fig. 1*B*), it is important to distinguish intron types when studying BP and splicing. Major introns were dominated by GT-AG (98.9%) as the 5′ss–3′ss terminal dinucleotides, whereas 71% and 24% of minor introns were GT-AG and AT-AC, respectively. We mapped 546,559 BP onto 200,059 introns, retained the BP within [−3, −100] nt of 3′ss (3′-proximal introns), and finally obtained 386,209 BP associated with 200,059 introns (Fig. 2*B* and Datasets S2 and S3). We excluded the distal BP in this study, as more complicated splicing mechanisms may occur (*SI Appendix*, Fig. S1). Among the intron-associated BP, 57% of them were located within a 10-nt window [−21, −30] nt of 3′ss, and 88% were located within a 25-nt window [−15, −40] nt of 3′ss (Fig. 2*C*); 32%, 41%, and 18% of 3′-proximal introns had one, two, and three BP mapped, respectively, while 9% of 3′-proximal introns harbored more than three BP (Fig. 2*D*). With 68% of 3′-proximal introns containing multiple BP, we found that these BP were largely located in proximity. By taking the first BP in each intron as the reference (i.e., the one closest to the 3′ss), 31% and 59% of nonfirst BP were found to be located within 5 nt and 10 nt upstream of the first BP (*SI Appendix*, Fig. S7). With multiple BP, the first BP could be the most important, as one study showed that BP closer to 3′ss were much more likely to influence splicing efficiency (31). The BP usage is also expected to be context-specific in alternatively spliced transcripts, but we do not have the requisite data to differentiate the usage. Thus, in this study, we considered the BP–intron associations in a general setting.

### Nucleotide Composition, BP-3′ss Distance, and BP-U2/U12 snRNA Binding Energy in Major and Minor Introns.

**Fig. 3.**
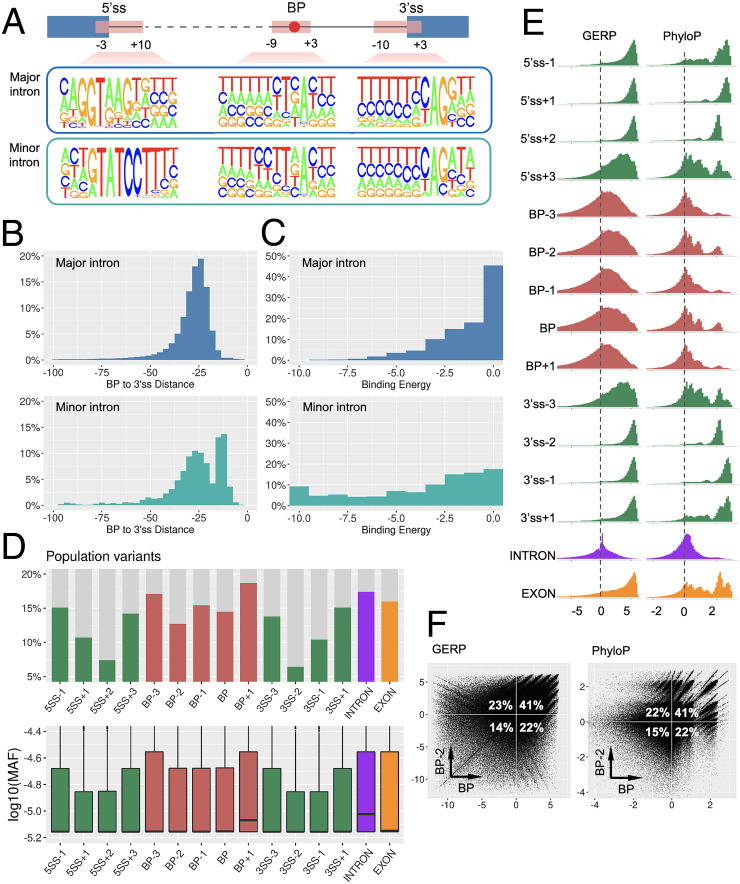
Characterization of BP. (*A*) Nucleotide composition of BP, 5′ss, and 3′ss motifs in major and minor introns. (*B*) The distance from BP to 3′ss in major and minor introns. (*C*) The binding energy between BP motif and U2/U12 snRNA in major and minor introns (the lower the energy, the higher the binding affinity). (*D*) The proportion of each genomic position harboring population variants (*Upper*), and the MAF distribution of population variants (*Lower*). (*E*) The distribution of the conservation scores GERP (*Left*) and PhyloP (*Right*) in each genomic position. (*F*) The cross-compared conservation scores between BP and BP−2 positions.

In major introns, BP motifs exhibited high conservation of A (92%) at pos:0 and T (72%) at pos:−2 and high pyrimidine content (C/T) at pos:−2 (84%), pos: −3 (69%), and pos:+1 (66%). In minor introns, BP showed high conservation of A (95%) at pos:0 and T (78%) at pos: −2 and high pyrimidine content at pos:[−2, −5] and pos:+1 with frequencies 89%, 75%, 67%, 66%, and 67%, respectively (Fig. 3*A* and *SI Appendix*, Table S4). We found 2% and 67% of BP in major introns were distributed in regions [−3, −15] and [−16, −30] nt of 3′ss, whereas 27% and 43% of BP in minor introns were found within these two regions (Fig. 3*B* and *SI Appendix*, Table S5). BP in minor introns were generally located closer to 3′ss than BP in major introns, which is consistent with the major spliceosome requiring the binding of U2AF65 with PPT that needs a larger spacing, which is absent in the minor spliceosome (7). We further estimated the binding energy between BP and snRNA and found that BP in major introns displayed weaker binding with U2-snRNA than that between BP and U12-snRNA in minor introns (Fig. 3*C* and *SI Appendix*, Table S5). This may imply that the BP–U2 interaction in the major spliceosome can be complemented by other spliceosomal elements (e.g., SF3B1, SF3B6, PHF5A, SFA2, SFA3) (6), and hence a weaker wobble BP–U2 base pairing could be acceptable. Since the minor spliceosome forms a U11–U12 di-snRNA complex to bind to 5′ss and BP simultaneously, it requires more perfect BP–U12 base pairing, which is also reflected in more conserved 5′ss and BP motifs in minor introns (Fig. 3*A*).

### Population Variants in BP.

We obtained human population variants from the gnomAD database r3.1.1 (32), with which to compare the occurrence of population variants in the BP region, essential/flanking splice sites, and intronic/exonic background. We focused on the [−3, +1] positions of 386,209 BP, took a 4-nt window for each splice site ([−1, +3] of 5′ss and [−3, +1] of 3′ss) from 200,059 introns and randomly sampled 1 million positions from the intron and exon backgrounds. In this way, we generated 15 sets of genomic locations for comparison with their population variants (Fig. 3*D* and *SI Appendix*, Table S6). We found that 17.4% of intronic and 16% of exonic background positions harbored population variants; 13.9 to 15.1% of the flanking splice sites had population variants, whereas the essential splice sites exhibited a much lower proportion (6.4 to 10.7%). Among the BP regions, 14.5% of BP positions, 15.4% of BP−1 positions, and 12.7% of BP−2 positions harbored population variants, which were lower than those for the intron and exon backgrounds and similar to the flanking positions of splice sites. The other two positions in the BP region (BP−3 and BP+1) had 17.1% and 18.7% of population variants respectively, which were close to or even higher than the intronic background. The minor allele frequencies (MAF) of these population variants at each position also suggested a similar pattern (Fig. 3*D*). An extended window around splice sites ([−2, +5] of 5′ss and [−5, +2] of 3′ss) is shown in *SI Appendix*, Fig. S8. These findings suggested that the BP and BP−2 positions exhibit a lower rate and a lower frequency of population variations than the intronic background and are similar to the exonic background and flanking positions of essential splice sites.

### Cross-Species Conservation of BP.

We evaluated genome-wide conservation scores, GERP (33) and PhyloP (34), in the abovementioned 15 sets of genomic locations. A positive score denotes that the position is likely to be evolutionarily conserved, whereas a negative score indicates that the position is probably evolving neutrally (Fig. 3*E* and *SI Appendix*, Table S7). Both scores were found to be comparable for each position studied. The essential splice sites and their exonic flanking regions displayed an absolute positive distribution of conservation scores, whereas their intronic flanking sites exhibited a reduced level of conservation. Intronic positions were mostly around zero, whereas exonic positions were mostly positive. The BP region displayed a larger proportion of positive scores (evolutionarily conserved) than negative scores (evolving neutrally), with two peaks of high PhyloP scores at BP and BP−2 positions. We cross-compared the scores between BP and BP−2 (Fig. 3*F*) and found that ∼23% of negatively scored BP positions had positive scores for their corresponding BP−2 positions (second quadrant), while ∼22% of negatively scored BP−2 positions had positive scores for their corresponding BP positions (fourth quadrant). Thus, if we consider at least one positive score for BP or BP−2 position, 86% of BP regions were evolutionarily conserved. Taken together, these findings suggested that the BP region is more highly conserved than the intronic background but less conserved than the exonic background and splice sites. It may also hint at a possible collaborative role between BP and BP−2 to ensure a certain level of conservation. An extended window around splice sites is shown in *SI Appendix*, Fig. S9. A recent study of the bovine genome showed that bovine BP are under evolutionary constraint (35). It is worth noting that splicing evolves rapidly between species (36), with one study showing significant splicing differences between primates, including human and chimpanzee (37). This suggests that the conservation scores of splicing regulatory elements may not be invariably correlated with their functional importance. In other words, BP with low/negative conservation scores could be functionally important.

### Comparative Characterization of BP.

We made seven comparisons of BP in the major introns, at intron, transcript, gene, and chromosome levels: 1) 353,031 adenine-BP vs. 14,476 cytosine-BP vs. 9,626 guanine-BP vs. 7,381 thymine-BP (*SI Appendix*, Fig. S10). We found that adenine-/thymine-BP had much lower rates of population variation than cytosine-/guanine-BP, and they had two spikes of high PhyloP conservation scores. 2) 94,581 mBP vs. 60,450 exclusively eBP vs. 229,483 exclusively cBP (*SI Appendix*, Fig. S11). mBP displayed the most conserved nucleotide composition, the lowest rate of population variation, and the largest proportion of positive conservation scores, followed by exclusively cBP and then exclusively eBP. 3) 180,766 first BP vs. 203,748 nonfirst BP (*SI Appendix*, Fig. S12) had no distinct feature noted. 4) 57,254 BP in 3′-proximal introns with single BP vs. 327,260 BP in 3′-proximal introns with multiple BP (*SI Appendix*, Fig. S13). BP in introns of single BP showed an absolute dominance (96%) of T in the BP−2 position, a lower rate of population variation, and a larger proportion of positive conservation scores. 5) 335,617 BP in introns without alternative splicing vs. 48,897 BP in introns with alternative splicing (*SI Appendix*, Fig. S14) had no notable difference. 6) 148,571 BP in genes encoding a single isoform vs. 235,943 BP in genes encoding multiple isoforms (*SI Appendix*, Fig. S15) had no notable difference. 7) 13,876 BP in genes located on the sex (X and Y) chromosomes vs. 370,638 BP in genes located on the autosomes (*SI Appendix*, Fig. S16). The sex chromosomes had a slightly higher proportion of 3′-proximal introns with single BP (36% vs. 31%) than the autosomes. We found that 8.7% of BP on sex chromosomes and 15% of BP on autosomes harbored population variants, revealing a lower rate of population variation of BP on sex chromosomes than autosomes. This may be due to the three population copies of X chromosomes (two copies in females, but one copy in males) compared to four population copies of autosomes (two copies in both females and males) and some other factors (e.g., effective population size, demography) (38).

### Pathogenic BP Variants.

Variants in BP may lead to reduced, altered, or abolished binding to spliceosome elements during the initiation of splicing, leading to the disruption of splicing and giving rise to misspliced gene products with the consequent loss of expression/function (Fig. 1*C*). Through the HGMD database and primary literature, we identified 48 pathogenic BP variants (42 single-nucleotide variants [SNVs] and 6 microdeletions) reported in 43 human genes underlying 33 different disorders that had been experimentally confirmed (Table 2 and *SI Appendix*, Table S8), from the first report of a BP variant in the *L1CAM* gene causing X-linked hydrocephalus in 1992 (29) to a recent study of BP variants in hereditary cancer genes in 2021 (39). These disorders were attributed to different classes of disease: developmental, oncogenic, metabolic, neurological, immunological, and circulatory. These BP variants comprised 16 homozygotes, 23 heterozygotes, 6 hemizygotes, and 3 without zygosity information and were located within [−9, −40] nt of 3′ss. Taking the reported missplicing consequences together (one variant may have multiple missplicing consequences), we identified 29 (60%) complete and 7 (15%) partial exon skippings and 10 (21%) complete and 11 (23%) partial intron retentions, suggesting that complete exon skipping may be the predominant molecular consequence from the disruption of BP recognition. The discovery narrative of these pathogenic BP variants generally occurred in a similar context (*SI Appendix*, Box S1). Their discovery timeline showed that 11, 15, and 22 pathogenic BP variants were published in the 1990s, the 2000s, and since 2010, respectively. Thus, neither the advent of MPS technology (since 2010) nor the publication of large-scale human BP datasets (since 2015) has resulted in a major increase in the identification of pathogenic BP variants. In fact, paradoxically, the proportion of pathogenic BP variants among all pathogenic variants discovered since the 1990s has steadily decreased. The detection efficiency of pathogenic BP variants is rather low when compared with other types of variant. Therefore, there is an urgent need to bridge BP data with MPS data, thereby extending the utility of the MPS data, making more practical use of the BP data and contributing to pathogenetic discovery.

**Table 2.** The 48 reported pathogenic BP variants underlying human inherited disorders, with experimentally confirmed molecular consequences

Pathogenic BP variants					BPHunter detection	
Gene	Variant	Distance to 3′ss	Disease	Consequence	Rank	Hit position
*ABCC8*	g.17452526T > C	−20	Hyperinsulinemic hypoglycemia	Partial retention of intron-11 (73nt)	#1/2	0
*ALPL*	g.21896765_21896784del	−33	Hypophosphatasia	Complete skipping of exon-8 and exon-7/8	#1/1	−2|−1|0
*BBS1*	g.66287067A > T	−21	Retinitis pigmentosa	Complete skipping of exon-8 and exon-7/8, partial skipping of exon-8 (30 nt)	#1/1	0
*BTK*	g.100609705T > C	−23	Agammaglobulinemia	Complete skipping of exon-16	#1/1	0
*C21orf2*	g.45750232T > A	−23	Axial spondylometaphyseal dysplasia	Complete retention of intron-6	#2/3	0
*CAPN3*	g.42684808del	−29	Calpainopathy	Partial retention of intron-6 (389 nt)	#1/1	−2
*CD40LG*	g.135736500_135736507del	−32	X-linked hyper-IgM syndrome	Complete skipping of exon-3	#1/1	−2|−1|0
*CDT1*	g.88873665A > G	−24	Meier-Gorlin syndrome	Complete retention of intron-8, complete skipping of exon-9	#1/1	0
*COL4A5*	g.107849932A > G	−40	Alport syndrome	Complete skipping of exon-29, partial skipping of exon-29 (43 nt)	#1/1	0
*COL5A1*	g.137686903T > G	−25	Ehlers-Danlos syndrome type II	Partial skipping of exon-33 (45 nt)	#1/2	−2
*COL7A1*	g.48616971T > C	−23	Dystrophic epidermolysis bullosa	Complete retention of intron-58 and intron-58/59, complete skipping of exon-59	#1/1	0
*CPS1*	g.211452758A > G	−24	Hyperammonemia	Complete skipping of exon-7	#1/2	0
*DYSF*	g.71817308A > G	−33	Limb-girdle muscular dystrophy	Complete retention of intron-31	#2/2	0
*ENG*	g.130578354A > G	−22	Pulmonary arterial hypertension	Complete skipping of exon-13	#1/2	−2
*F8*	g.154130469T > C	−27	Hemophilia A	Complete skipping of exon-19	#1/1	0
*F9*	g.138619496A > G	−25	Hemophilia B	Partial retention of intron-2 (25 nt)	#1/1	0
*FAS*	g.90770494A > G	−16	Autoimmune lymphoproliferative syndrome	Complete skipping of exon-6	N.D.	
*FBN2*	g.127670562A > C	−26	Congenital contractural arachnodactyly	Complete skipping of exon-31	#1/1	−2
*FBN2*	g.127671284T > C	−15	Congenital contractural arachnodactyly	Complete skipping of exon-29	N.D.	
*FGD1*	g.54476769del	−35	Aarskog–Scott syndrome	Complete skipping of exon-13	#1/1	0
*HEXB*	g.74014605A > G	−17	Sandhoff disease	Partial retention of intron-10 (37nt)	N.D.	
*IKBKG*	g.153788599A > T	−23	Ectodermal dysplasia with primary immunodeficiencies	Complete skipping of exon-5 and exon-3/4/5/6, complete retention of intron-4	#1/1	0
*ITGB2*	g.46321660A > C	−12	Leukocyte adhesion deficiency	Partial skipping of exon-6 (149 nt)	N.D.	
*ITGB4*	g.73732344T > A	−25	Epidermolysis bullosa with pyloric atresia	Complete retention of intron-14 and intron-14/15	#1/1	−2
*ITGB4*	g.73748508T > A	−19	Epidermolysis bullosa with pyloric atresia	Complete retention of intron-31, partial skipping of exon-32 (38 nt)	#1/1	−2
*KCNH2*	g.150646165T > C	−28	Long QT syndrome	Partial retention of intron-9 (147 nt)	#1/1	0
*L1CAM*	g.153131293T > G	−19	X-linked hydrocephalus	Complete skipping of exon-19, partial retention of intron-18 (69 nt)	#2/3	0
*LCAT*	g.67976512A > G	−22	Fish-eye disease	Complete retention of intron-4	#1/2	−2
*LIPC*	g.58830518A > G	−14	Hypertriglyceridemia and cardiovascular disease	Complete retention of intron-1, partial retention of intron-1 (13 nt, 78 nt)	N.D.	
*LMX1B*	g.129377625_129377641del	−37	Nail patella syndrome	Complete skipping of exon-2	#1/1	−2|−1|0
*MLH1*	g.37090369T > G	−26	Inherited cancer	Complete skipping of exon-17	#2/3	−2
*MLH1*	g.37090371A > G	−24	Inherited cancer	Complete skipping of exon-17	#2/3	0
*MSH2*	g.47709894A > G	−24	Inherited cancer	Complete skipping of exon-16 and 3′ untranslated region, partial retention of intron-15 (85 nt, 141 nt)	#1/1	0
*MSH6*	g.48032731T > G	−26	Inherited cancer	Complete skipping of exon-5	#2/3	−2
*NPC1*	g.21137182T > C	−28	Niemann-Pick type C disease	Complete skipping of exon-7	#1/1	0
*NTRK1*	g.156843392T > A	−33	Congenital insensitivity to pain with anhidrosis	Partial retention of intron-7 (137nt)	#1/1	−2
*RB1*	g.49039315A > T	−26	Retinoblastoma	Complete skipping of exon-24	#1/2	−1
*SLC25A20*	g.48921567A > C	−10	Carnitineacylcarnitine translocase deficiency	Complete skipping of exon-3 and exon-3/4	N.D.	
*SLC5A2*	g.31499327_31499349del	−31	Familial renal glycosuria	Complete skipping of exon-8	#1/2	−2|−1|0
*TH*	g.2187017A > T	−24	Extrapyramidal movement disorder	Complete skipping of exon-12, partial retention of intron-11 (36 nt)	#3/3	−2
*TSC2*	g.2138031A > G	−18	Tuberous sclerosis	Complete retention of intron-38, partial skipping of exon-39 (74 nt)	#1/2	0
*UROS*	g.127477605A > C	−31	Congenital erythropoietic porphyria	Partial retention of intron-9 (81 nt, 246 nt, 358 nt, 523 nt)	#1/1	−2
*USH2A*	g.216040529T > C	−17	Usher syndrome	Partial skipping of exon-44 (39 nt)	N.D.	
*VMA21*	g.150572076A > C	−27	Autophagic vacuolar myopathy	Showed significant reduction of expression and activity	#1/1	0
*VMA21*	g.150572076A > T	−27	Autophagic vacuolar myopathy	Showed significant reduction of expression and activity	#1/1	0
*VWF*	g.6101204T > A	−20	von Willebrand disease	Complete skipping of exon-38	#1/2	0
*XPC*	g.14209889A > T	−9	Xeroderma pigmentosum	Complete skipping of exon-4	N.D.	
*XPC*	g.14209904T > C	−24	Xeroderma pigmentosum	Complete skipping of exon-4	#1/1	0

All references are available in *SI Appendix*, Table S8. Full information on these variants with BPHunter’s annotations are available in Dataset S4. (The genomic positions are on the hg19/GRCh37 human genome assembly. Hit position: BP positions disrupted by the variant. N.D.: not detected.)

### BPHunter: Detection of Intronic Variants That Disrupt the BP.

Variants that affect BP recognition may be individually rare, but their impact on splicing, gene expression, and protein function can be highly deleterious. Therefore, in searching for disease-causing variants, we should routinely consider BP variants as a discrete category of potentially damaging variants, especially when the search within coding regions and essential splice sites has turned out to be unproductive. We therefore developed BPHunter for the genome-wide detection of human intronic variants that disrupt the BP in MPS data, thereby enabling the identification of BP variant candidates that could potentially result in aberrant splicing at a biochemical level and hence disease at a physiological level. Given a VCF file of variants, BPHunter detects those variants that disrupt BP with informative outputs (Box 1). We developed the BPHunter as a one-line command that can be easily implemented into MPS analysis and also as a user-friendly webserver for users with less computational expertise. We believe that there may have been many negative findings involving BP variants that were tested but never reported. To improve our understanding of functional BP, their mutational consequences, their utilization of alternative BP, and their cell-type specificity, we ought to respect the potential value to be derived from these negative data. Therefore, we created a reporting platform in the BPHunter webserver for researchers to submit their tested BP variants that do not display aberrant splicing. These data will be periodically collated and analyzed, and the report will become available on our website and as a preprint, with all contributing researchers acknowledged as members of the BPHunter Effort Group. As we cannot validate the submitted negative BP variants centrally, we shall require all investigators to take responsibility for their submitted data. Therefore, BPHunter constitutes a computational method capable of detecting candidate BP-disrupting variants and a platform for collating the negative results from BP genetics studies that should not be wasted.

**Box 1: Input and Output of BPHunter**

BPHunter supports both hg19/GRCh37 and hg38/GRCh38 human reference genomes and provides an option to focus on all or canonical transcripts. Given a VCF file of variants with the first five columns as mandatory fields (CHROM, POS, ID, REF, ALT), BPHunter outputs a text file of the variants that disrupt [−2, 0] nt region of BP, with the following informative annotations:

• variant type (snv, x nt-deletion, x nt-insertion)
• gene symbol
• BP name (m/e/cBP_chrom_pos_strand_nucleotide)
• BP ranking in this 3′-proximal intron (#rank/total)
• BP position hit (−2, −1, 0)
• distance from BP to 3′ss
• level of consensus (1:YTNAY, 2:YTNA, 3:TNA, 4:YNA, 0:none)
• number of sources, and list of sources
• population variant MAF at BP and BP-2 positions (noted as “.” if no variant found)
• conservation scores GERP and PhyloP at BP and BP−2 positions
• intron type (major, minor), and intron length
• transcript ID and intron number (e.g., ENST123456789_IVS10)
• BPHunter score (*SI Appendix*, Fig. S20)

In addition, the BPHunter webserver provides a negative BP reporting platform, which requires the submitters to provide variant information, cell type, assay type, and experimental evidence following the template (Dataset S6).

### Validation of BPHunter on the Reported Pathogenic BP Variants.

**Fig. 4.**
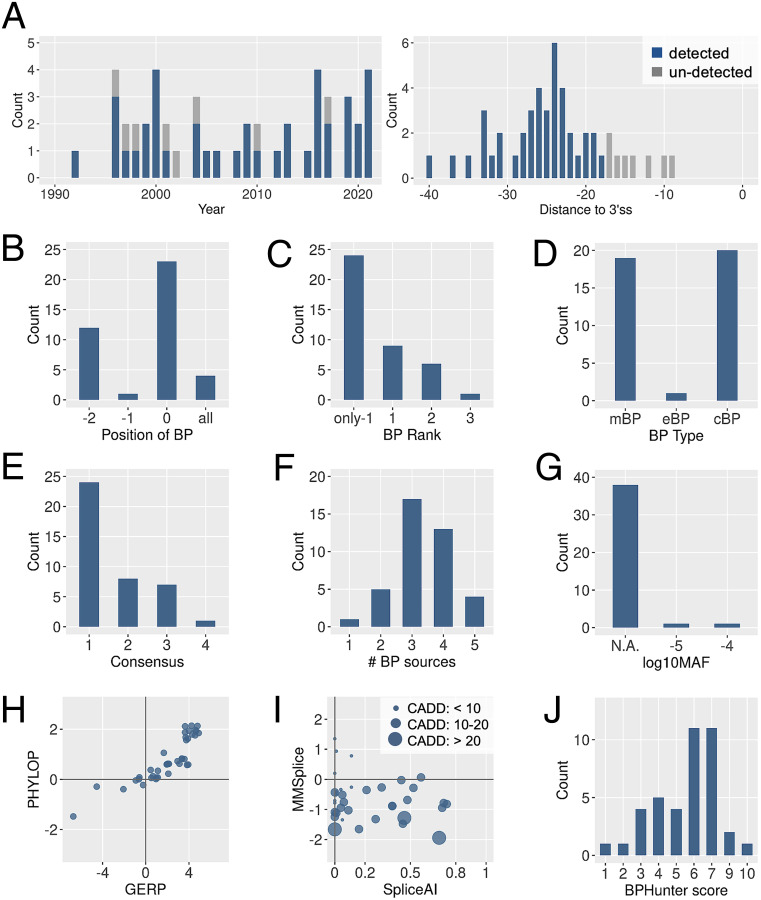
Detection of pathogenic BP variants by BPHunter. (*A*) Timeline of the 48 published pathogenic BP variants (*Left*) and the distance to their 3′ss (*Right*). (*B*) Disrupted positions of BP. (*C*) Ranking of the disrupted BP in its 3′-proximal intron. (*D*) Type of disrupted BP. (*E*) Level of consensus of the disrupted BP motif (1: YTNAY, 2: YTNA, 3: TNA, and 4: YNA). (*F*) Number of data sources supporting the disrupted BP. (*G*) Population variation of the disrupted BP. (*H*) Conservation scores of the disrupted BP. (*I*) Missplicing scores and deleteriousness score of the pathogenic BP variants. (*J*) BPHunter scores of the pathogenic BP variants.

We deployed BPHunter retrospectively on the 48 reported pathogenic BP variants and successfully captured 40 of them (Fig. 4*A*, Table 2, *SI Appendix*, Fig. S17, and Dataset S4). There were 4 deletions (8/17/20/22 nt) that removed the entire BP motif, 2 single-nucleotide deletions that removed one BP site and one BP−2 site, respectively, 22 SNVs that disrupted BP sites, 11 SNVs that disrupted BP−2 sites, and 1 SNV that disrupted a BP−1 site (Fig. 4*B*). In 24 cases, the variants disrupted the only BP in their 3′-proximal introns, in 9 cases, the variants disrupted the first BP, and in 7 cases, the variants disrupted the nonfirst BP (Fig. 4*C*). The affected BP comprised 19 mBP, 1 exclusively eBP and 20 exclusively cBP (Fig. 4*D*). There were 24 BP that matched the YTNAY consensus sequence, whereas the other 8, 7, and 1 BP matched the increasingly relaxed YTNA, TNA, and YNA consensus motifs (Fig. 4*E*). There were 39 BP positions supported by more than one evidence (Fig. 4*F*). No population variants were found for 38 BP positions, whereas very rare variants were noted in the other two BP positions (Fig. 4*G*). The conservation scores GERP and PhyloP were both positive for 33 BP positions and both negative for 5 BP positions (Fig. 4*H*). We also used two missplicing prediction tools [SpliceAI (40) and MMSplice (41)] and a mutation deleteriousness prediction score [CADD (42)] to evaluate these pathogenic BP variants (Fig. 4*I*). SpliceAI provides a probability score for altering the acceptor sites (the higher the score, the higher the likelihood of missplicing), and it predicted no variant above its high-precision cutoff of 0.8 (the highest was 0.74), and five variants above its medium cutoff of 0.5. MMSplice provides a score for acceptor disruption (the lower the score, the higher the likelihood of missplicing), and it predicted no variant beyond its suggested cutoff of −2 (the lowest was −1.944). CADD provides a mutation damage score which is heavily reliant on conservation-related metrics. It identified 3 variants having scores >20 (genome-wide top 1%), 26 variants having scores in 10 to 20 (top 1 to 10%), and 11 variants with scores <10. With the use of BPHunter score (introduced in a later section) we found 38/40 variants had score ≥3 (Fig. 4*J*).

### Pathogenic BP Variants That BPHunter Did Not Detect.

BPHunter detected 40/48 reported pathogenic BP variants, leaving 8 variants undetected (Table 2), all of which were located very close ([−9, −17] nt) to 3′ss and 6 of which were published before 2004 (Fig. 4*A*). The eight introns harboring these variants all have BP documented in the BPHunter, but none of these pathogenic variants disrupted the BP. Interestingly, by extracting and aligning their wild-type (wt) and mutated (mt) sequences using SeqTailor (43), we found that all of them had created an AG-dinucleotide in the region between BP and 3′ss (*SI Appendix*, Fig. S18). By comparing the MaxEnt 3′ss strength (44) between the constitutional AGs and the created AGs, we found that these eight variants created one stronger 3′ss, six slightly weaker 3′ss, and one unlikely 3′ss. These eight original studies all reported complete/partial exon skipping or intron retention, rather than de novo 3′ss at the created AGs. We therefore hypothesize that the AG-dinucleotides created in the BP-3′ss region may have promoted binding with U2AF35 that recognizes the acceptor site, thereby sterically interfering with the recognition of PPT by U2AF65 and the formation of the spliceosome complex (45, 46) (Fig. 1*B*). Thus, these eight variants should not be considered as BP variants. Rather, they should be regarded as causing aberrant splicing through the disruption of spliceosome organization on the 3′ side of the intron. BPHunter therefore successfully detected all 40 published pathogenic variants that truly disrupted BP. When these 40 variants were tested with existing cBP datasets, we found that 36, 30, and 37 can be detected using BPP, Branchpointer, and LaBranchoR, respectively. This confirms that the detection output from BPHunter is an inclusive outcome that outperforms the use of any single BP dataset.

### Comparison of the Pathogenic BP Variants with Common BP Variants in the General Population.

Focusing on SNVs at BP and BP−2 positions, we compared 33 pathogenic variants (22 variants on BP and 11 variants on BP−2) against 659 common population variants (386 variants on BP and 273 variants on BP−2, with MAF ≥1%) (*SI Appendix*, Fig. S19*A*). We found that common variants occurred significantly less frequently in 3′-proximal introns with single BP (6%) versus pathogenic variants (58%, *P* < 0.00001 by Fisher’s exact test) and were significantly more frequent in BP of rank ≥3 (28% vs. 2%, *P* value < 0.00001). Thirty-five percent of common variants were located within BP motifs that did not match any level of consensus, which was 0% in pathogenic variants (*P* = 0); 63% of common variants were in BP sites supported by only one source, which was 3% in pathogenic variants (*P* < 0.00001). By contrasting the positively GERP/PhyloP-scored BP positions with the negatively GERP/PhyloP-scored BP positions, common variants (36% vs. 54%) showed significant enrichment (*P* < 0.00001) in having negative conservation scores against pathogenic variants (79% vs. 14%). There were 66% of common variants obtained low BPHunter scores = 0∼2 (the next section), whereas only one pathogenic BP variant was scored ≤2 (*P* < 0.00001). Furthermore, we analyzed the nucleotide changes in BP and BP−2 positions separately. We compared 22 pathogenic variants against 386 common variants at BP positions (*SI Appendix*, Fig. S19*B*). A > G was the most frequent (68%) among the pathogenic BP variants (47) but not statistically significant when compared with common BP variants (51%, *P* = 0.128). We then compared 11 pathogenic variants against 273 common variants at BP−2 positions (*SI Appendix*, Fig. S19*C*). The nucleotide changes T > A and T > G were both significantly enriched in pathogenic BP−2 variants against common BP−2 variants (37% vs. 10% with *P* = 0.025 for T > A and 45% vs. 10% with *P* = 0.0038 for T > G). The nucleotide change T > C was the least frequent (18%) in pathogenic BP−2 variants although the most frequent (38%) in common BP−2 variants, albeit not significant (*P* = 0.2198). These statistics were based on the small number of reported pathogenic variants, and with additional BP variants reported in the future this analysis should be refreshed.

### Strategy for Prioritizing BP Variant Candidates.

The analysis of the published pathogenic BP variants, in concert with the analysis of common BP variants, helped us to formulate a strategy to prioritize BP variant candidates: 1) deletion of the entire BP motif, or disruption of the BP or BP−2 positions; 2) variant of the only BP in the 3′-promixal intron or the first/second BP in the 3′-promixal intron with no more than three BP; 3) variant in a BP motif matching the consensus YTNAY, while also allowing a slightly relaxed consensus; 4) variant at a BP position with more than one supporting source, regardless of its type (mBP/eBP/cBP); 5) variant at a BP position with no or rare population variants; 6) variant at a BP position with positive-scored GERP or PhyloP may be preferred; and 7) variant C > T or T > C at BP−2 could be deprioritized. We implemented a BPHunter scoring scheme to take account of these important features (Fig. 4*J* and *SI Appendix*, Fig. S20) to help to prioritize the candidate variants. The score ranges from 0 to 10, with a higher score indicating a higher likelihood of deleteriousness. We recommend a threshold score ≥3 and suggest focusing on SNVs and deletions, as the impact of insertions on splicing is difficult to predict using the current tool. The SpliceAI, MMSplice, and CADD scores could provide additional reference but should not be fully relied upon, particularly when evaluating newly identified BP variants (see our case study of a *STAT2* variant in a critical COVID-19 patient). Additionally, homozygous and hemizygous variants should be prioritized, and candidate variants in genes associated with the disease under study should be prioritized. The sequencing quality and the complexity/repeats of neighboring nucleotides need to be carefully checked. This prioritization strategy will be updated as the number of discovered BP variants increases.

### A Germline Heterozygous SNV of *STAT2* Disrupts BP and Splicing in a Patient with Critical COVID-19.

**Fig. 5.**
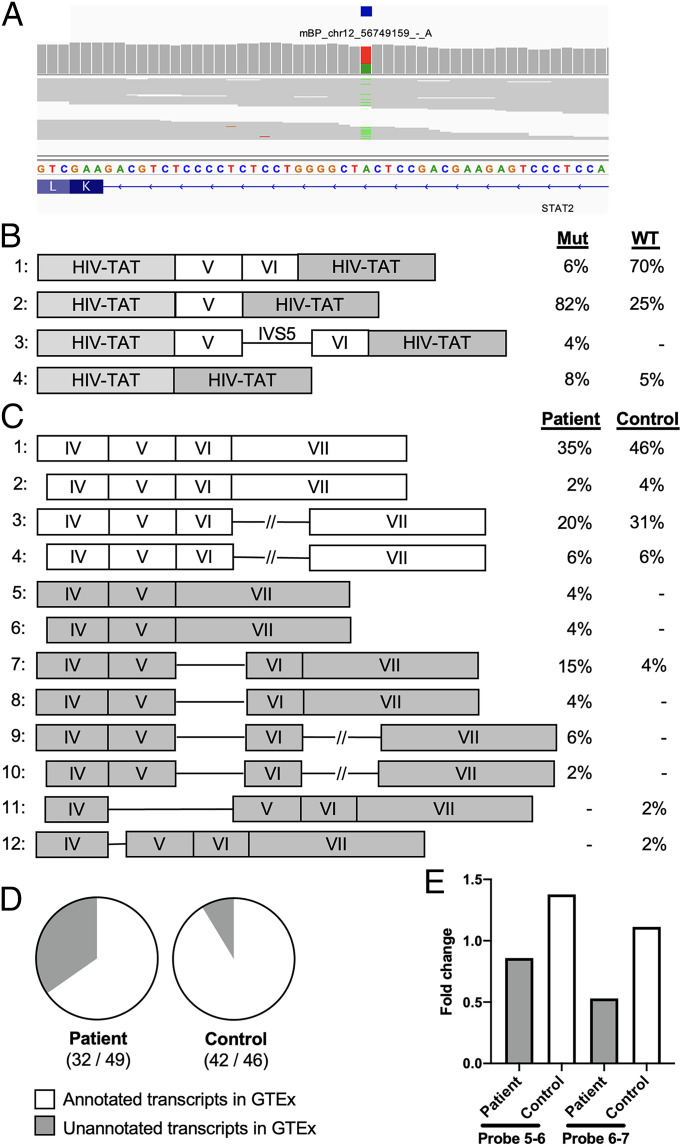
Detection and validation of a germline heterozygous *STAT2* variant which disrupts BP and splicing in a life-threatening COVID-19 patient. (*A*) Detection of a heterozygous *STAT2* variant disrupting BP. (*B*) *STAT2* transcripts and proportions from exon trapping assay in COS-7 cells. (*C*) *STAT2* transcripts and proportions from RNA extracted from whole blood. (*D*) Ratio of the GTEx-annotated transcripts among the total transcripts. (*E*) Estimation of *STAT2* canonical transcripts based on RT-qPCR, measuring *STAT2* mRNA levels in whole blood using two probes spanning intron 5 (probe 5-6) and intron 6 (probe 6-7).

We performed BPHunter on whole-exome sequencing (WES) data from a cohort of 1,035 patients with life-threatening COVID-19 (48), focusing on 13 genes that have been reported to carry deleterious variants impairing type-I interferon (IFN) production and underlying critical illness (49). BPHunter detected one heterozygous variant g.56749159T > A (IVS5-24A > T) in *STAT2* that disrupted the only BP (mBP) located −24 nt from 3′ss of intron-5 (Fig. 5*A*). This BP matched the YTNAY consensus sequence, had a MAF of 6.98e-6, exhibited a GERP of −0.257 and a PhyloP of 0.152, and was supported by five data sources. These features satisfied our prioritization strategy requirements for a promising candidate BP variant, and it had a BPHunter score = 6, even though the missplicing/deleteriousness predictions were not suggestive of its pathogenicity (SpliceAI = 0, MMSplice = −0.4, CADD = 10.1). We therefore hypothesized that this intronic variant might disrupt BP recognition, leading to missplicing of *STAT2* transcripts. To assess its impact on mRNA splicing, we performed an exon trapping assay. Compared to the wild-type control, mRNA extracted from cells transfected with this variant displayed a majority of abnormally spliced *STAT2* transcripts: 6% normal transcripts in the mutant vs. 70% in the control, 82% of complete exon-6 skipping in the mutant vs. 25% in the control, and 4% of complete intron-5 retention in the mutant vs. none in the control (Fig. 5*B*). We extracted mRNA from whole blood of the patient and a healthy control and amplified the *STAT2* complementary DNA (cDNA) from exon-3 to exon-7/8 boundary. Sanger sequencing of the PCR products following TOPO-TA cloning showed that the patient’s cells had more misspliced transcripts (exon skipping and intron retention) than the healthy control (Fig. 5*C*). The cDNA from the patient contained 35% unannotated transcripts in GTEx, whereas the control contained 9% of unannotated transcripts (Fig. 5*D*). We also assessed *STAT2* mRNA by RT-qPCR to estimate the amount of canonical mRNA transcripts extracted from whole blood and observed about half the level in the patient as compared with the control (Fig. 5*E*). If we also consider the misspliced gene products that could have been degraded by NMD (50), the patient’s cells should carry more aberrantly spliced products. This case study effectively demonstrated the identification of a novel BP variant in *STAT2* from a life-threatening COVID-19 patient and the experimental validation of its biochemical consequences. It also showed that a BP position with a very low level of evolutionary conservation can nevertheless be functional in splicing regulation, and it is for this reason that existing missplicing/deleteriousness predictions are inadequate to identify promising candidate BP variants.

### A Somatic Intronic 59-nt Deletion of *ITPKB* Removes All Three Potential BP Sites and Leads to the Retention of the Intron in a Lymphoma Patient.

**Fig. 6.**
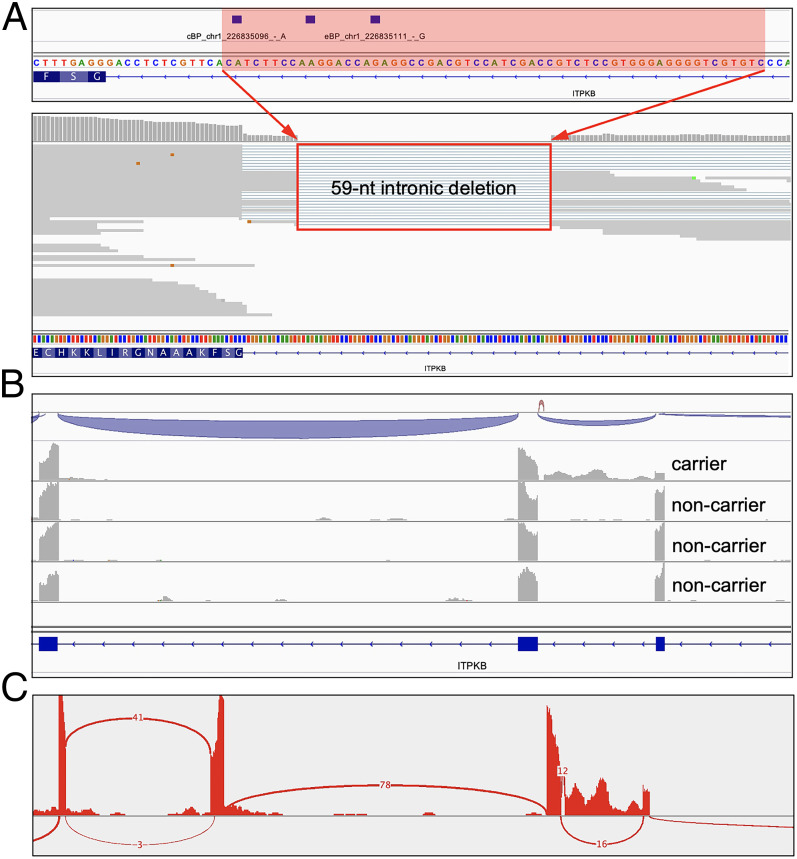
Detection and validation of a private somatic intronic deletion of *ITPKB* in a lymphoma patient. (*A*) Detection of a somatic intronic 59-nt deletion of *ITPKB* that removed all three BP sites in intron-3. (*B*) RNA-seq reads alignment on *ITPKB* that showed intronic retention in the lymphoma patient carrying this variant but not in the patients without this variant. (*C*) Sashimi plot from the RNA-seq data.

We performed BPHunter on somatic tumor-specific variant data derived from the whole-genome sequencing (WGS) of 53 diffuse large B-cell lymphoma (DLBCL) tumor samples (51–53). Focusing on a set of 212 lymphoma-associated genes (51), BPHunter detected one intronic deletion in *ITPKB* that removed 59 nucleotides from intron-3 (g.226835095_226835153del) (Fig. 6*A*). This intron contains three potential BP sites, and the deletion eliminated all of them, suggesting that the *ITPKB* gene product is highly likely to be misspliced in this tumor sample. *ITPKB* has been shown to be a negative regulator of the BCR/NF-κB signaling pathway which is very important for the growth and survival of lymphoma cells (54). It has also been shown that truncated ITPKB protein boosts PI3K/Akt signaling, which is a key growth-promoting pathway in lymphoma cells (55). We therefore hypothesized that this intronic deletion would lead to aberrant splicing, resulting in a truncated/decayed *ITPKB* gene product with loss of function/expression, leading to promotion of the growth and proliferation of lymphoma cells in the affected patient. We studied RNA-seq data of this tumor sample derived from the patient, as well as another three tumor biopsies from random DLBCL patients who do not carry this deletion, and observed complete retention of intron-3 specifically in this patient (Fig. 6 *B* and *C*). Hence, we concluded that this intronic deletion, which would have been filtered out in the absence of BPHunter, resulted in aberrant splicing of *ITPKB*, which consequently contributed to the lymphomagenesis in this patient. This case study successfully demonstrated the use of BPHunter for the detection and exploration of somatic variants. In addition, we screened the noncoding somatic variants in COSMIC (56), focused on 1,148 genes known to be associated with cancer formation and progression (*Methods*), and identified 129 candidate variants that may disrupt BP (106 SNVs and 23 deletions, in 101 genes, from 121 patients involving 19 cancer types) (Dataset S5).

### Genome-Wide Detection of BP Variants and Validation of Their Consequences from Paired WES and RNA-Seq Data.

We identified 14 individuals’ RNA-seq data that we previously published in different studies (30, 48, 57–59), whose paired WES data were also available in-house (*SI Appendix*, Table S9). We used BPHunter for genome-wide detection, retained SNVs and deletions that had BPHunter scores ≥3, removed variants that failed quality checking, and obtained 69 variants to be validated. We checked their corresponding RNA-seq data (in different cell types, under nonstimulation) and found that 39 variants in genes that have no or very low expression, which we discarded for further analysis. Of the remaining 30 variants that had sufficient RNA-seq read coverage, we observed 11 (37%) variants that demonstrated evidence of missplicing (retained intronic reads, exon skipping, and reduced splicing efficiency) (*SI Appendix*, Fig. S21). Cell type-specific missplicing was observed with variant #5, where intronic retention was evident in neurons but not in fibroblasts. It is worth noting that the RNA-seq data used for this validation were generated by different configurations (protocols, cell types, read lengths/depths, and machines), and no NMD inhibitor was used to retain misspliced transcripts. This analysis demonstrated additional BP variants that lead to missplicing consequences at the biochemical level.

## Discussion

Intronic sequences comprise ∼38% of the human genome, whereas exonic sequences comprise ∼3%. In the search for candidate variants underlying human disease, current approaches mostly focus on nonsynonymous variants in coding regions and variants residing in essential splice sites. If an investigator wishes to screen for candidate variants among the vast number of intronic variants, a combination of approaches may be applied, e.g., testing for enrichment of intronic variants in a case-control study, computing and ranking the deleteriousness/missplicing scores (40–42) of intronic variants, referencing the MAF of population variations (32, 60), and clustering genetic heterogeneity (presumably deleterious intronic variants together with coding/splice-site variants) underlying physiological homogeneity (61). However, enrichment of intronic variants relies on having a group of carriers in cases versus controls, so individual variants (albeit functionally impactful) in sporadic cases are rarely captured. Moreover, many variant deleteriousness scores (42) are heavily reliant upon evolutionary conservation, and splicing has been shown to evolve rapidly with significant differences between species (36, 37). This suggests that the application of conservation-derived deleteriousness scores to intronic splicing regulatory elements may not be particularly effective, a conclusion supported by our *STAT2* case study. We also showed that missplicing scores (40, 41) are underpowered to prioritize promising BP variants. Further, even though some intronic variant candidates may be identified, they do not come with the biochemical interpretations that other types of variants do (e.g., missense, stop-gain, essential splice site) to guide investigators in assessing their pathogenicity.

With BPHunter, we are now able to detect intronic variants that may disrupt BP recognition, and hence splicing and gene expression/function, in a systematic, efficient, and informative manner. It provides an opportunity for medical genetics studies to explore patients’ MPS data more deeply, by identifying candidate BP variants in known disease-associated genes, or alternatively in new and functionally promising candidate genes. It also provides an opportunity to systematically investigate the biomolecular effect of BP variants, at least in some genes, particularly in those 3′-proximal introns containing multiple BP. Such studies will delineate the intron-specific molecular associations between BP and splicing. We expect an increasing number of characterized BP variants, which alongside the characterization of their biochemical consequences and pathophysiological impact which will help us to comprehend BP function and dysfunction, from the positive results standpoint. BPHunter also provides a platform to report BP variants that do not give rise to missplicing, from the standpoint of negative results, which cannot be published but should nevertheless not be neglected. Lessons learned from negative BP variants will feed back valuable input that can be used to determine functional BP in different contexts, which will in turn improve our understanding of genuine BP positions and alternative BP usages, while improving our ability to discover pathogenic BP variants. We have also noted a study of 120 human BP variants (some were their own data, and some were collected from the published literature), in which the authors showed 82 BP variants (from 79 genomic positions) having no missplicing effect (62). We found that 72/79 of these positions were not documented as BP by BPHunter. Moreover, it should be noted that many of the variants in this paper were misquoted from their cited origins and had no clear experimental validation.

In this study, we built a comprehensive database of human BP which represents a qualitative and quantitative knowledgebase of BP in the human genome. This BP database is, however, not yet optimal. The eBP are likely to include false-positive BP, caused by mislocation from RNA-seq reads. The cBP would have missed false-negative BP (some real non-adenine-BP for example), as machine-learning methods always learn the major distinguishing features from training data to differentiate between positives and negatives. Regarding the BP consensus sequence, we noted that while only ∼40% of BP motifs matched the long-established YTNAY, most of the remaining BP motifs fitted with relaxed consensus patterns. Our hypothesis I is that BP motifs that fit the relaxed consensus (mostly displaying low binding affinity with snRNA) may have other *cis*-elements (e.g., intronic splicing enhancers) in the vicinity that interact with *trans*-elements with stronger affinity, to guarantee the collaborative organization of the spliceosome necessary for correct splicing (6). Our hypothesis II is that the definition of the BP consensus sequence could be context-specific (e.g., tissue-, cell-type-, developmental-stage-, or intron-specific or RNA-structure-dependent) to some extent, to meet the highly context-specific physiological requirements of different tissues and cells. A variant that creates a new BP may disrupt splicing as well, but BPHunter cannot detect this in its current form, which suggests one opportunity for improvement. In addition, we are aware of some splicing mechanism studies involving BP: recursive splicing within the intron allowing multistep intron removal (9), stem-loop structures bringing distal BP closer to 3′ss (10), stochastic splice site selection leads to kinetic variation in intron removal (11), closer-to-5′ss BP leading to mutually exclusive splicing (63), and BP haplotypes associated with alternative splicing (64). Taken together, these studies present a fascinating glimpse of the complexity of the spliceosome that calls for greater effort in understanding splicing and in investigating splicing-related pathogenesis.

BPHunter enables the efficient genome-wide detection of candidate BP variants in MPS data. We hope that this tool will help to improve our ability to diagnose human genetic disease. We also anticipate the application of BPHunter to the study of human somatic variants, in cancers for example (65). Our timely recruitment of BPHunter to screen our COVID-19 cohort directly led to the identification of a novel and deleterious BP variant in the *STAT2* gene from a patient with life-threatening COVID-19, which was immediately tested experimentally to demonstrate its biochemical consequences. Our subsequent application of BPHunter to lymphoma patients also directly led to the detection of an intronic deletion of *ITPKB* that removed all three potential BP sites in that intron leading to its complete retention as evidenced by RNA-seq data. Without BPHunter, neither of these deleterious variants that impaired the function of these key genes in the disease studied would have been picked up by preexisting conventional methods (e.g., missplicing predictions, deleteriousness scores, enrichment tests). BPHunter therefore constitutes not only an important resource that provides us with an improved genome-wide understanding of BP but also a powerful tool that should drive us to discover new BP variants underlying human genetics diseases and traits.

## Methods

### Collection of Eight Published BP Datasets, Creation of Two New BP Datasets, and Consensus-Guided Positional Adjustment of BP.

We collected five datasets of experimentally identified BP (5, 15–18) and three datasets of computationally predicted BP (19–21). We created eBP_BPHunter, by analyzing RNA-seq data of three *DBR1*-mutated patients (30). We created cBP_BPHunter, by developing a high-precision machine-learning model to scan the region [−3, −40] nt of 3′ss. We screened a window of [−2, +2] nt from each raw BP position for consensus sequence matching and adjusted the position to its closest neighbor that perfectly matched the consensus.

### Human Population Variants.

We obtained human genetic variants from the gnomAD database (32) v3.1, which contained 76,156 WGS samples. We extracted the total allele count (AC) and MAF of population variants, which were categorized into singleton (AC = 1), rare (MAF < 1%), and common (MAF ≥ 1%).

### Variant Deleteriousness and Missplicing Prediction Scores.

We used CADD v1.6 (42), which predicts variant deleteriousness. We used two missplicing scores, SpliceAI (40) and MMSplice (41), to evaluate the predicted probability of BP variants in disrupting splicing.

### A Cohort of Patients with Critical COVID-19 and a Cohort of Lymphoma Patients.

We used the WES data from 1,035 patients with critical COVID-19, which were recruited through The COVID Human Genetic Effort (48). We also used WGS data from 53 diffuse large B-cell lymphoma patients, whose paired RNA-seq data from the tumor tissues were available (51–53).

Written informed consent was obtained in the country of residence of each patient, in accordance with local regulations and with institutional review board (IRB) approval. Data analysis was conducted under the approval of the IRB of the Rockefeller University Institutional Review Board in New York.

The full methods section is available in *SI Appendix*.

## Supplementary Material

Supplementary File

Supplementary File

Supplementary File

Supplementary File

Supplementary File

Supplementary File

Supplementary File

## Data Availability

BPHunter datasets and software are publicly accessible from https://hgidsoft.rockefeller.edu/BPHunter (66) and https://github.com/casanova-lab/BPHunter (67), under CC BY-NC-ND 4.0 License. All other study data are included in the article and/or supporting information.
