# Supplementary material for: Genome-wide detection of human variants that disrupt intronic branchpoints
Source: Proc Natl Acad Sci U S A. 2022 Oct 28;119(44):e2211194119. doi: 10.1073/pnas.2211194119 (PMC9636908; doi:10.1073/pnas.2211194119)
Supplement: Supplementary File [file pnas.2211194119.sapp.pdf]

PNAS

Supplementary Information for

Genome-wide detection of human variants that disrupt intronic branchpoints

Peng Zhang*, Quentin Philippot, Weicheng Ren, Wei-Te Lei, Juan Li, Peter D. Stenson, Pere Soler Palacín, Roger Colobran, Bertrand Boisson, Shen-Ying Zhang, Anne Puel, Qiang Pan-Hammarström, Qian Zhang, David N. Cooper, Laurent Abel, Jean-Laurent Casanova*

* Corresponding Authors:

Peng Zhang (pzhang@rockefeller.edu)

Jean-Laurent Casanova (casanova@rockefeller.edu)

This PDF file includes:

Supplementary Methods
Figures S1 to S21
Tables S1 to S10
Box S1
SI References

Other supplementary materials for this manuscript include the following:

Datasets S1 to S6

SUPPLEMENTAL METHODS

Experimentally identified BP (eBP) data

Five datasets of experimentally identified high-confidence BP were collected from five large-scale studies. These five datasets were named after the last names of their first authors: eBP_Mercer (1), eBP_Taggart (2), eBP_Pineda (3), eBP_Talhouarne (4) and eBP_Briese (5) (**Table 1**). The first four datasets were derived from RNA-seq data: eBP_Mercer was identified from RNA-seq data from 11 cell lines (GSE53328) (1), eBP_Taggart was identified from Mercer's RNA-seq data and ENCODE RNA-seq data from 99 cell lines (GSE30567) (2), eBP_Pineda was obtained from 17,164 RNA-seq data sets from GTEx and TCGA (3), whereas eBP_Talhouarne was acquired from RNA-seq data of cytoplasmic RNA from 5 cell lines (PRJNA479418) (4). However, these previous RNA-seq studies either only described their processing steps without providing the tool, or provided a perl program with insufficient documentation. The R package LaSSO (6), designed for lariat and BP detection from RNA-seq data, did not work in our test. The fifth dataset eBP_Briese was detected by spliceosome iCLIP experiment in 40 cell lines (E-MTAB-8182) (5).

Identification of BP from RNA-seq data from *DBR1*-mutated patients

We obtained 15 RNA-seq datasets from the fibroblasts of three *DBR1*-mutated patients with brainstem viral infection under five different stimulation conditions (non-stimulation (NS), IFNα, pIC, HSV1-8h, and HSV1-24h) (SRP130621), which we previously studied (7). *DBR1* encodes the only known lariat debranching enzyme. This RNA-seq dataset is paired-end 150-bp long, and each sample contains around 70 million reads. We mapped the fastq reads onto the human reference genome GRCh37 with STAR aligner v2.7 (8), and outputted the unmapped reads to a new fastq file for each sample. We used Trimmomatic (9) to remove the low-quality reads and to trim the low-quality ends, to obtain the remaining reads for BP searching. To this end, we developed our one-line command Python program (BPHunter_fastq2BP.py) embedded with BLAST+ (10), to identify 5'ss-BP junction reads and hence BP positions (**Figure S2a, Data and Software Access**). Based on the GENCODE human reference genome GRCh37 and its gene annotation (11), we extracted the 20-nt intronic sequences downstream of 5'ss (20-nt 5'ss library), and the 200-nt intronic sequences upstream of 3'ss (200-nt 3'ss library), for introns longer than 200 nt. For introns shorter than 200 nt, we used the entire intronic sequences in the 200-nt 3'ss library. We first aligned all BP-searching reads to the 20-nt 5'ss library, retaining the reads that had a perfect 20-nt match or only one mismatch as 5'ss-hit reads. For each 5'ss-hit read, we trimmed away the read sequence from the start of its alignment to the 20-nt 5'ss library, and inverted the remaining sequence. We then aligned the trimmed 5'ss-hit reads to the inverted 200-nt 3'ss library, and retained those reads that had at least a 20-nt alignment with at least 95% identity in the same intron, as 5'ss-3'ss-hit reads. The ends of the aligned sequence in the 200-nt 3'ss library were used to determine the genomic positions of BP. This process yielded a total of 280,899 5'ss-BP junction reads from 15 RNA-seq datasets, harboring 8,682 unique BP positions (**Table S1**).

Consensus-guided positional adjustment of BP

Since the transesterification reaction between 5'ss and BP generates a noncanonical 2'-to-5' linkage, and the reverse transcriptase in RNA-seq can introduce variants (mismatches, micro-insertions/deletions) when traversing the 5'ss-BP junction (1, 2), we anticipated that a number of eBP sites might have been mis-located in the raw dataset (**Figure S2b**). We therefore screened a window of [-2, +2] nt from each BP for consensus sequence (YTNAY) matching, and adjusted the raw BP position to its closest neighbor that perfectly matched the consensus (**Figure S2c**).

Computationally predicted (cBP) datasets

We also collected three datasets of computationally predicted BP, and we named these three datasets after their method names: cBP_BPP (12), cBP_Branchpointer (13) and cBP_LaBranchoR (14) (**Table 1**). cBP_BPP was trained by using expectation maximization algorithm on eBP_Mercer data, and predicted BP in the 14-nt region [-21, -34] nt of 3'ss (12). cBP_Branchpointer was trained by gradient boosting machine on eBP_Mercer data, and predicted BP in the 27-nt region [-18, -44] nt of 3'ss (13). cBP_LaBranchoR was trained by sequence-based deep-learning on eBP_Mercer and eBP_Taggart data to predict BP in the region [-1, -70] nt of 3'ss (14).

Prediction of BP in the region [-3, -40] nt upstream of 3'ss

As the previous BP predictions only used one or two eBP datasets for training, and overlapped the prediction in the region [-21, -34] nt of 3'ss, we supplemented them with an additional high-precision BP prediction in the region [-3, -40] nt of 3'ss that covered the sequences closer to 3'ss. We used all 198,256 consensus-guided position-adjusted eBP as positive training data (**Results**), and randomly generated 1,000,000 non-BP positions from intronic and exonic regions as negative training data. We extracted the flanking 13-nt motif [-9, +3] nt of each BP and non-BP position in the training data, based on the interaction mode between BP and snRNA (15) (**Figure 1b**). We then vectorized the 13-nt motif into 52-bit binary code by one-hot-encoding (converting A to 0001, C to 0010, G to 0100, and T to 1000). We developed three machine learning classification models: gradient boost machine (GBM), random forest (RF), and logistic regression (LR), by using scikit-learn (16) (**Figure S2d**). For each model, we first performed parameter optimization by using grid search of different combinations of key parameters, and evaluated the performance of each set of parameters by stratified-shuffled 10-fold cross-validation based on its F1 score (F1 = 2 * (precision * recall) / (precision + recall)). The parameters yielding the highest F1 score were selected as the optimal parameters (**Table S10**). As we aimed to identify the BP candidates with high precision, we performed thresholding optimization to establish the optimal probability cutoff for each model. We generated precision-recall curves (PRC) and receiver operating characteristic curves (ROC), by averaging the performance of stratified-shuffled 10-fold cross-validation. We determined the optimal threshold of each model by requiring precision ≥ 0.95 and maximizing Youden's J statistic (J = sensitivity + specificity - 1). We therefore trained and optimized GBM-BP model, RF-BP model and LR-BP model respectively, and then combined them by majority voting for improved performance (**Table S10**). We then extracted all positions in the region [-3, -40] nt of all 3'ss, and vectorized their flanking 13-nt motif into binary code as input for BP prediction.

Intronic data

We obtained human genome sequence and gene annotation data on the hg19/GRCh37 genome assembly from the GENCODE database (11). By focusing on protein-coding genes and transcripts, and requiring the gene/transcript status = 'KNOWN' and the confidence level = "1 or 2", we extracted a total of 43,225 transcripts from 19,149 protein-coding genes. We identified multi-exon transcripts and removed introns that were shorter than 10 nucleotides, thereby obtaining 355,472 introns (200,059 unique introns) from 41,975 transcripts of 17,372 genes (**Figure S6**). We tested the genomic overlaps between these 200,059 introns, and identified 41,952 introns with alternative splicing. We also collected 672 and 752 minor introns reported by the IAOD (17) and MIDB (18) databases respectively. We then detected 18 new minor introns by implementing the intron classification criteria proposed by MIDB (**Supplemental Data 3**). A gene ontology analysis revealed that the genes harboring minor introns were enriched in intracellular transport and ion channels. We also obtained canonical transcript data from the MANE database (19).

Mapping BP to introns

The genomic positions of all 546,559 BP and the genomic ranges of all 200,059 introns were formatted into BED files. We then mapped BP to introns using BEDTools (20), to identify all the pairwise BP-intron associations based on their positional intersection.

Nucleotide composition

We defined the region of union [-9, +3] as the BP motif. We measured the nucleotide frequency of BP, 5'ss and 3'ss motifs in major and minor introns respectively, and plotted them by using SeqLogo (21).

BP-U2/U12 snRNA binding energy

U2/U12-snRNA binds to the [-5, +3] and [-7, +2] regions of BP in major/minor introns respectively, whereas the BP site itself bulges out and is not involved in the interaction with snRNA (1, 22) (**Figure 1b**). We used the RNAcofold function from ViennaRNA package (23) to estimate the binding energy between BP motifs (excluding the BP sites) and U2/U12 snRNA sequences (U2: AUGAUGUG, U12: AAGGAAUGA), according to the associated intron type. RNAcofold allows the intermolecular base pairing between two RNA sequences to form static interactions, and computes the minimum free energy (MEF), which is always negative or equal to zero, to represent the binding energy (in unit: kcal/mol): a value close to zero denotes unstable binding, whereas a more negative value denotes more stable binding between the BP motif and U2/U12 snRNA.

Motif searching in the region [-50, +20] nt surrounding BP

We searched for enriched motif patterns potentially concealed within the 50-nt upstream regions and 20-nt downstream regions of the BP positions separated by their nucleotides (adenine-BP, cytosine-BP, guanine-BP, and thymine-BP). We performed XSTREME analysis (24) for motif discovery (motif width: 5-10 nt), and reported those enriched motifs having *p*-values <0.05 and appearing >5% in each of the nucleotide-separated BP datasets. The *p*-values were computed using the randomly shuffled nucleotides from the input sequences as the background.

Human population variant data

We obtained human genetic variants from the gnomAD database (25) v3.1, which contained 76,156 WGS datasets on the hg38/GRCh38 genome assembly. We converted the variants' genomic positions from GRCh38 to GRCh37 by using the liftover program from the UCSC Genome Browser (26), to allow a consistent presentation of all the genomic data in this article. By focusing on protein-coding genes, we obtained population variants and their total allele count (AC) and minor allele frequencies (MAF). We categorized variants into singleton (AC = 1), rare (MAF < 1%), and common (MAF ≥ 1%).

Cross-species conservation scores

We obtained the pre-computed genome-wide cross-species conservation scores (GERP and PhyloP-46way) from the UCSC Genome Browser (26). GERP (Genomic Evolutionary Rate Profiling) computes position-specific scores of evolutionary constraint using maximum likelihood evolutionary rate estimation by aligning 35 mammals (27). PhyloP-46way (Phylogenetic P-values) measures the evolutionary base-wise conservation based on the alignment of 46 vertebrates (28). Both scores indicate the strength of purifying selection of a given genomic position: a positive

value denotes that the genomic position is likely to be evolutionarily conserved across species, whereas a negative value indicates that the genomic position is probably evolving neutrally.

Variant deleteriousness, mis-splicing prediction scores, and splice site strength

We used CADD v1.6 (29), which predicts the deleteriousness of variants by taking account of an array of nucleotide sequence information and variant annotations (including conservation, amino acid change, epigenetic modification, human population variation, splicing, etc.). We extracted CADD PHRED-scaled scores (ranging from 0 to 99): the larger the score, the higher the probability of deleteriousness. It precomputed and ranked all possible variants in the human genome, and then assigned a score of 10 to the top 10% of predicted deleterious variants, a score of 20 to the top 1% of variants, and a score of 30 to the top 0.1% of variants, etc. Usually, a high-cutoff of 20 or a moderate-cutoff of 10 were used for large-scale variant filtration for deleterious candidate variants (30, 31). We also recruited SpliceAI (32) and MMSplice (33) to evaluate the mis-splicing prediction scores on BP variants. SpliceAI predicts splice junctions from RNA sequence, by means of a deep neural network model (32). It claimed its capability to recognize *cis*-acting elements (including BP), and to predict their mutational impact on splicing. SpliceAI provides a score for disrupting the acceptor site (ranging from 0 to 1): the higher the score, the higher the probability of altering the acceptor site. SpliceAI suggested a high-cutoff of 0.8 for high-precision, and also recommended a moderate-cutoff of 0.5. MMSplice predicts the effect of variants on splicing, by means of a neural network-based modular modelling on different components of splicing. It claimed to include 50 nt upstream of 3'ss to cover BP regions in training their model. MMSplice computes a score (unspecified range) for acceptor site inclusion (positive score) or exclusion (negative score). MMSplice suggested a cutoff of -2 to be considered as evidence for acceptor site disruption (33). In the study of splice site strength, we used SeqTailor (34) to extract the wild-type and mutated 23-nt DNA sequences surrounding the variants of interest, and then used MaxEntScan (35) to estimate the splice site strength in wt and mt sequences respectively.

A cohort of patients with critical COVID-19

In this study, we used the whole-exome sequencing (WES) data from a cohort of 1,035 patients with life-threatening COVID-19, which were recruited through an international consortium - The COVID Human Genetic Effort (36). All human subjects in this study were approved by the appropriate institutional review board.

Exon trapping assay

DNA segments encompassing *STAT2* exon 5 and 6 region (chr12:56749479 to chr12:56748872 region, GRCh37 reference) were amplified from genomic DNA extracted from PBMCs of a healthy control and were cloned into a pSPL3 vector, using the *EcoR*I and *BamH*I sites. c.472-24 A>T of *STAT2* (an intronic variant located in intron 5 and predicted to alter a branchpoint) was generated by site-directed mutagenesis. Plasmids containing wild-type (wt) and mutant *STAT2* exon 5 and 6 region were then used to transfect COS-7 cells. After 24 hours, total RNA was extracted and reverse transcribed. cDNA products were amplified using flanking HIV-TAT sequences of the pSPL3 vector, and ligated into the pCRTM4-TOPO® vector (Invitrogen). StellarTM cells (Takara) were transformed with the resulting plasmids. Colony PCR and sequencing using primers located in the flanking HIV-TAT sequences of the pSPL3 were performed to assess the splicing products transcribed by the wt and mutant alleles.

TOPO-TA cloning and RT-qPCR

Total RNA was extracted from a whole blood sample from the patient and a healthy control using Tempus Blood RNA Tube and Tempus Spin RNA Isolation Kit (Applied Biosystems), and reverse transcribed into cDNA using

SuperScript III (Invitrogen). For TOPO-TA cloning, specific primers located in exon 3 (forward primer, CATGCTATTCTTCCACTTCTTG) and exon 7-8 boundaries (reverse primer, GGCATCCAGCACCTCCTTTC) were used to amplify *STAT2* cDNA by PCR. PCR products were then purified and ligated into a pCRTM4-TOPO® vector (Invitrogen). StellarTM cells (Takara) were transformed with the resulting plasmids. Colony PCR and sequencing using the primers used to amplify *STAT2* cDNA were performed to assess the splicing products generated from the wt and mutated alleles. For RT-qPCR, *STAT2* mRNAs were quantified using probes Hs01013129_g1 (exons 5-6) and Hs01013130_g1 (exons 6-7; Thermo Fischer Scientific), with the Taqman Gene Expression Assay (Applied Biosystems), and normalized to the expression level of human β-glucuronidase. Results were expressed using the ΔΔCt method, as described by the manufacturer, and the amount of *STAT2* canonical transcript (ENST00000314128.9) was estimated based on the TOPO-TA data using the following formula: ΔΔCt x percentage of canonical transcript/percentage of transcripts with canonical exon 5-6 junction for probe Hs01013129_g1 or ΔΔCt x percentage of canonical transcripts/percentage of transcripts with canonical exon 6-7 junction for probe Hs01013130_g1.

A cohort of lymphoma patients

We studied the somatic variants from a cohort of 53 diffuse large B-cell lymphoma patients, whose paired WGS and RNA-seq data from the tumor tissues were also available (37-39). We focused on a set of 212 genes that are frequently mutated in B-cell lymphomas or known to be important for B-cell lymphomagenesis (37). All human subjects in this study were approved by the appropriate institutional review board.

COSMIC database

We collated the somatic variants documented in the COSMIC database (40) v94, which have been detected in cancer patients from a variety of different sources. We also identified four gene sets of interest, which were associated with cancer formation and progression: 123 tumor suppressor genes, 161 apoptosis genes, 150 DNA repair genes and 714 cell cycle genes, based on the COSMIC (40) and MSigDB (41) databases. We used the following criteria to retain the candidate BP variants: (1) in canonical transcripts; (2) deletions (< 100 nt) or SNVs that remove or disrupt the entire BP motifs or the BP/BP-2 positions; (3) in 3'-proximal introns harboring single or two BP; (4) no or very rare (MAF < 1e-3) population variations; (5) having BPHunter score ≥ 3; and (6) passed the variant quality checking.

SUPPLEMENTAL FIGURES

Figure S1: Splicing mechanisms that use distal BP deep inside an intron. (a) Recursive splicing mechanism in an intron for multi-step intron removal. (b) Stem-loop RNA structure brings distal BP closer to 3'ss. (c) Stochastic splice site selection leads to kinetic variation in intron removal. (d) Mutually exclusive splicing by using BP closer to 5'ss.

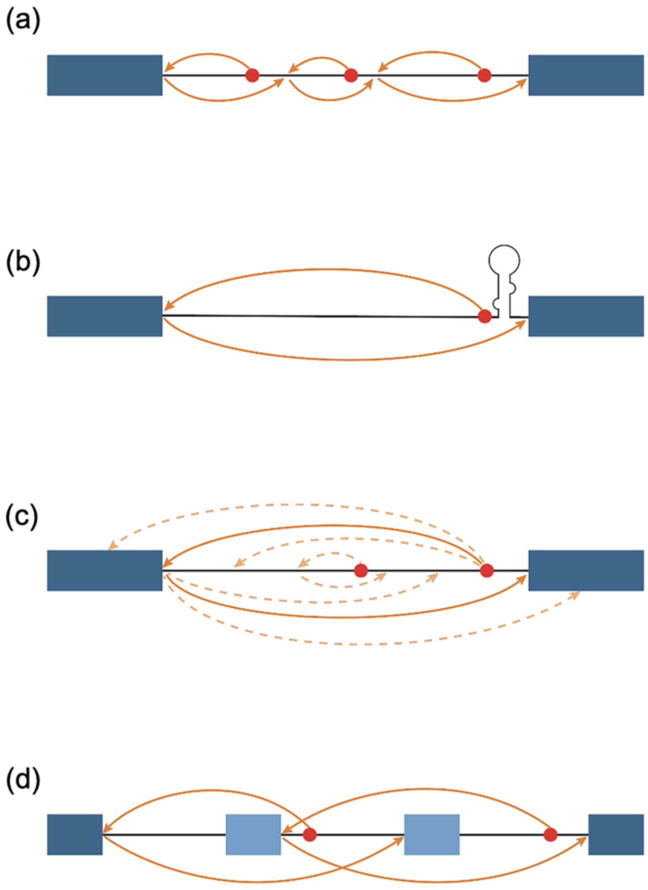

Figure S2: Identification of eBP_BPHunter, BP positional adjustment, and prediction of cBP_BPHunter. (a) Workflow of BP identification from RNA-seq of *DBR1*-mutated patients. (b) Introduction of mutations to the 5'ss-BP junction reads by reverse transcriptase in an RNA-seq experiment. (c) Positional adjustment of BP within its [-2, +2] neighborhood, guided by the consensus sequence (blue: raw position, red: adjusted position). (d) Development of three machine learning models to majority-voted prediction of BP within the region [-3, -40] nt of 3'ss.

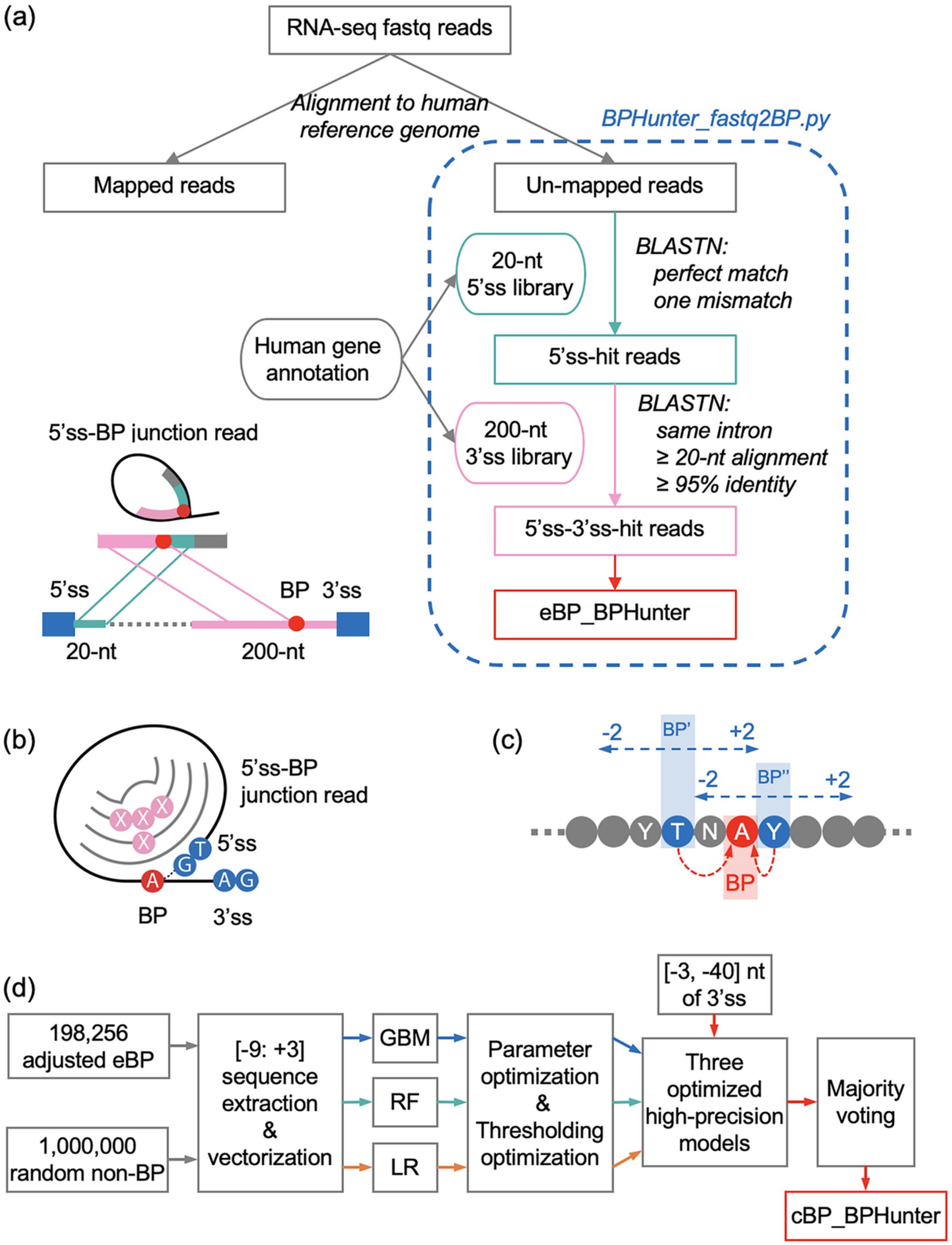

Figure S3: The overlaps between BP datasets (after consensus-guided positional adjustment). (a) The overlaps between five eBP datasets, excluding eBP_Talhouarne owing to its small data size, its high representation of cytosine-BP, and the difficulty in visualizing the overlaps of six datasets. (b) The overlaps between four eBP datasets. (c) The overlaps between the combined eBP dataset and the combined cBP dataset.

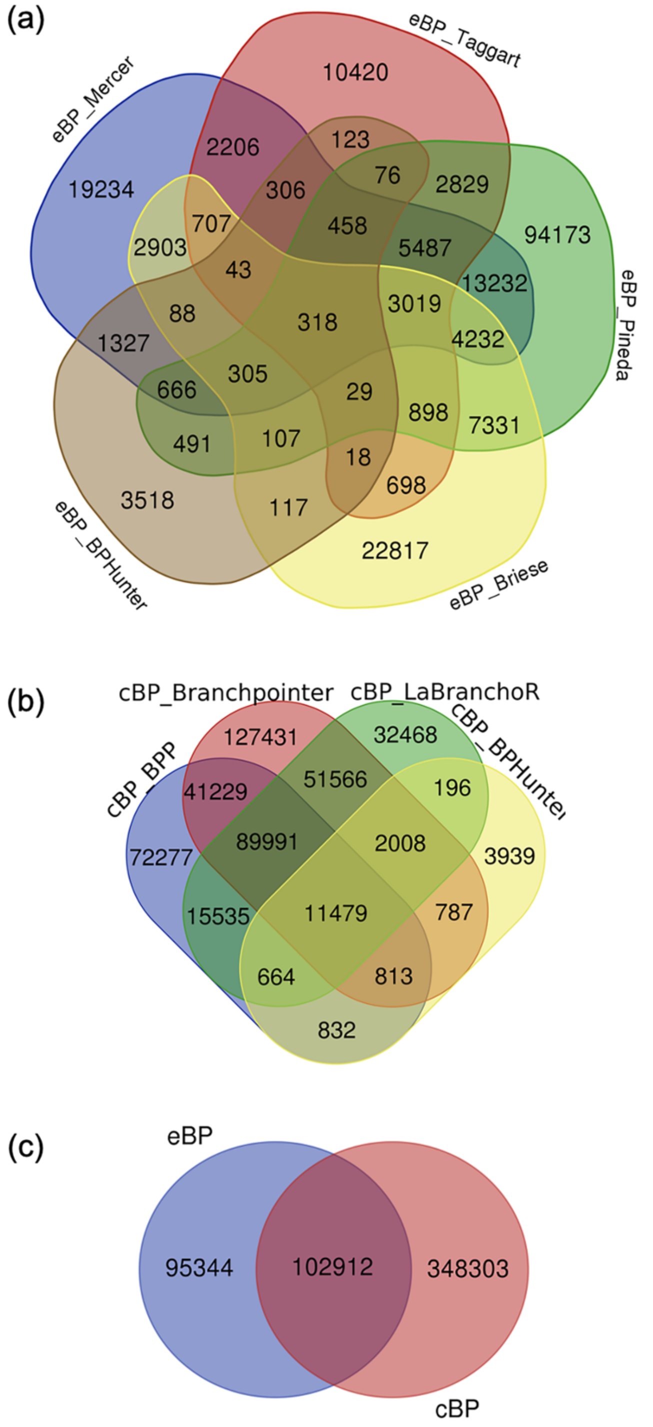

Figure S4: BP motif decomposition by their nucleotide and consensus sequences. The hierarchical decomposition of the total BP data by their nucleotides (A/C/G/T), and then by increasingly relaxed consensus sequences (1: YTNAY, 2: YTNA, 3: TNA, and 4: YNA).

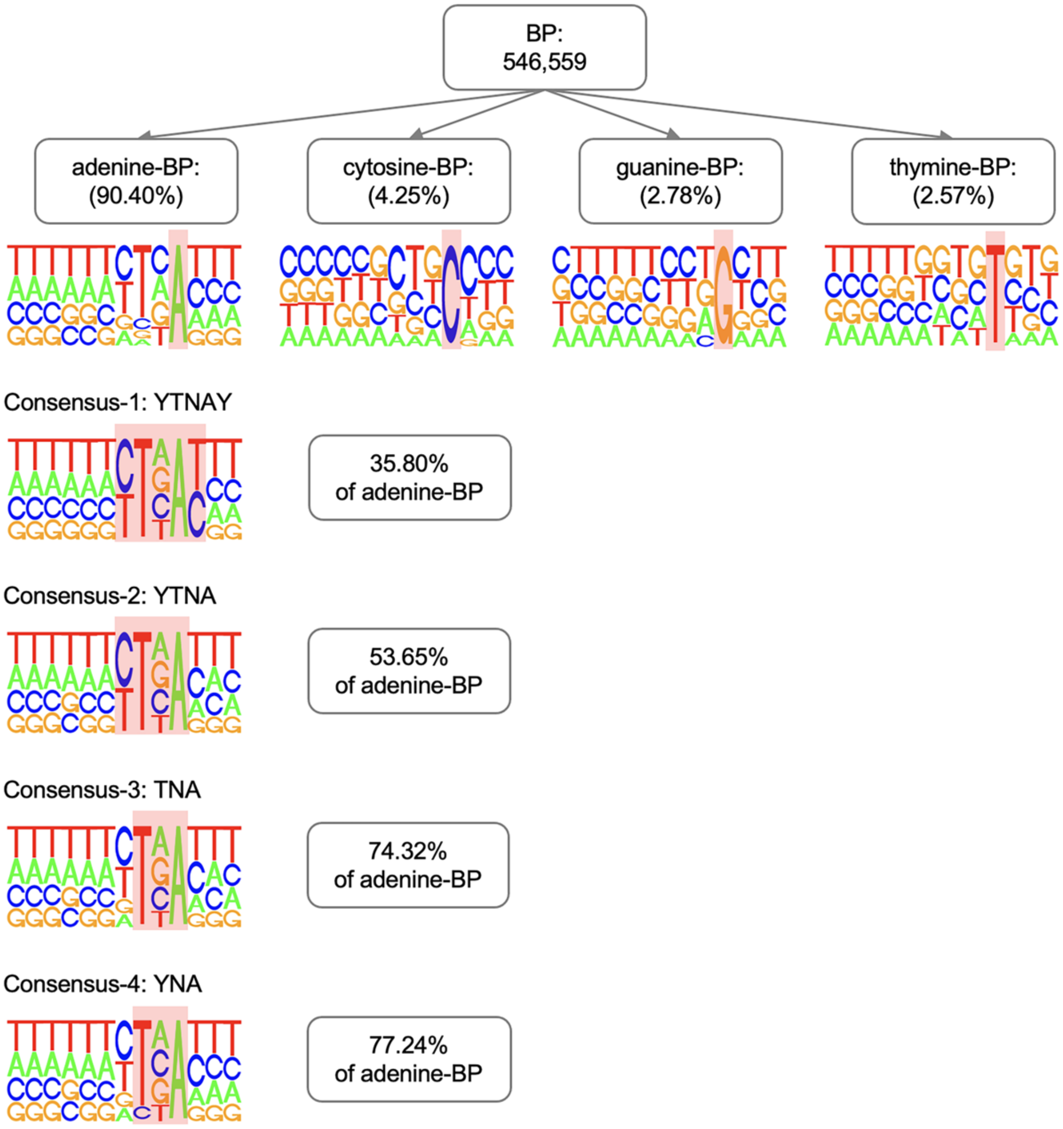

Figure S5: Motif searching in the region [-50, +20] nt surrounding BP, in terms of different nucleotides of BP. A motif was reported to be enriched if it had a *p*-value < 0.05 and $> 5\%$ occurrence in each respective BP dataset. Motif enrichment was only observed in the 50-nt upstream region of BP, whereas no motif was enriched in the 20-nt downstream region of BP.

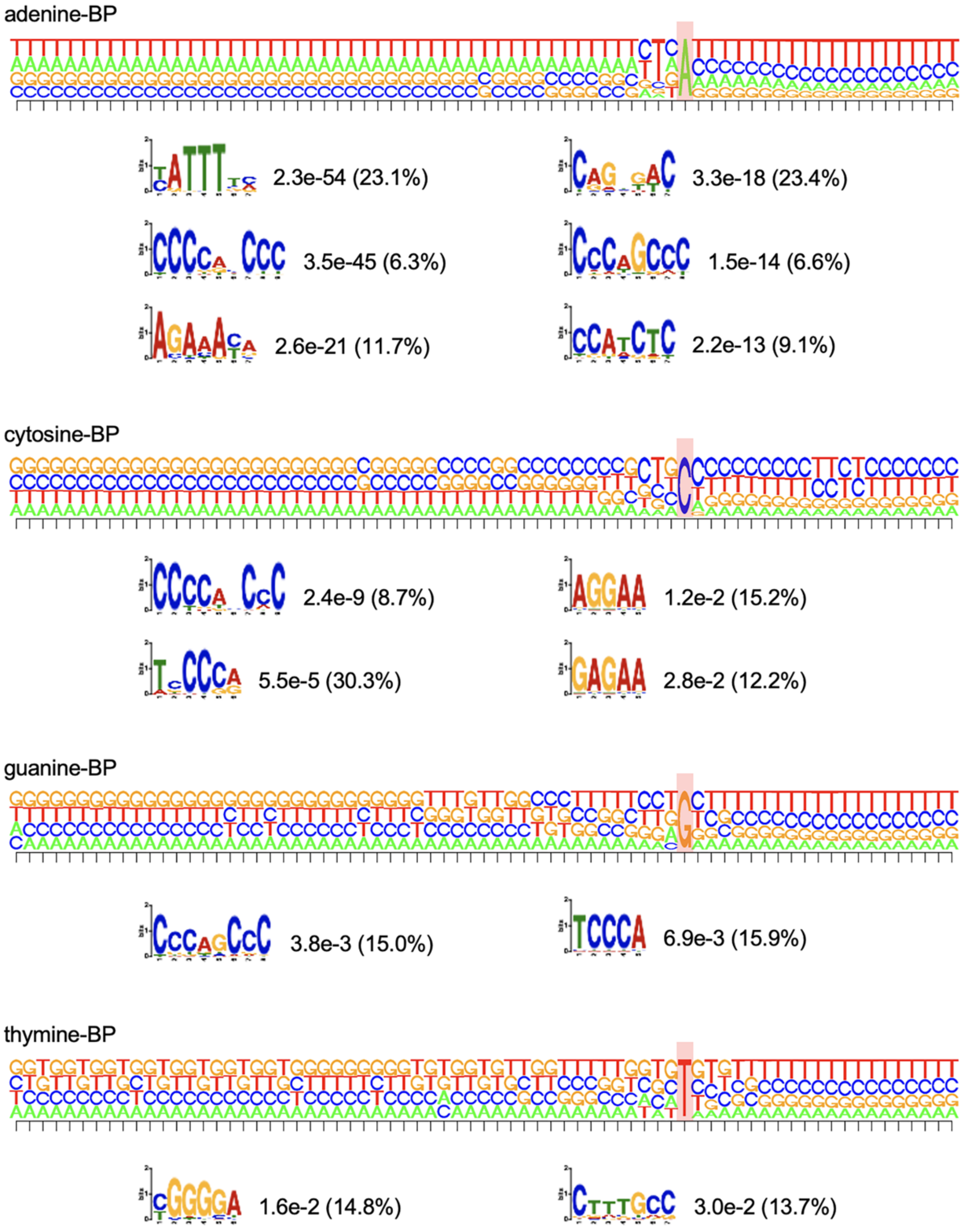

Figure S6: Collation of introns from human protein-coding genes. The reference genome and gene annotation were obtained from the GENCODE database. The introns were classified into major and minor types.

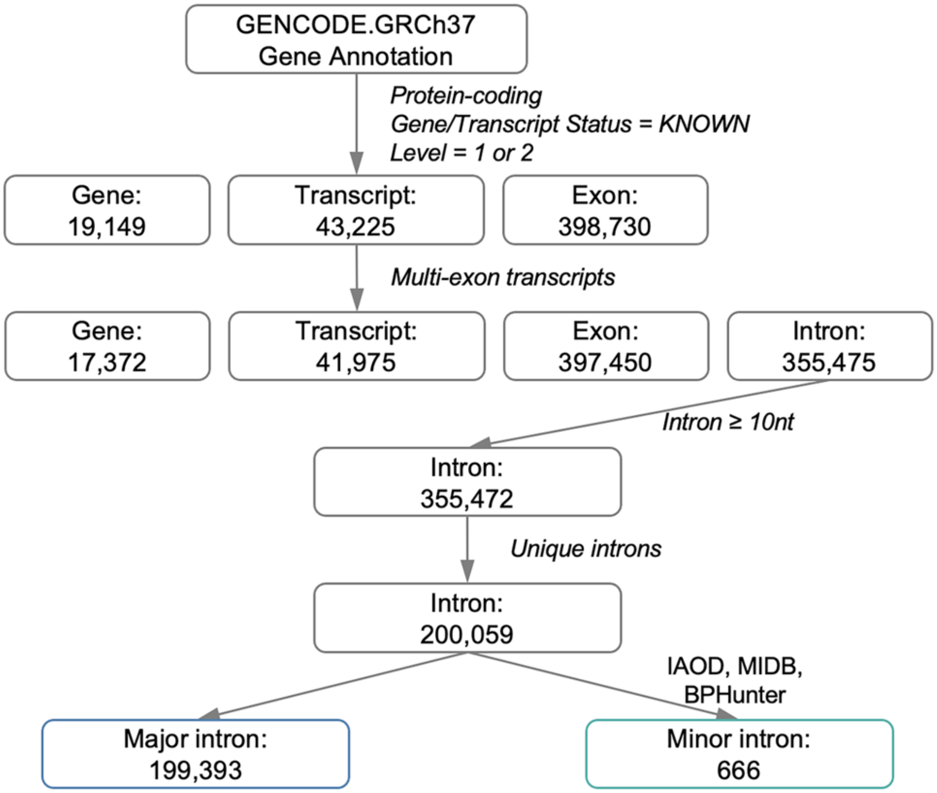

Major intron — Intron Length

Minimum	26
Medium	1,682
Maximum	1,097,903

Minor intron — Intron Length

Minimum	82
Medium	1,271
Maximum	522,714

Major intron — Top 5’ss-3’ss Pair

GT - AG	98.92%
GC - AG	0.97%
GT - TG	0.01%

Minor intron — Top 5’ss-3’ss Pair

GT - AG	71.02%
AT - AC	24.02%
AT - AG	1.80%

Figure S7: The relative distance from the non-first BP to the first BP in each intron.

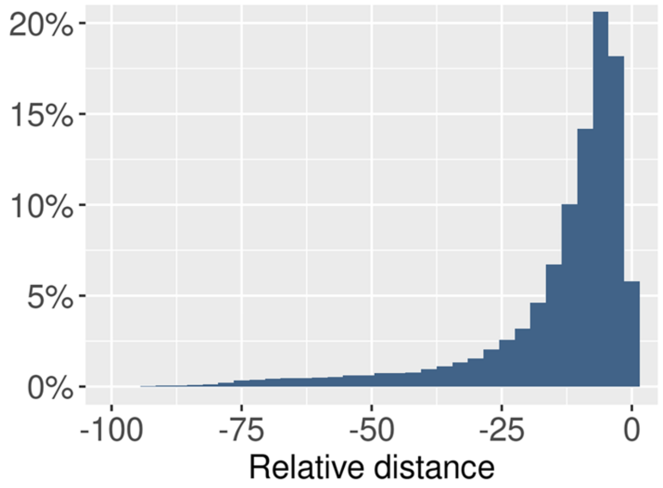

Figure S8: **The proportion of each genomic position harboring population variants (upper), and the MAF distribution of population variants (lower).** This represents an extension of the main **Figure 3d**, which includes an additional six positions (-2, +4, +5 of 5'ss, and -5, -4, +2 of 3'ss) around the splice sites.

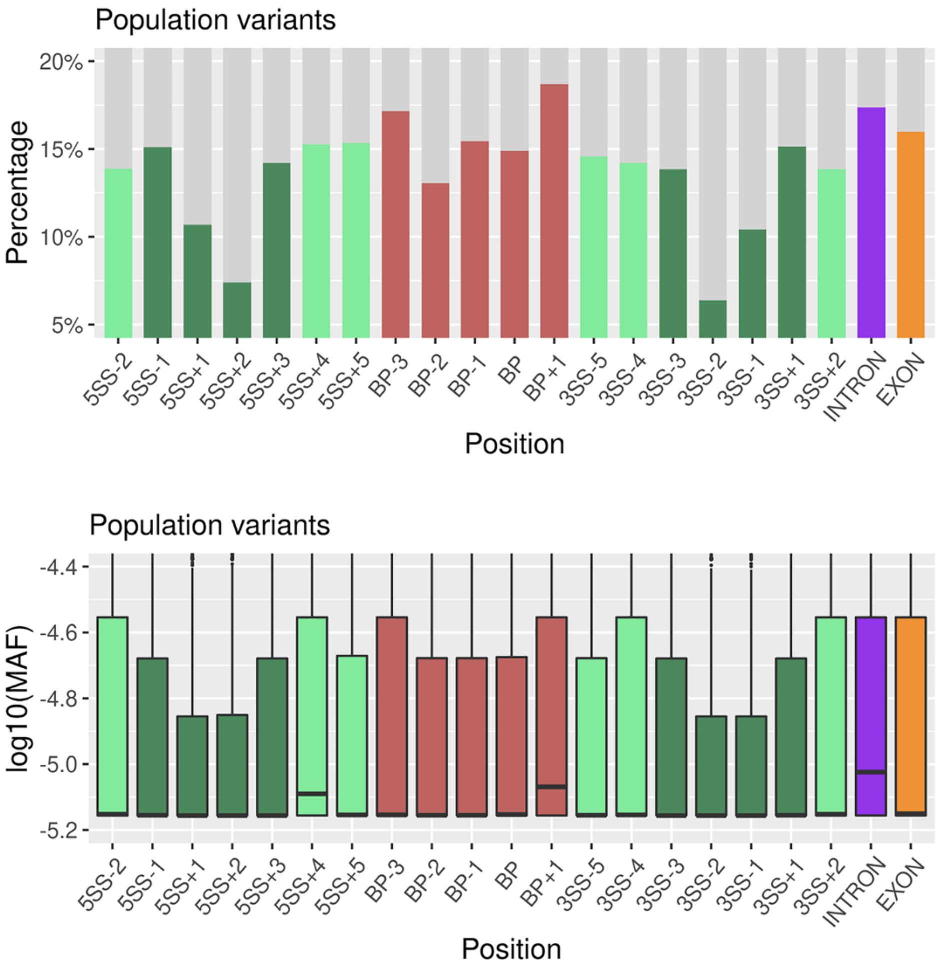

Figure S9: The distribution of the conservation scores GERP (left) and PhyloP (right) in each genomic position. The represents an extension of the main **Figure 3e**, which includes an additional six positions around the splice sites.

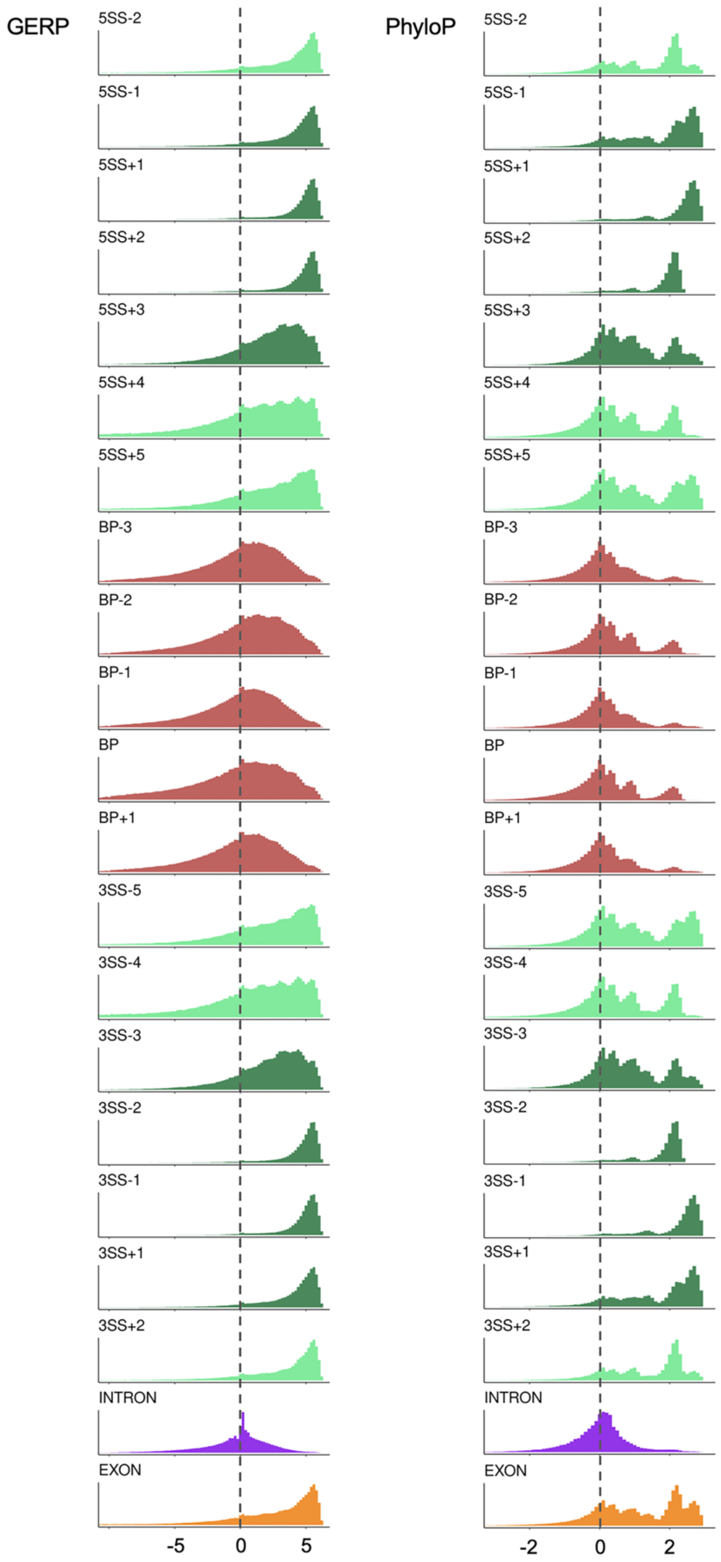

Figure S10: Comparison of BP (I): adenine-BP *vs.* cytosine-BP *vs.* guanine-BP *vs.* thymine-BP.

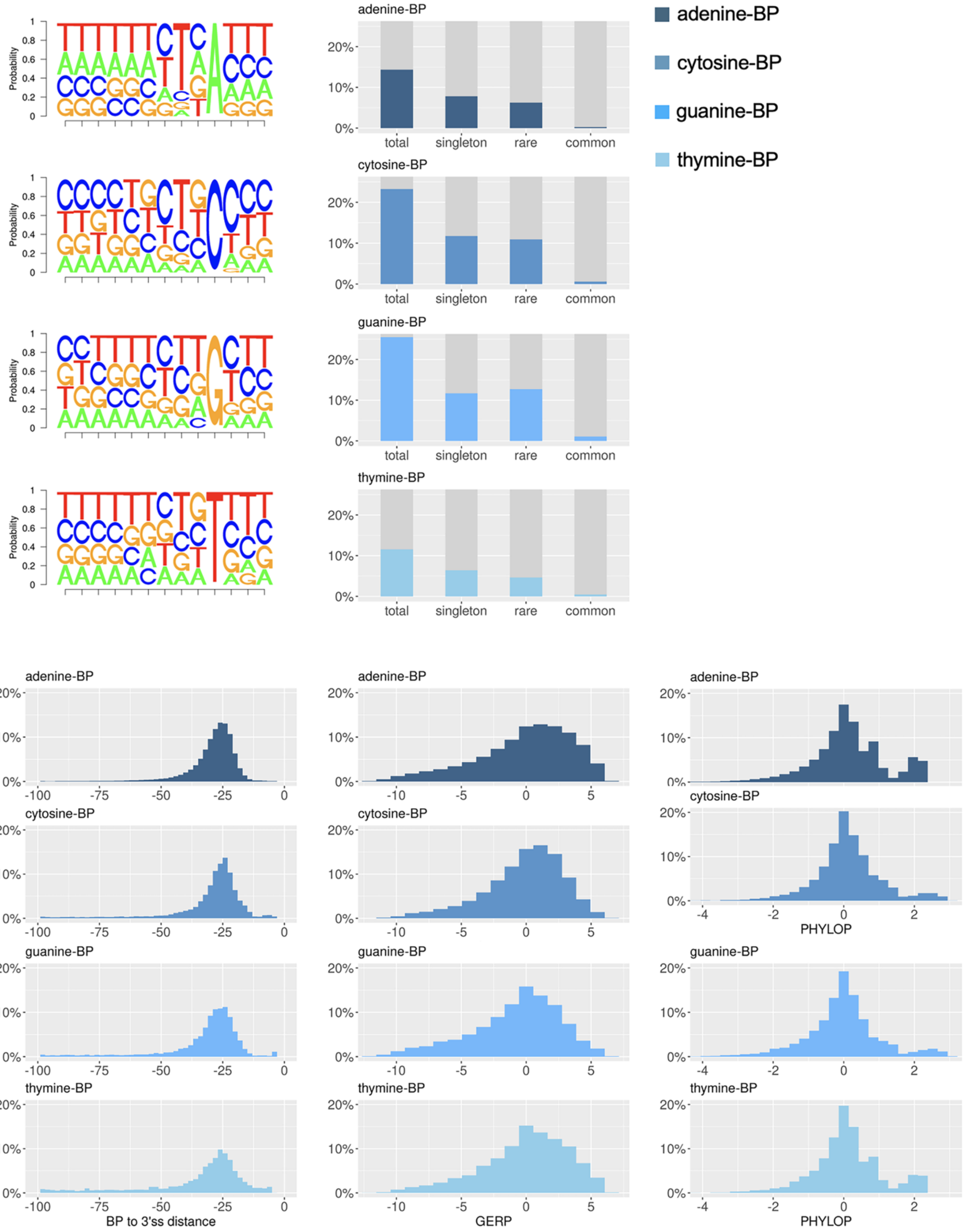

Figure S11: Comparison of BP (II): mBP *vs.* exclusively eBP *vs.* exclusively cBP.

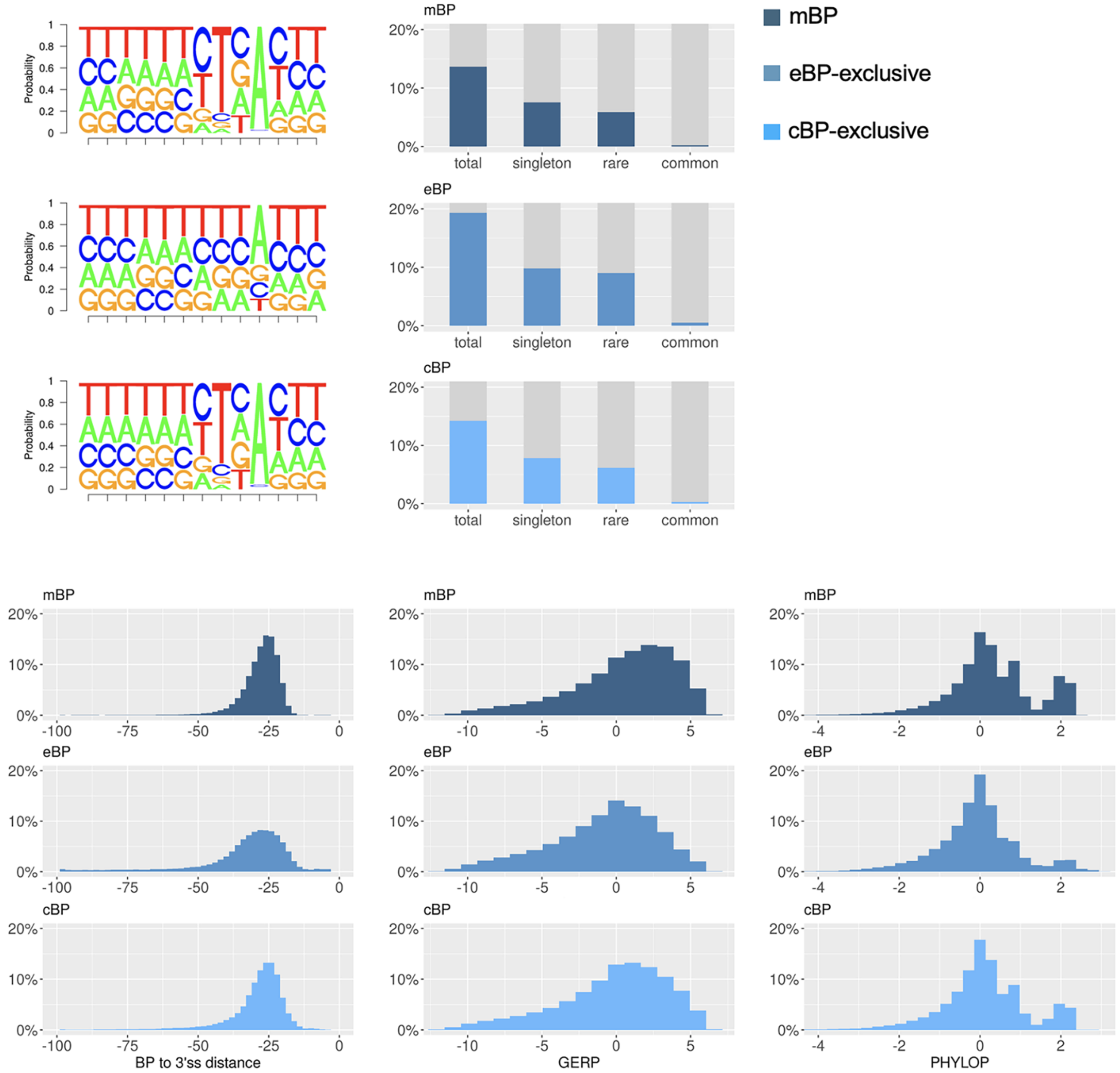

Figure S12: Comparison of BP (III): first BP in 3'-proximal introns *vs.* non-first BP in 3'-proximal introns.

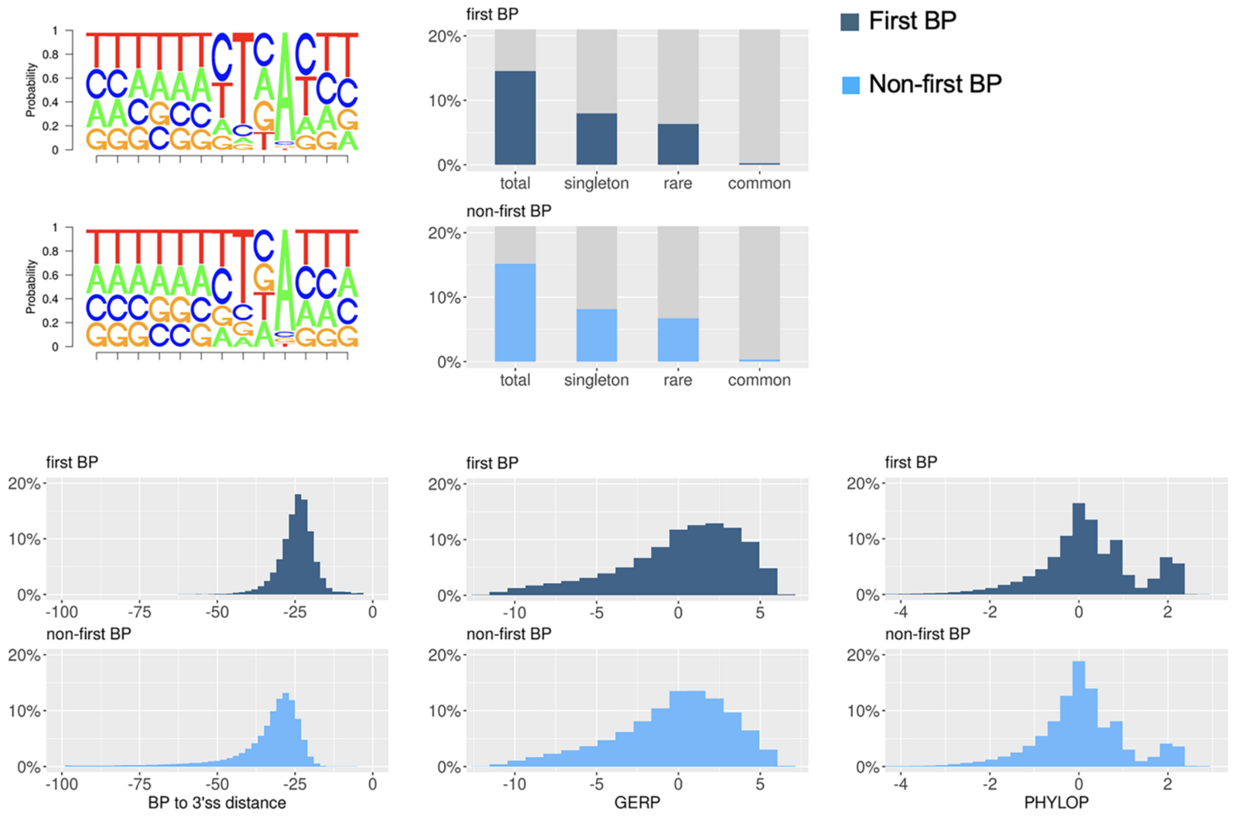

Figure S13: Comparison of BP (IV): BP in 3'-proximal introns of single BP *vs.* BP in 3'-proximal introns of multiple BP.

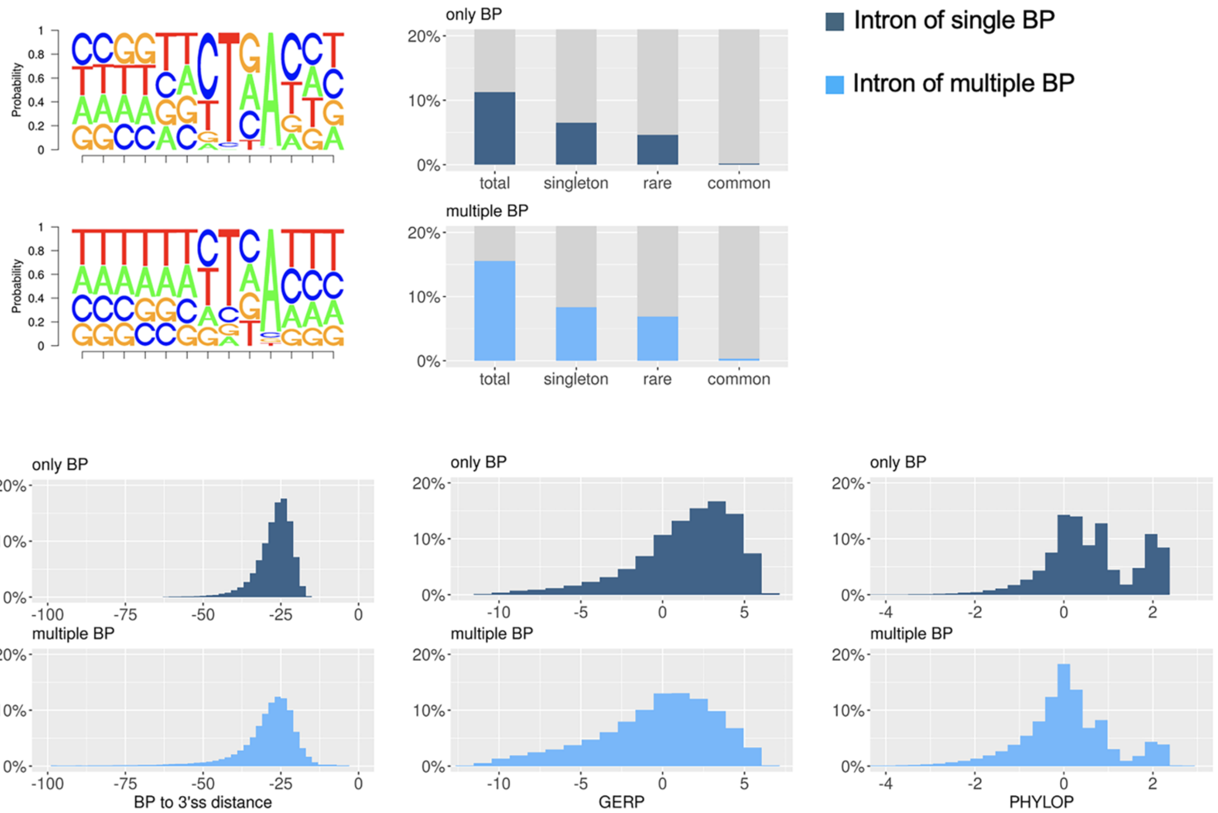

Figure S14: Comparison of BP (V): BP in non-alternatively spliced introns *vs.* BP in introns with alternative splicing.

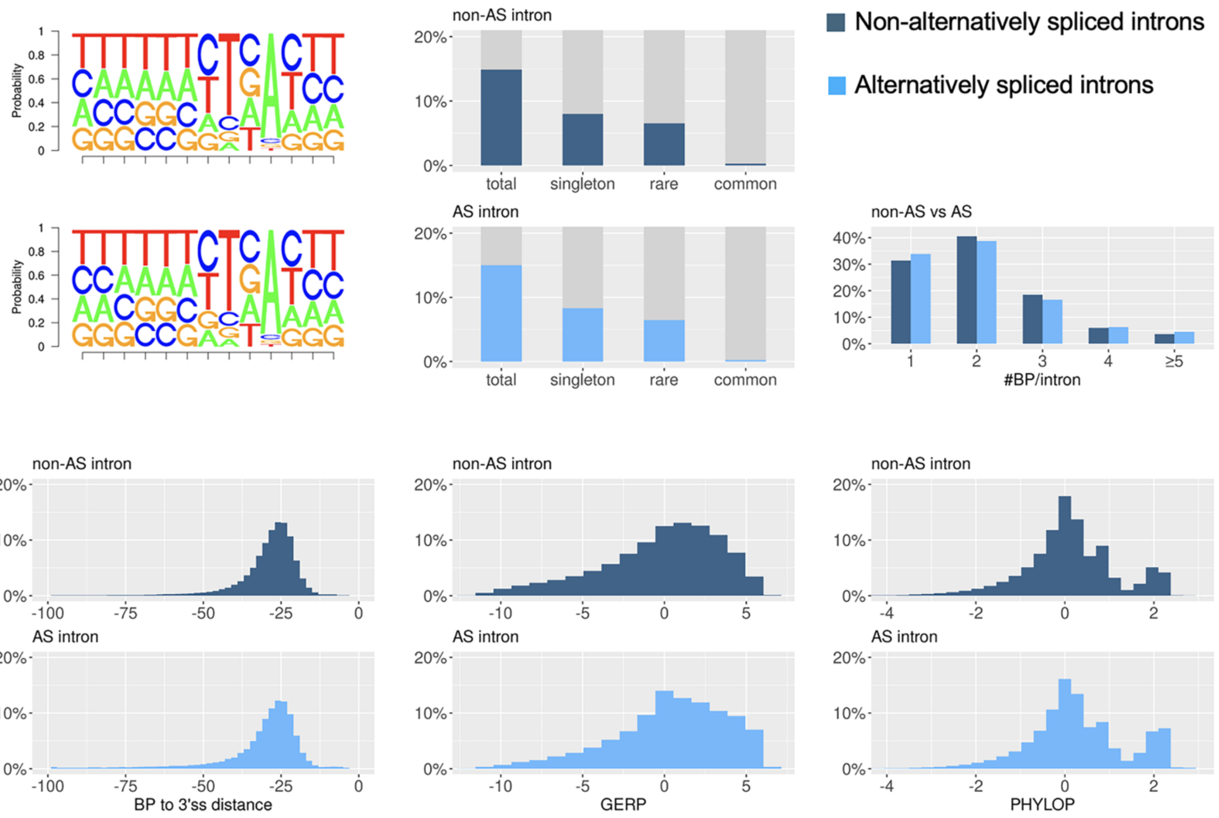

Figure S15: Comparison of BP (VI): BP in genes encoding a single isoform *vs*. BP in genes encoding multiple isoforms.

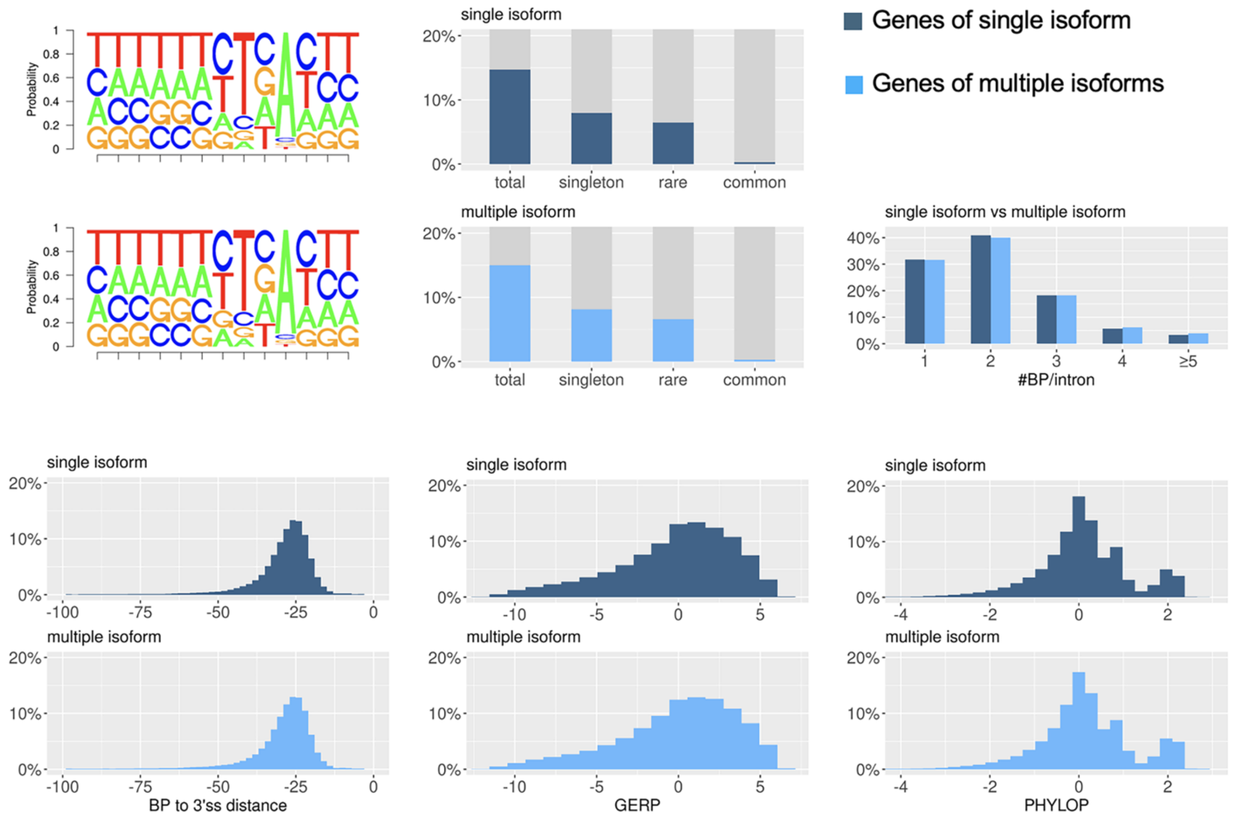

Figure S16: Comparison of BP (VII): BP in genes located on the sex chromosomes *vs.* BP in genes located on the autosomes.

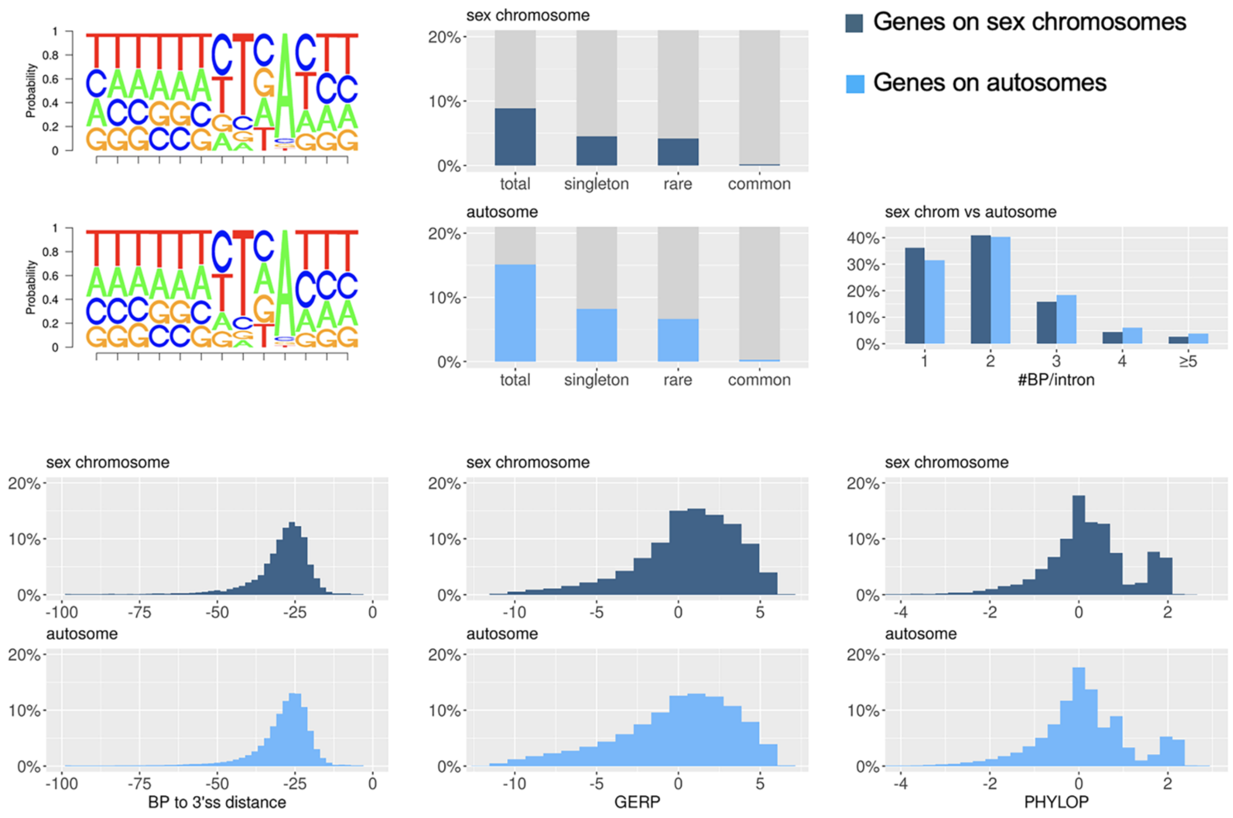

Figure S17: Graphical illustration of the 40 BPHunter-detected pathogenic BP variants. Full details of these pathogenic BP variants and BPHunter's annotation are available in **Supplementary Data 4**.

ABCC8	g.17452526T>C	IVS11-20A>G

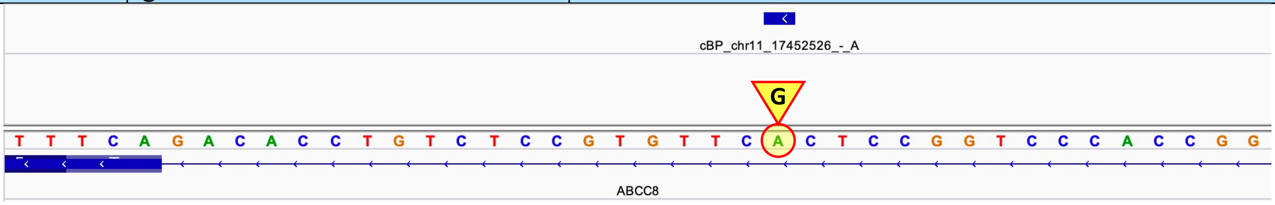

ALPL	g.21896765_21896784del	IVS7-33_-14delCCCGGCATGTGCTGACACAG

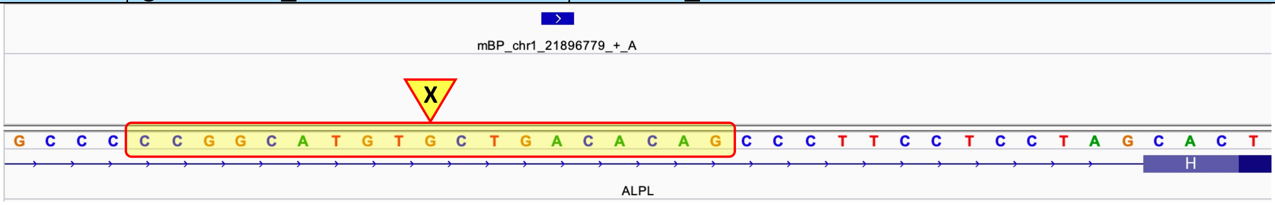

BBS1	g.66287067A>T	IVS8-21A>T

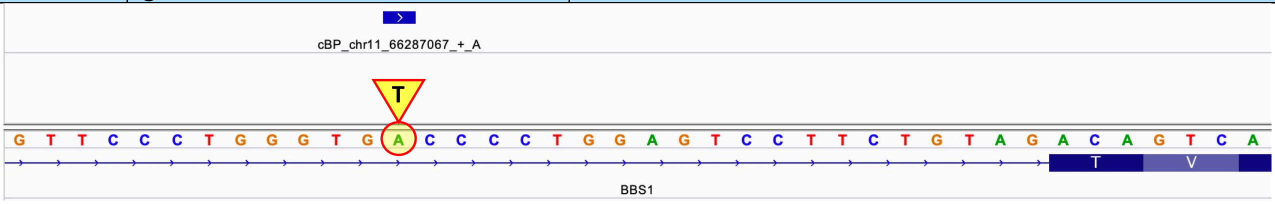

BTK	g.100609705T>C	IVS15-23A>G

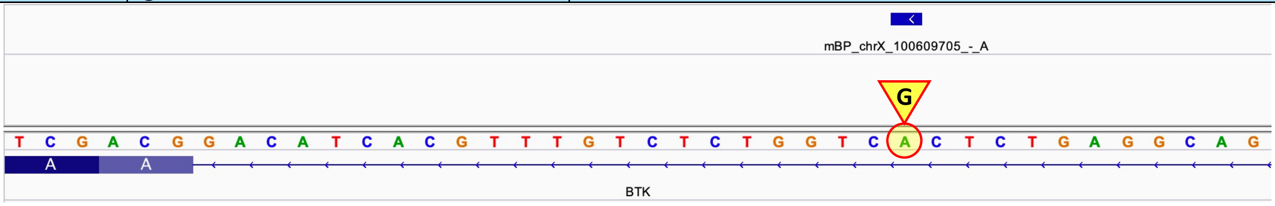

C21orf2	g.45750232T>A	IVS6-23A>T

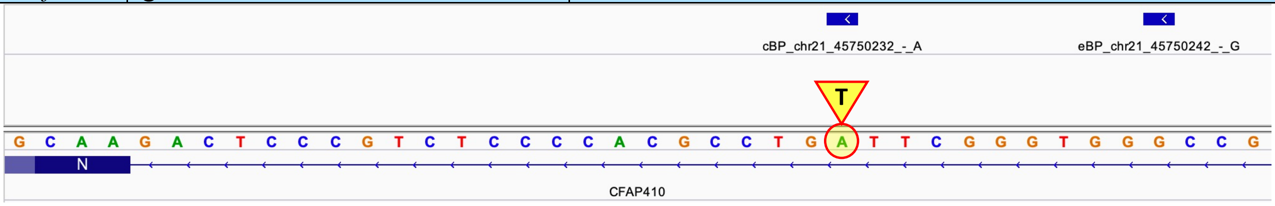

CAPN3	g.42684808del	IVS6-29delT

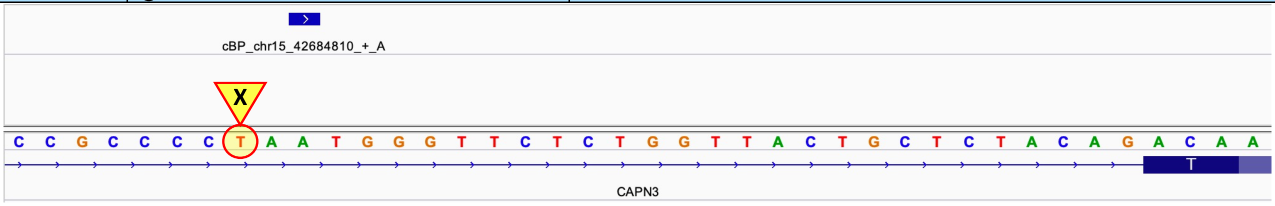

CD40LG	g.135736500_135736507del	IVS2-32_-25delAAAATGAC

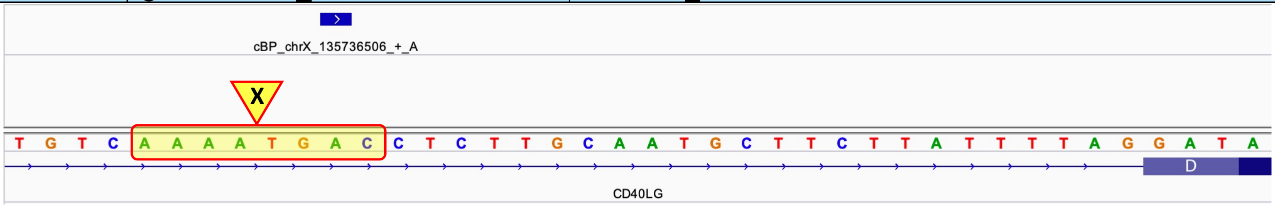

CDT1	g.88873665A>G	IVS8-24A>G

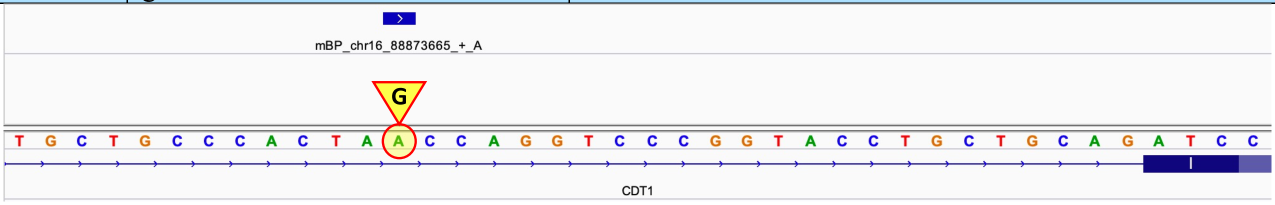

COL4A5	g.107849932A>G	IVS28-40A>G

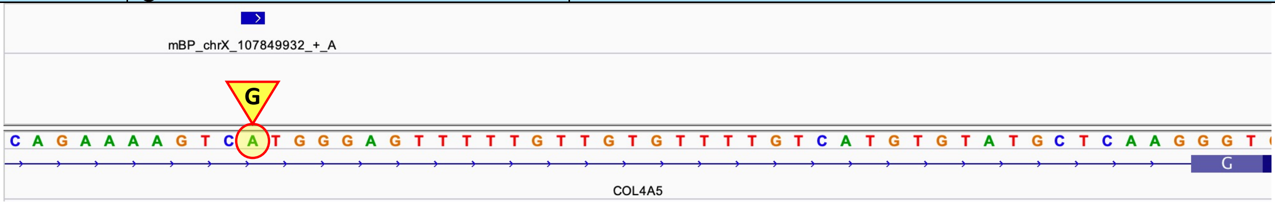

COL5A1	g.137686903T>G	IVS32-25T>G

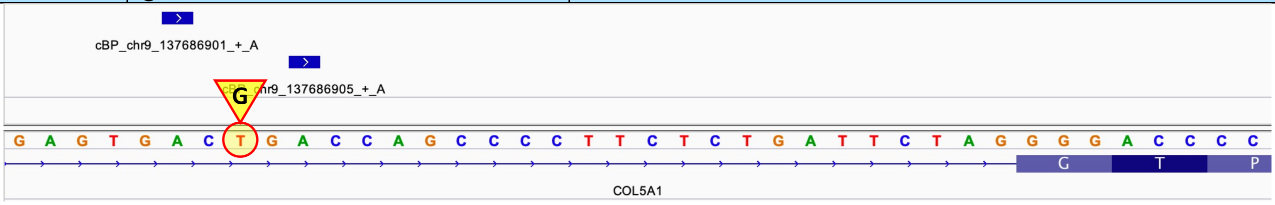

COL7A1	g.48616971T>C	IVS58-23A>G

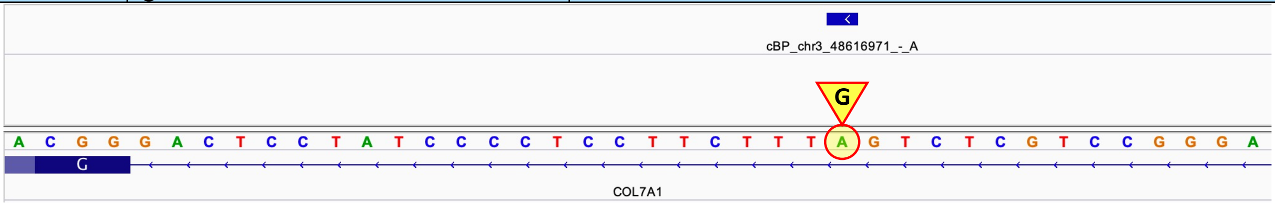

CPS1	g.211452758A>G	IVS6-24A>G

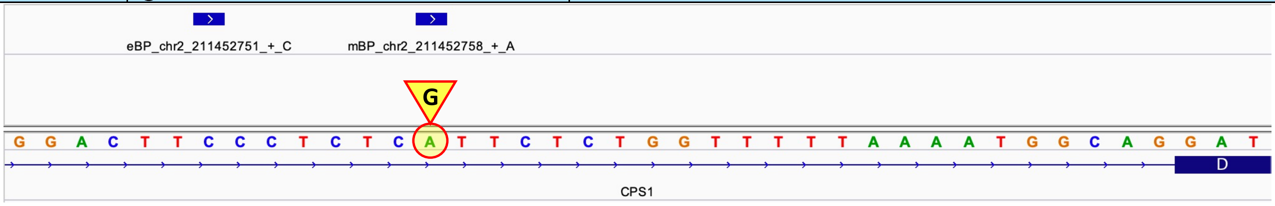

DYSF	g.71817308A>G	IVS31-33A>G

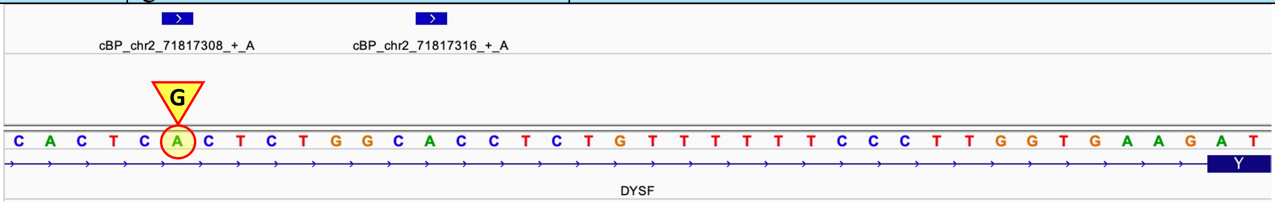

ENG	g.130578354A>G	IVS12-22T>C

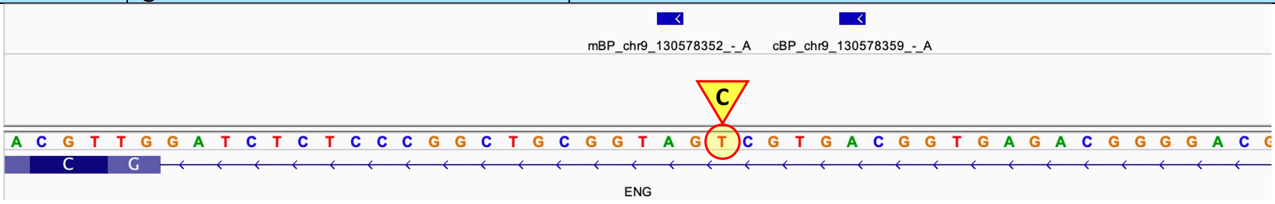

F8	g.154130469T>C	IVS18-27A>G

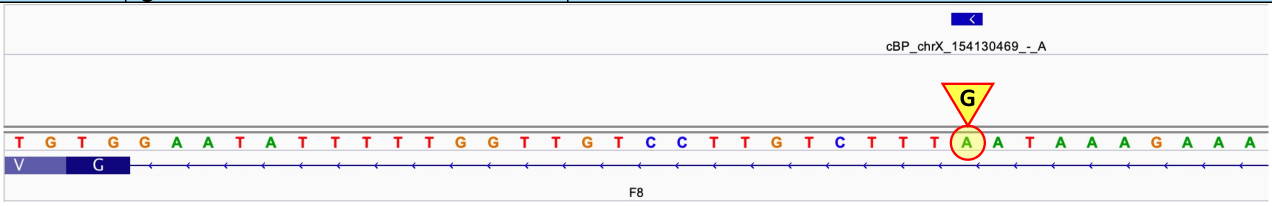

F9	g.138619496A>G	IVS2-25A>G

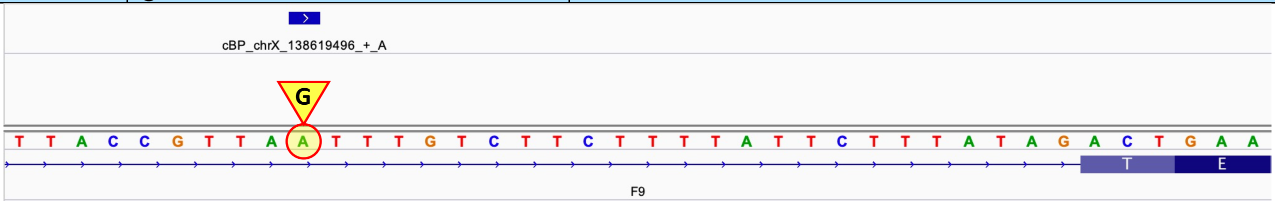

FBN2	g.127670562A>C	IVS30-26T>G

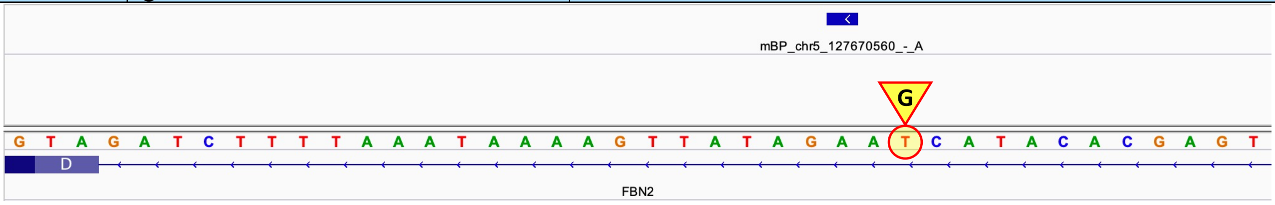

FGD1	g.54476769del	IVS12-35delA

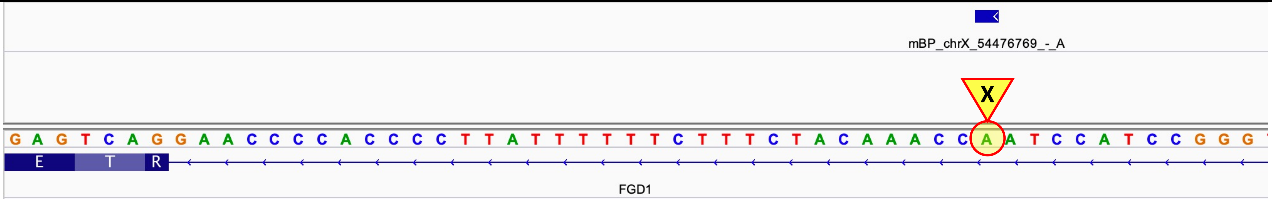

IKBKG	g.153788599A>T	IVS4-23A>T

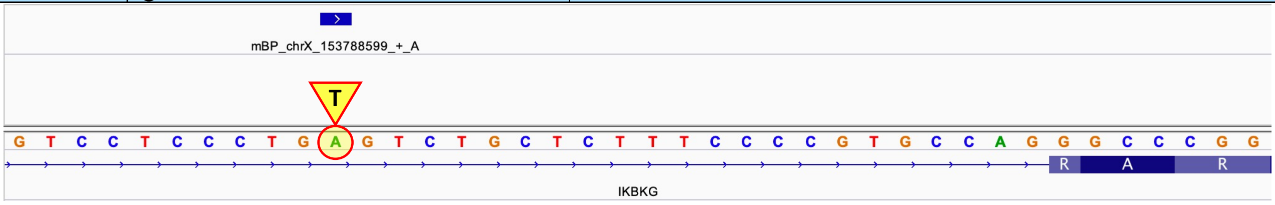

ITGB4	g.73732344T>A	IVS14-25T>A

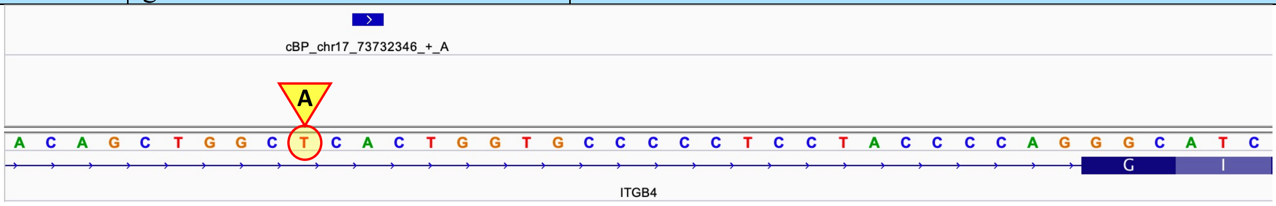

ITGB4	g.73748508T>A	IVS31-19T>A

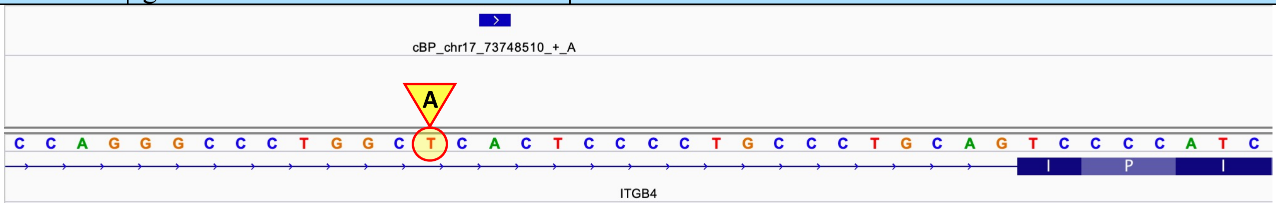

KCNH2	g.150646165T>C	IVS9-28A>G

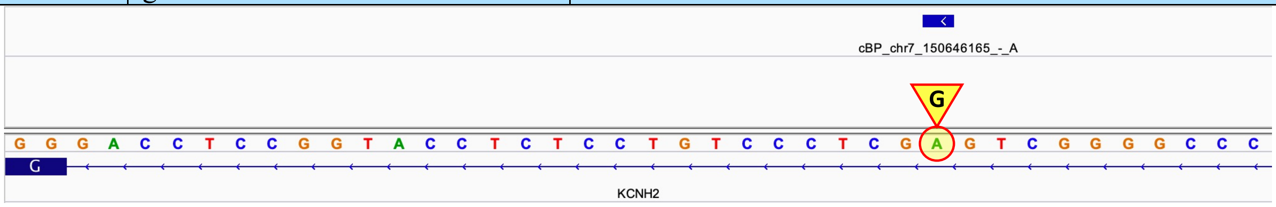

L1CAM	g.153131293T>G	IVS18-19A>C

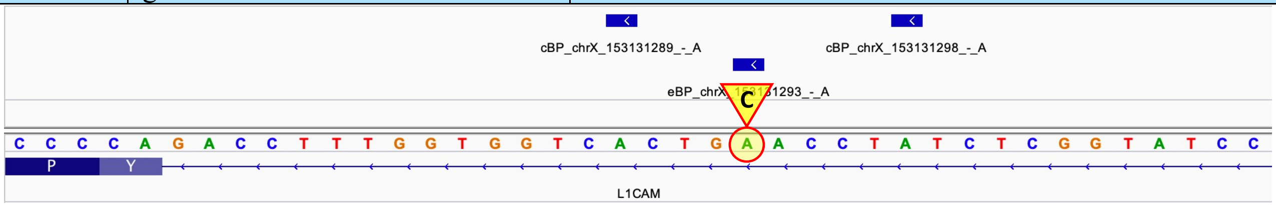

LCAT	g.67976512A>G	IVS4-22T>C

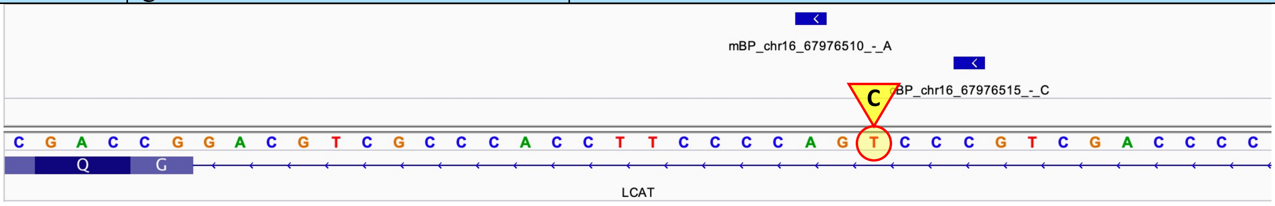

LMX1B	g.129377625_129377641del	IVS1-37_-21delGGCGCTGACGGCCGGGC

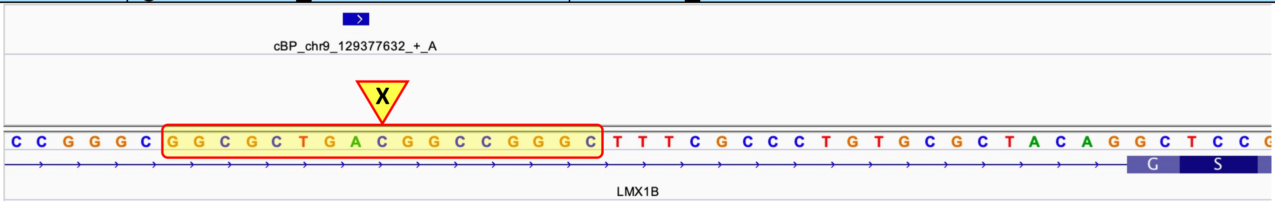

MLH1	g.37090369T>G	IVS16-26T>G

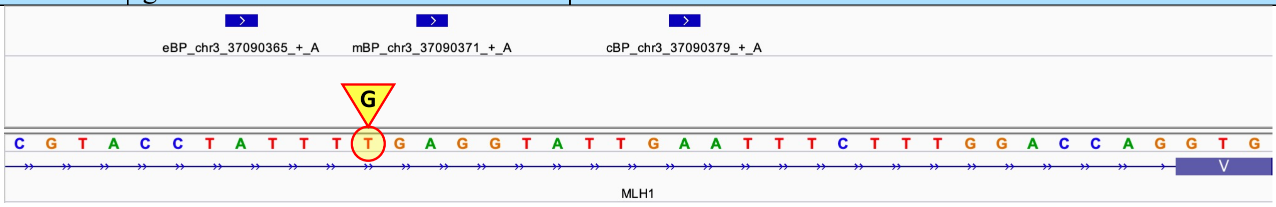

MLH1	g.37090371A>G	IVS16-24A>G

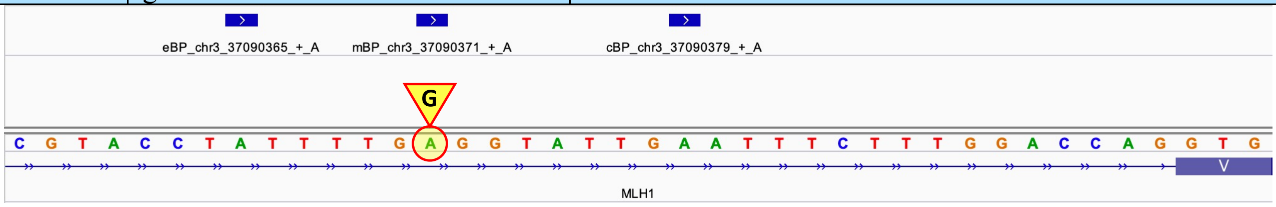

MSH2	g.47709894A>G	IVS15-24A>G

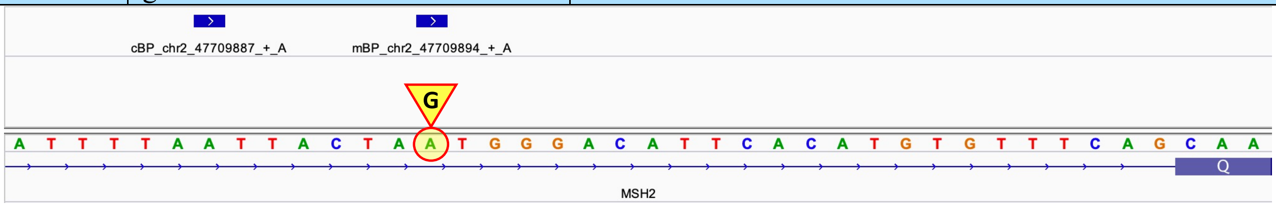

MSH6	g.48032731T>G	IVS4-26T>G

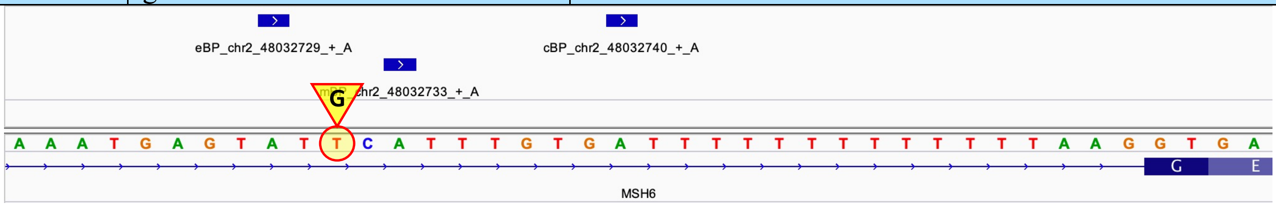

NPC1	g.21137182T>C	IVS6-28A>G

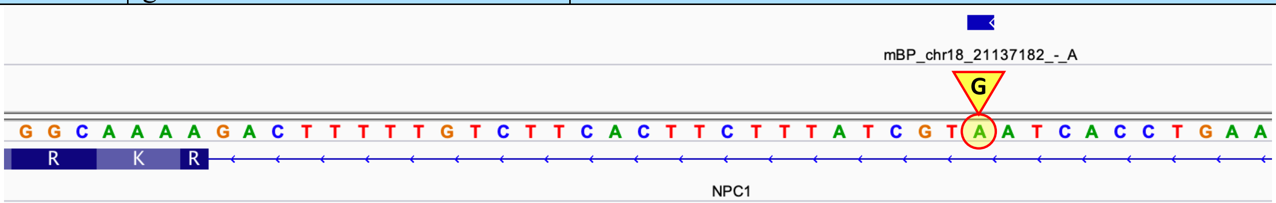

NTRK1	g.156843392T>A	IVS7-33T>A

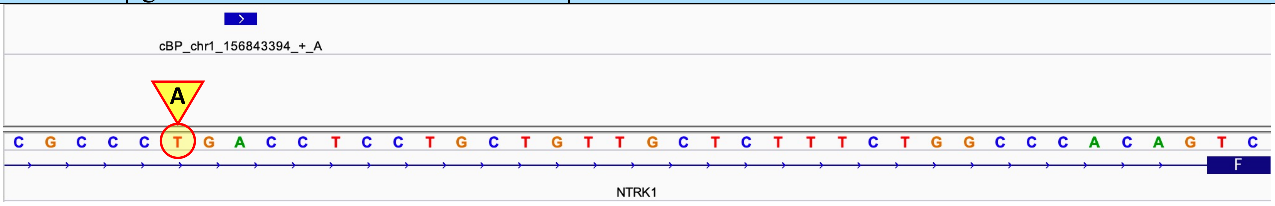

RB1	g.49039315A>T	IVS22-26A>T

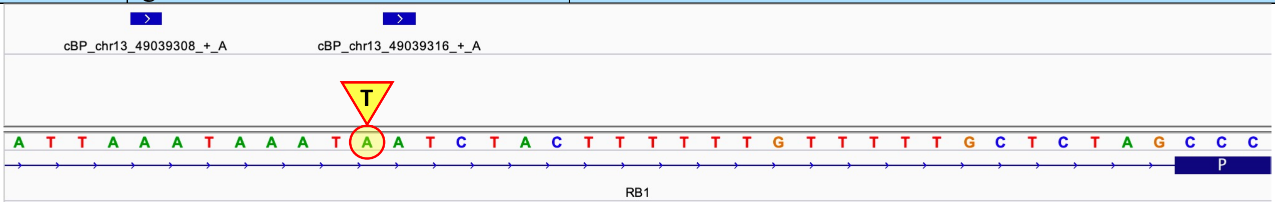

SLC5A2	g.31499327_31499349del	IVS7-31_-10delGCAAGCGGGCAGCTGAACGCCC

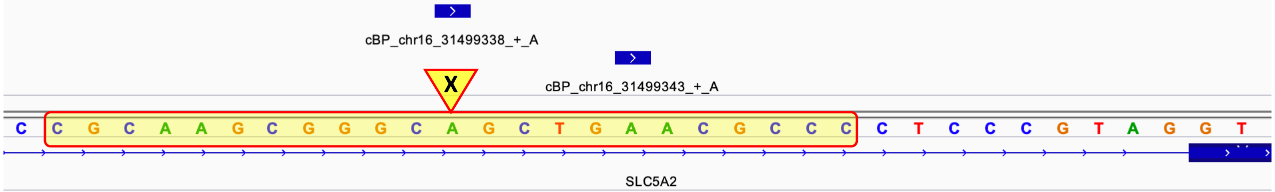

TH	g.2187017A>T	IVS11-24T>A

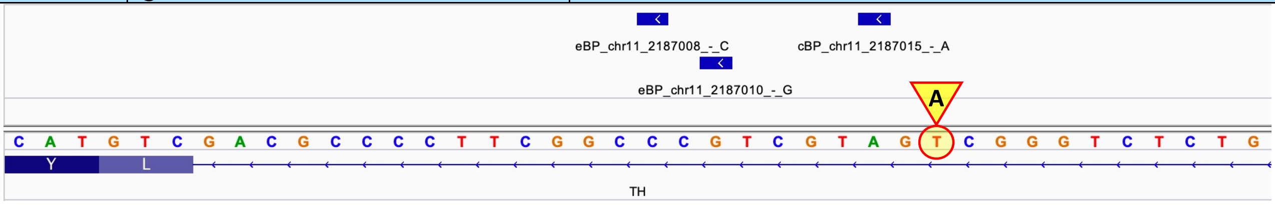

TSC2	g.2138031A>G	IVS38-18A>G

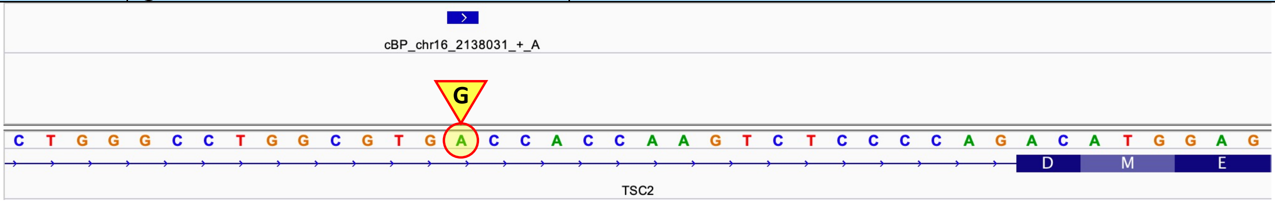

UROS	g.127477605A>C	IVS9-31T>G

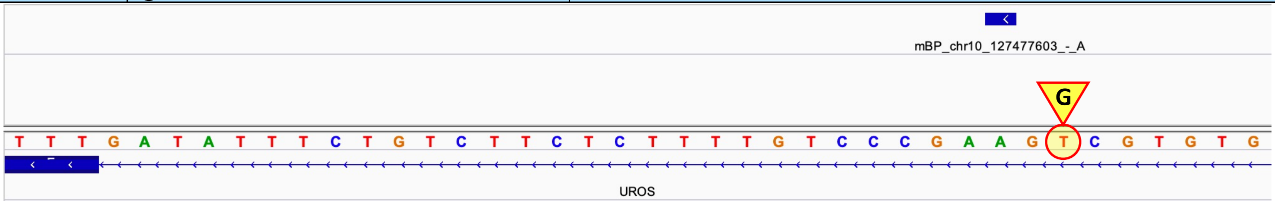

VMA21	g.150572076A>C, A>T	IVS1-27A>C, A>T

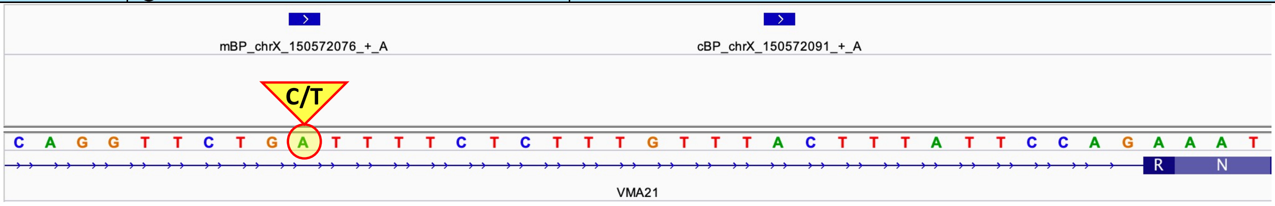

VWF	g.6101204T>A	IVS37-20A>T

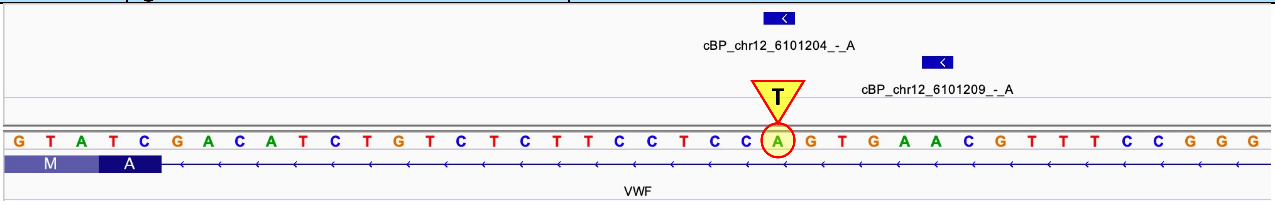

XPC	g.14209904T>C	IVS3-24A>G

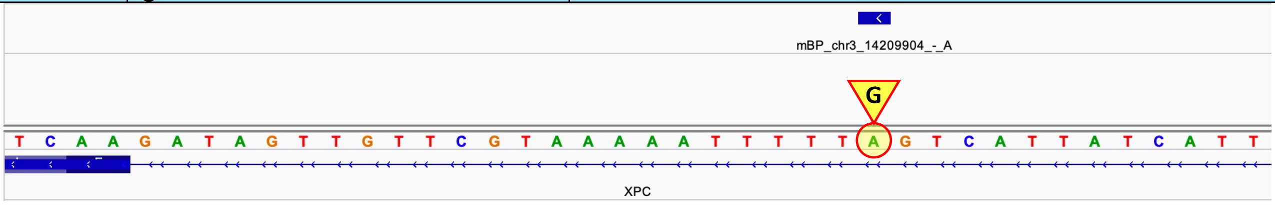

Figure S18: Schematic of the wild-type (wt) and mutant (mt) sequences of the eight pathogenic "BP" variants un-detected by BPHunter. BP positions, variant sites, and exon regions are colored in red, yellow, and blue, respectively. The value in the box is the MaxEntScan 3' splice site strengths for the constitutional AGs and the newly created AGs.

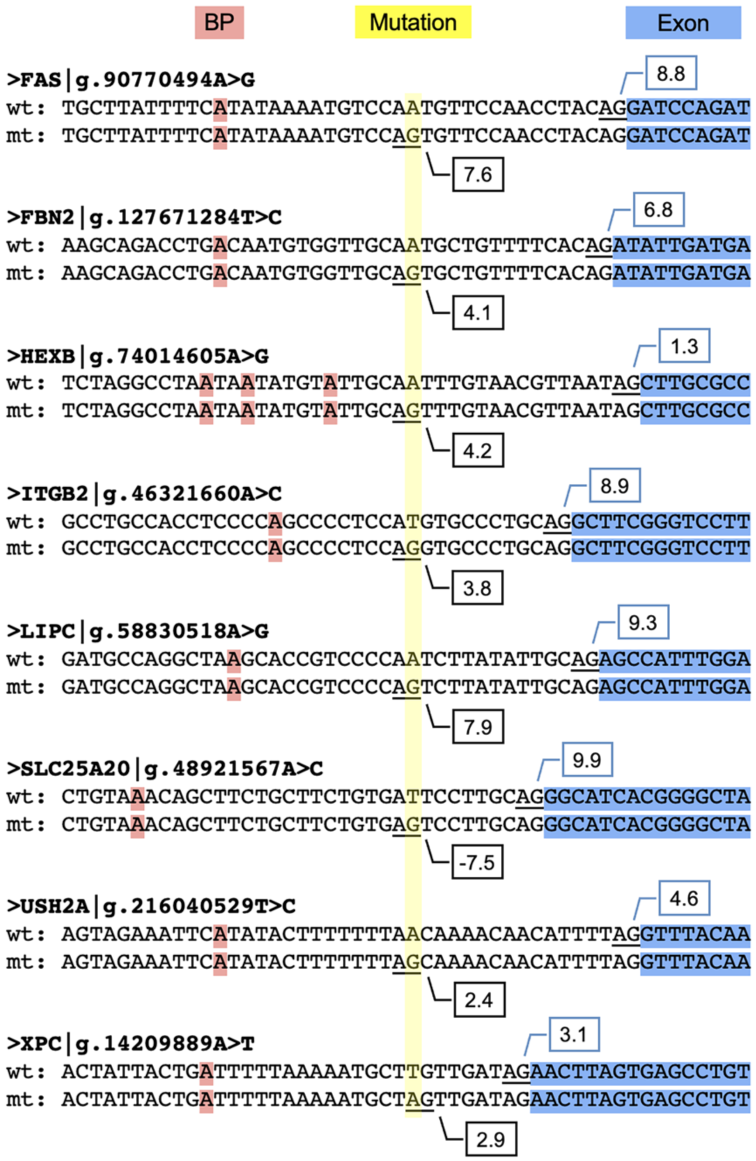

Figure S19: Comparison of pathogenic BP SNVs *vs.* common BP SNVs in the population. (a) 33 BP sites containing pathogenic variants against 659 BP sites containing common variants. (b) 22 pathogenic variants versus 386 common variants at BP positions. (c) 11 pathogenic variants versus 273 common variants at BP-2 positions.

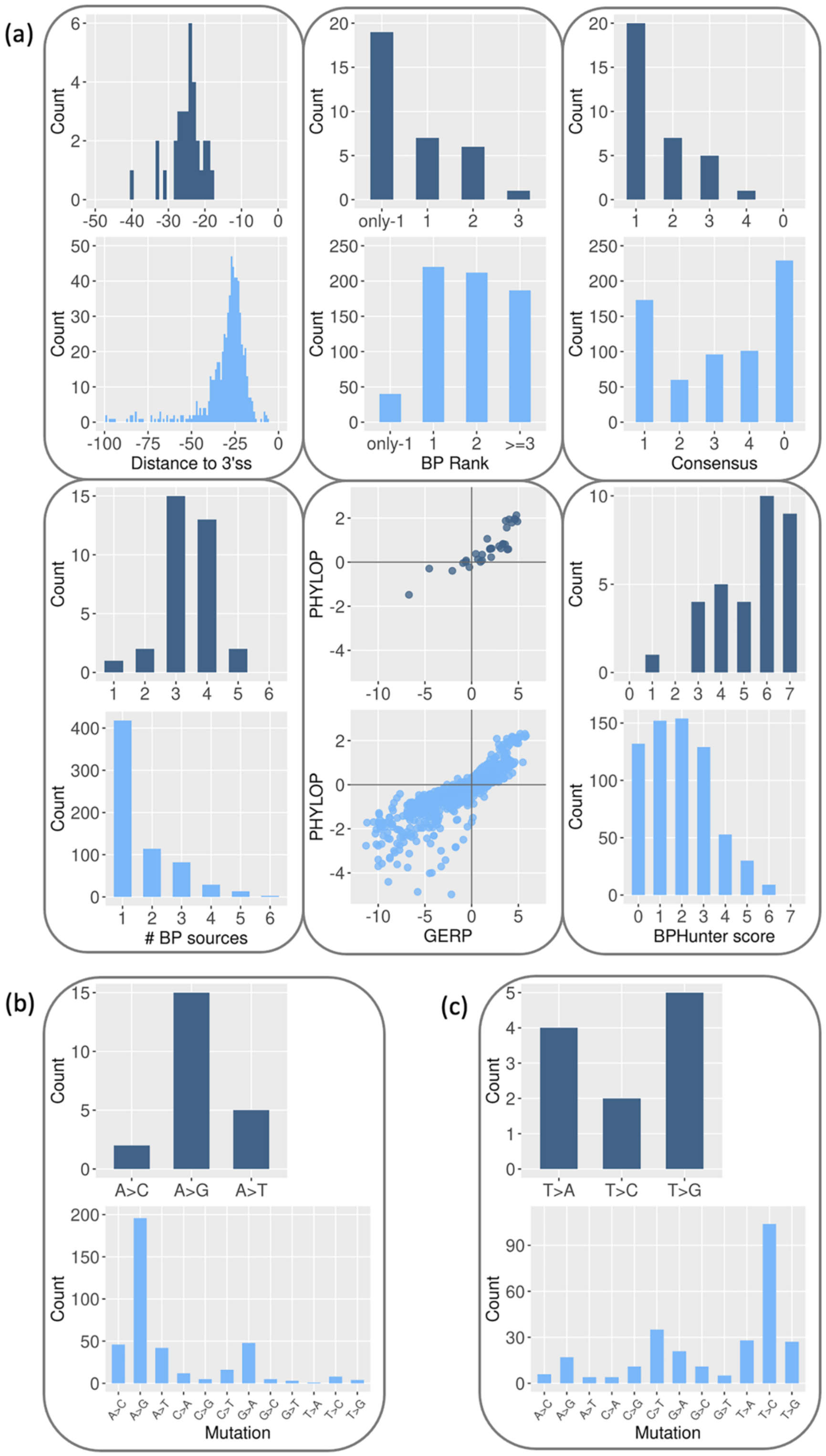

Figure S20: BPHunter scoring scheme.

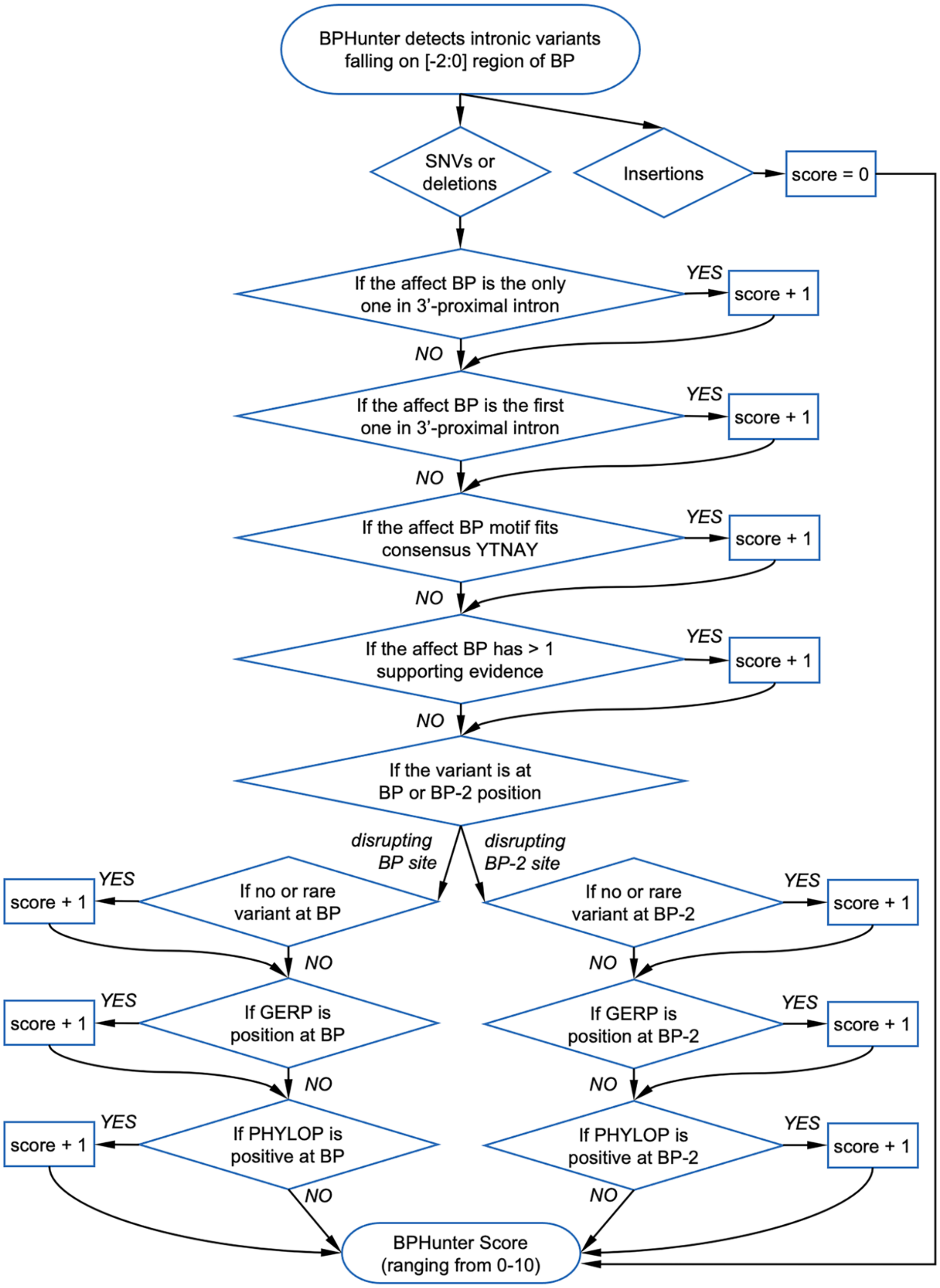

Figure S21: Demonstration of 11 BP variants with their evidence of mis-splicing from RNA-seq data. Retained intronic reads are shown as IGV alignment or Sashimi plots. Exon skipping events are shown in Sashimi plots. The green crosses in the Sashimi plots indicate the locations of BP variants.

<table>
<tr><th>Variant</th><th>Sample</th><th>Gene</th><th>Chrom</th><th>Position</th><th>Ref</th><th>Alt</th><th>Variant type</th><th>Score</th></tr>
<tr><td>Var #1</td><td>S3</td><td>IPO7</td><td>chr11</td><td>9459592</td><td>GTTTTTTTTTTTTTTT</td><td>G</td><td>del-15nt</td><td>3</td></tr>
<tr><td colspan="9">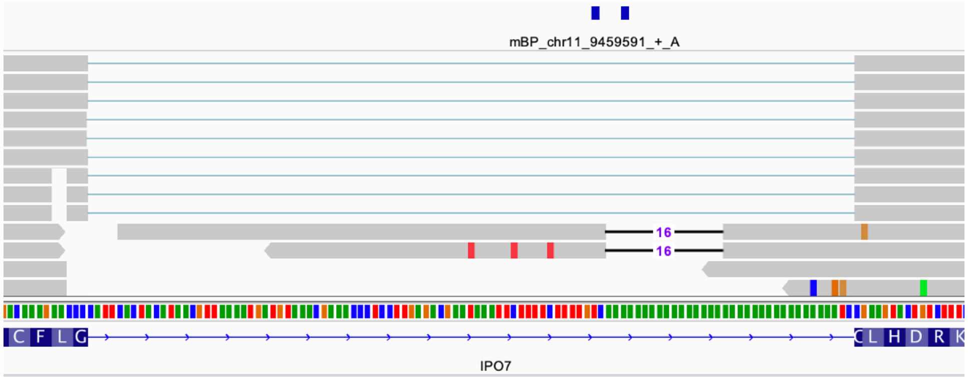
</td></tr>
<tr><td colspan="9">The intronic reads harboring the deletion were retained.</td></tr>
<tr><td>Var #2</td><td>S3</td><td>LOXL1</td><td>chr15</td><td>74238736</td><td>A</td><td>C</td><td>snv</td><td>4</td></tr>
<tr><td colspan="9">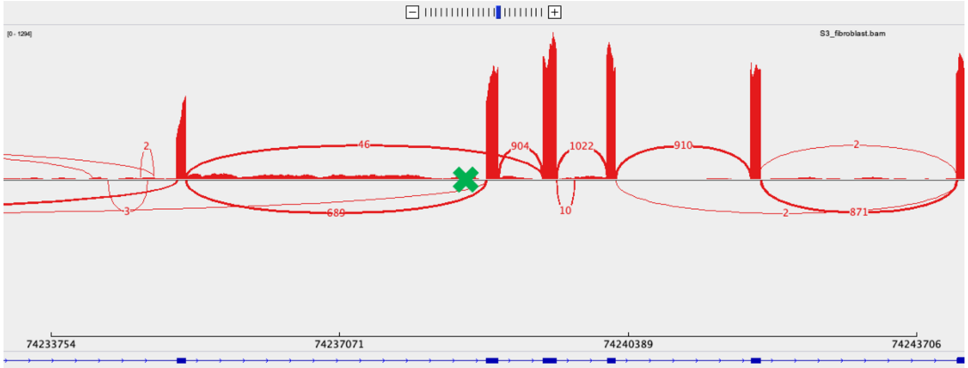
</td></tr>
<tr><td colspan="9">The exon skipping was seen in 46 aberrantly spliced junction reads.</td></tr>
<tr><td>Var #3</td><td>S4</td><td>ASAP1</td><td>chr8</td><td>131138362</td><td>A</td><td>G</td><td>snv</td><td>5</td></tr>
<tr><td colspan="9">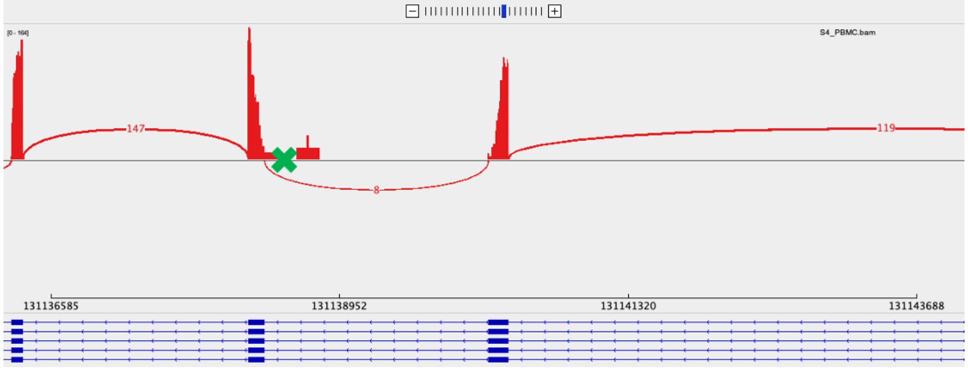
</td></tr>
<tr><td colspan="9">Splicing efficiency was significantly reduced, as the splice junction reads (8) were much lower than the adjacent exons (147 and 119).</td></tr>
</table>

Var #4	S4	*TP53BP2*	chr1	223976890	T	A	snv	3

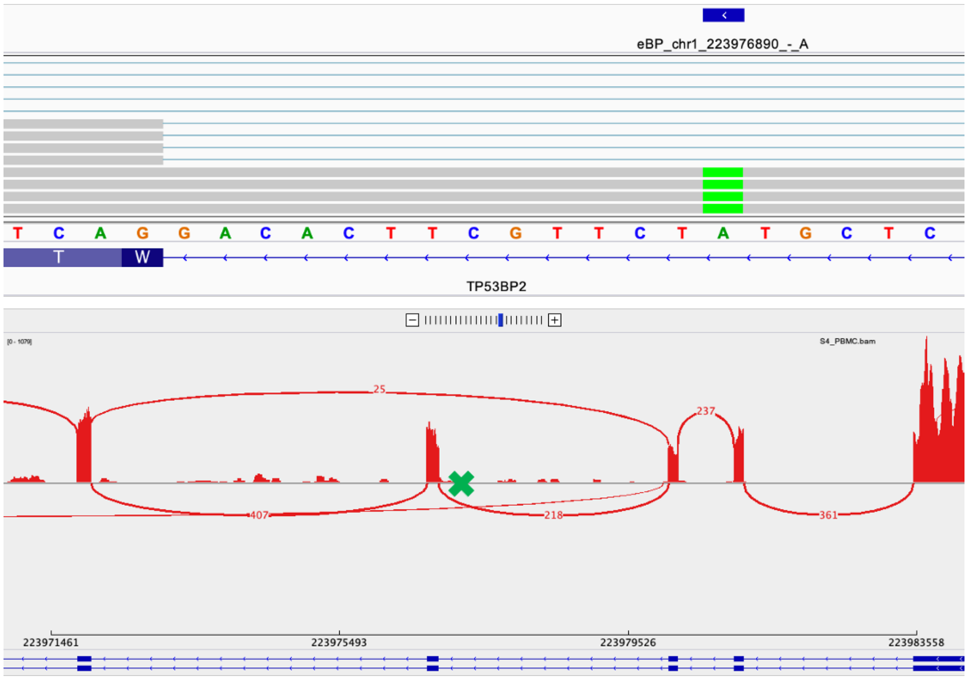

The intronic reads harboring the variant were retained. The exon skipping was seen in 25 aberrantly spliced junction reads.

Var #5	S8	*TPP1*	chr11	6637323	T	C	snv	3

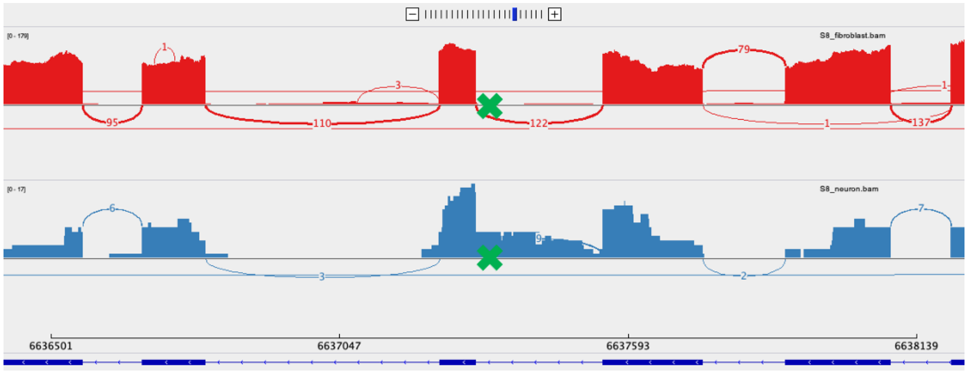

Intronic retention in neurons but not in fibroblasts.

Var #6	S9	*BRD8*	chr5	137503787	GT	G	del-1nt	6

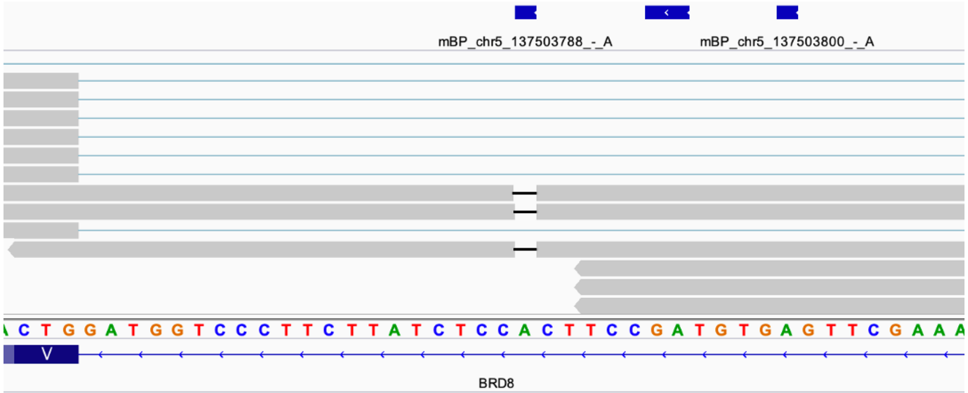

The intronic reads harboring the deletion were retained.

Var #7	S10	*SREBF1*	chr17	17719367	CA	C	del-1nt	4

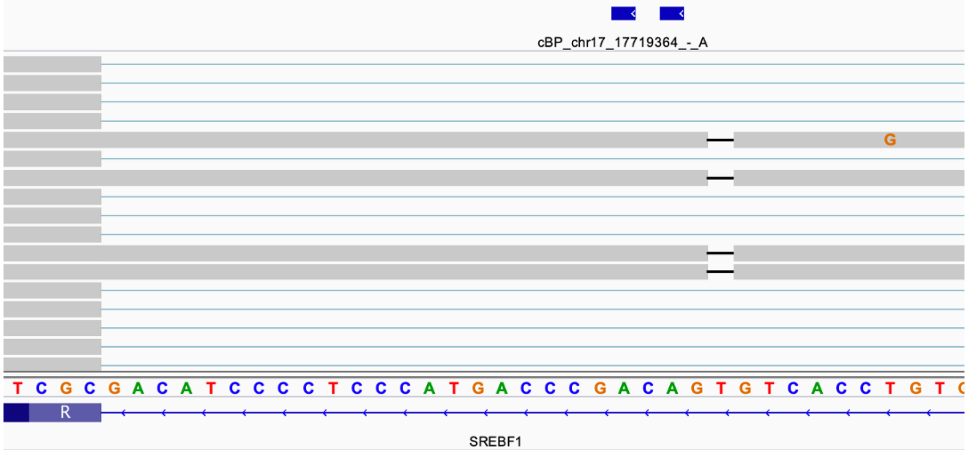

The intronic reads harboring the deletion were retained.

Var #8	S12	*MTR*	chr1	237016225	A	G	snv	5

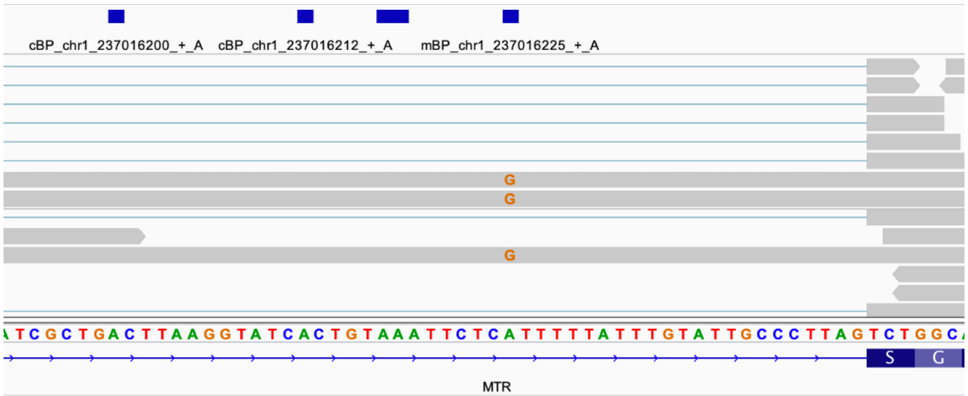

The intronic reads harboring the variant were retained.

Var #9	S12	*WASL*	chr7	123324655	T	C	snv	3

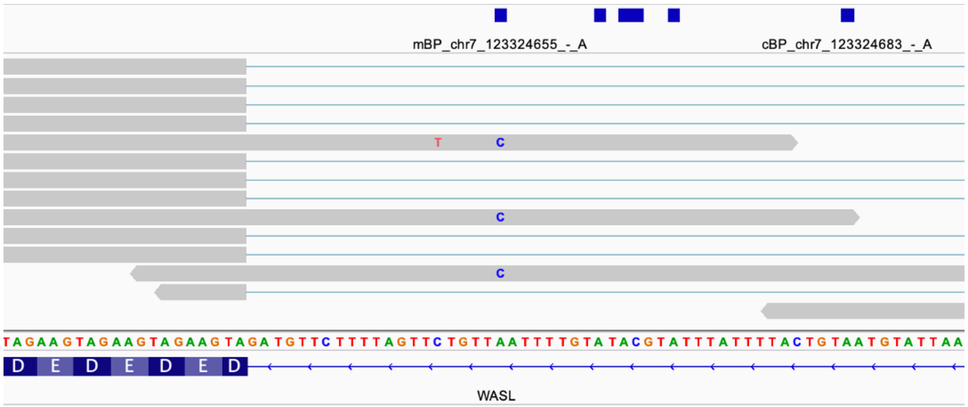

The intronic reads harboring the variant were retained.

Var #10	S14	*CDK12*	chr17	37682091	A	G	snv	5

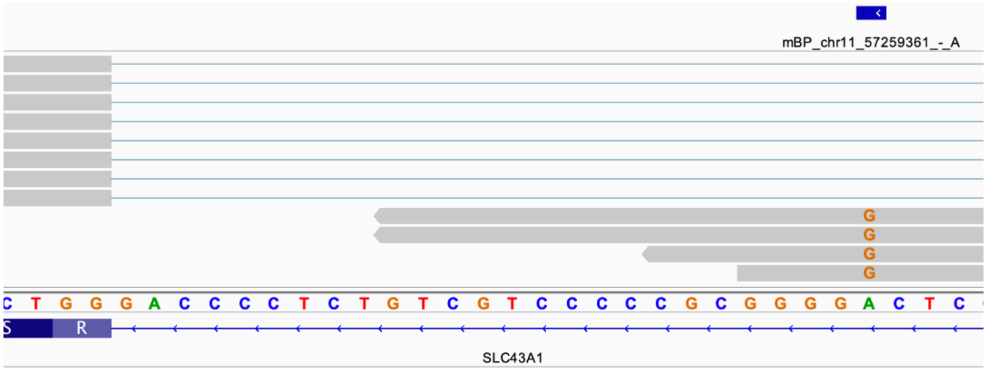

The intronic reads harboring the variant were retained.

Var #11	S14	*SLC43A1*	chr11	57259361	T	G	snv	5

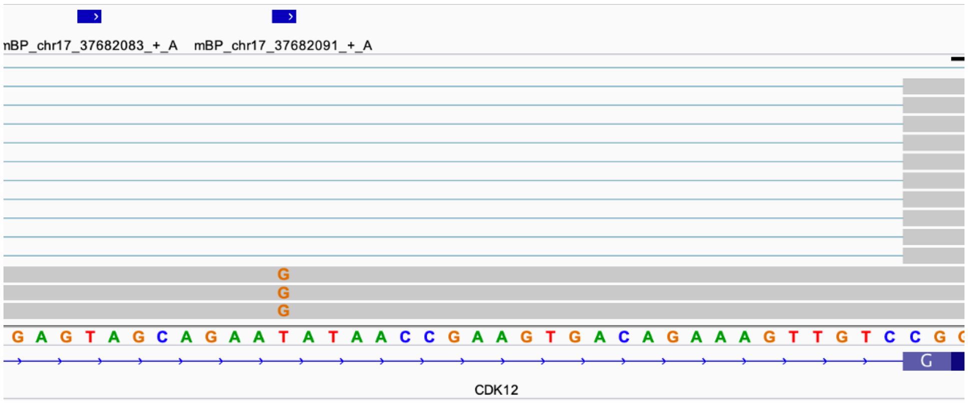

The intronic reads harboring the variant were retained.

SUPPLEMENTAL TABLES

Table S1: Identification of BP positions (eBP_BPHunter) from 15 RNA-seq datasets from three *DBR1*-mutated patients.

RNA-seq dataset	Genome alignment		BLAST to 20-nt 5'ss library		BLAST to 200-nt 3'ss library	
	**# Total reads**	**% Unmapped**	**# Reads**	**# 5'ss**	**# Reads**	**# 3'ss**
DBR1_P1_NS	67,898,775	32.2%	474,471	27,470	17,650	856
DBR1_P1_IFNa	67,476,272	34.1%	515,604	29,188	22,864	753
DBR1_P1_pIC	69,451,012	32.8%	521,275	29,155	18,002	949
DBR1_P1_HSV1_8h	65,364,204	32.0%	522,843	37,353	17,226	1,046
DBR1_P1_HSV1_24h	77,650,960	60.5%	806,010	21,031	11,322	1,531
DBR1_P5_NS	66,453,726	30.0%	457,434	29,057	19,557	796
DBR1_P5_IFNa	67,627,001	32.7%	505,975	30,559	26,947	915
DBR1_P5_pIC	71,708,411	30.7%	486,120	28,933	22,720	779
DBR1_P5_HSV1_8h	69,381,682	29.9%	461,178	29,749	26,033	776
DBR1_P5_HSV1_24h	72,165,677	58.1%	789,468	22,596	11,675	1,399
DBR1_P6_NS	74,418,160	27.4%	459,476	30,903	20,115	825
DBR1_P6_IFNa	71,982,293	28.3%	416,590	26,891	20,972	803
DBR1_P6_pIC	74,693,142	32.0%	415,654	26,067	16,232	721
DBR1_P6_HSV1_8h	68,593,257	30.4%	445,085	30,341	19,072	806
DBR1_P6_HSV1_24h	74,216,041	58.6%	687,534	24,793	10,512	1,333
				**# 5'ss-BP junction reads**	280,899	
				**# BP positions**	8,682	

Table S2: Matching the BP consensus sequence YTNAY to BP positions, and sliding the matches within a window of [-2, +2] of BP positions, for consensus-guided positional adjustment of BP positions. The percentages in the table refer to the proportions at specific positions that matched the consensus sequence.

	**-2**	**-1**	**BP**	**+1**	**+2**
eBP_Mercer	1.2%	0.7%	28.5%	4.4%	2.9%
eBP_Taggart	1.5%	2.8%	35.6%	0.5%	0.1%
eBP_Pineda	1.0%	0.3%	24.0%	2.2%	0.5%
eBP_Talhouarne	0.4%	0.0%	2.1%	1.7%	0.0%
eBP_Briese	0.8%	0.0%	39.1%	1.6%	0.0%
eBP_BPHunter	1.0%	0.6%	19.3%	5.6%	4.9%
**eBP**	1.2%	0.8%	23.5%	2.7%	1.2%
cBP_BPP	0.0%	0.0%	43.5%	0.1%	0.0%
cBP_Branchpointer	0.3%	0.0%	39.5%	5.5%	0.5%
cBP_LaBranchoR	0.3%	0.0%	44.4%	1.2%	0.0%
cBP_BPHunter	0.0%	0.0%	99.7%	0.0%	0.0%
**cBP**	0.3%	0.0%	35.6%	4.2%	0.4%

Table S3: The overlaps and exclusive BP positions across the entire collection of ten BP datasets.

	eBP_Mercer	eBP_Taggart	eBP_Pineda	eBP_Talhouarne	eBP_Briese	eBP_BPHunter	cBP_BPP	cBP_Branchpointer	cBP_LaBranchoR	cBP_BPHunter	Exclusive
eBP_Mercer	-										9,794
eBP_Taggart	12,544	-									7,857
eBP_Pineda	27,716	13,114	-								60,907
eBP_Talhouarne	135	63	41	-							73
eBP_Briese	11,615	5,730	16,239	3	-						4,677
eBP_BPHunter	3,511	1,371	2,450	103	1,025	-					2,996
cBP_BPP	22,251	12,240	39,600	12	24,816	2,275	-				66,513
cBP_Branchpointer	31,580	14,758	52,798	36	32,328	2,992	143,512	-			114,144
cBP_LaBranchoR	28,869	14,080	49,435	63	31,107	3,035	117,669	155,044	-		24,742
cBP_BPHunter	2,795	2,056	5,037	4	3,328	307	13,788	15,087	14,347	-	3,791

Table S4: Characterization of BP in major and minor introns: nucleotide frequencies of BP motifs [-9, +3], 3'ss motifs [-10, +3] and 5'ss motifs [-3, +10].

**Major intron**	**BP**		-9	-8	-7	-6	-5	-4	-3	**-2**	-1	**0**	+1	+2	+3
		A:	24.4%	24.9%	25.0%	26.0%	25.1%	28.3%	15.5%	6.9%	25.2%	**91.8%**	19.3%	22.7%	21.2%
		C:	24.8%	24.1%	22.7%	20.6%	20.8%	22.0%	37.1%	12.3%	29.0%	**3.8%**	33.1%	27.4%	24.4%
		G:	20.3%	20.4%	21.6%	21.9%	21.5%	19.5%	15.5%	8.9%	25.6%	**2.5%**	14.9%	16.3%	18.6%
		T:	30.5%	30.6%	30.6%	31.6%	32.6%	30.1%	32.0%	72.0%	20.2%	**1.9%**	32.7%	33.6%	35.7%
	**3'ss**		-10	-9	-8	-7	-6	-5	-4	-3	**-2**	**-1**	+1	+2	+3
		A:	8.6%	9.8%	10.7%	11.3%	8.6%	9.1%	23.8%	5.8%	**100%**	**0.0%**	25.8%	24.9%	25.8%
		C:	28.2%	29.4%	32.3%	33.3%	34.0%	29.3%	27.1%	64.7%	**0.0%**	**0.0%**	14.3%	19.0%	23.3%
		G:	10.7%	11.3%	10.3%	9.0%	6.3%	6.3%	20.8%	0.3%	**0.0%**	**100%**	48.7%	19.3%	23.6%
		T:	52.5%	49.5%	46.8%	46.4%	51.1%	55.3%	28.3%	29.1%	**0.0%**	**0.0%**	11.2%	36.8%	27.3%
	**5'ss**		-3	-2	-1	**+1**	**+2**	+3	+4	+5	+6	+7	+8	+9	+10
		A:	33.0%	64.0%	9.5%	**0.0%**	**0.0%**	59.8%	69.4%	8.7%	17.5%	29.6%	22.5%	22.0%	22.4%
		C:	36.3%	10.8%	2.7%	**0.0%**	**1.0%**	2.9%	7.7%	5.5%	14.9%	19.1%	25.3%	26.6%	24.0%
		G:	18.7%	11.5%	81.2%	**100%**	**0.0%**	34.3%	12.0%	78.2%	19.2%	29.9%	23.8%	24.4%	25.8%
		T:	12.0%	13.7%	6.5%	**0.0%**	**99.0%**	3.0%	11.0%	7.6%	48.3%	21.5%	28.4%	27.0%	27.7%
**Minor intron**	**BP**		-9	-8	-7	-6	-5	-4	-3	**-2**	-1	**0**	+1	+2	+3
		A:	22.1%	21.6%	20.5%	19.1%	18.2%	18.9%	11.5%	**3.5%**	32.8%	**94.8%**	15.5%	22.6%	21.9%
		C:	20.6%	20.3%	19.1%	17.2%	35.0%	43.0%	23.5%	**11.2%**	21.0%	**3.0%**	39.7%	26.1%	23.6%
		G:	21.6%	19.2%	18.9%	17.3%	16.1%	13.9%	13.3%	**7.2%**	24.3%	**0.8%**	17.3%	14.9%	19.0%
		T:	35.7%	38.9%	41.4%	46.4%	30.7%	24.2%	51.7%	**78.1%**	21.9%	**1.3%**	27.5%	36.5%	35.5%
	**3'ss**		-10	-9	-8	-7	-6	-5	-4	-3	**-2**	**-1**	+1	+2	+3
		A:	25.4%	21.0%	20.7%	24.5%	19.2%	16.2%	16.8%	9.6%	**99.2%**	**0.5%**	45.6%	10.5%	41.4%
		C:	30.6%	27.2%	27.8%	25.5%	30.3%	35.0%	31.1%	64.7%	**0.0%**	**24.0%**	20.6%	21.2%	16.4%
		G:	12.9%	18.5%	17.3%	18.2%	18.0%	11.1%	16.1%	0.9%	**0.8%**	**73.7%**	15.8%	13.2%	16.8%
		T:	31.1%	33.3%	34.2%	31.8%	32.4%	37.7%	36.0%	24.8%	**0.0%**	**1.8%**	18.0%	55.1%	25.4%
	**5'ss**		-3	-2	-1	**+1**	**+2**	+3	+4	+5	+6	+7	+8	+9	+10
		A:	30.3%	24.8%	27.2%	**27.3%**	**0.0%**	100%	0.2%	0.0%	0.3%	2.4%	2.6%	6.5%	18.6%
		C:	23.0%	34.5%	20.0%	**0.0%**	**0.2%**	0.0%	0.5%	99.6%	96.4%	1.1%	7.1%	21.6%	27.0%
		G:	24.9%	15.3%	12.6%	**72.7%**	**0.0%**	0.0%	0.0%	0.2%	0.0%	1.1%	5.4%	9.8%	20.0%
		T:	21.8%	25.4%	40.2%	**0.0%**	**99.9%**	0.0%	99.4%	0.3%	3.3%	95.5%	85.0%	62.2%	34.4%

Table S5: Characterization of BP in major and minor introns: distance from BP to 3'ss, and BP-snRNA binding energy.

	**Major Intron**	**Minor Intron**
# BP	384,514	1,725
# Introns	199,393	666
**BP-to-3'ss distance**		
Min	-4	-4
25th	-23	-12
Med	-27	-15
Avg	-29	-25
75th	-32	-32
Max	-99	-97
**BP-to-3'ss distance in ranges**		
[3–10]	0.79%	4.27%
[11–15]	1.45%	22.59%
[16–20]	10.04%	10.80%
[21–25]	29.92%	15.77%
[26–30]	27.34%	17.22%
[31–35]	14.09%	11.50%
[36–40]	6.28%	5.66%
[41–45]	3.17%	3.06%
[46–50]	1.78%	2.37%
[51–100]	5.14%	6.76%
**BP-snRNA binding energy**		
Min	0	0
25th	0	-0.9
Med	-0.7	-2.8
Avg	-1.3	-3.7
75th	-2.2	-6.2
Max	-10.2	-10.2

Table S6: Positional comparison of human population variants between BP region [-3, +1], 5’ss region [-1, +3], 3’ss region [-3, +1], and random intronic and exonic backgrounds.

**Position category**	**Total positions**	**% hit in population variants**	**Median of log10(MAF)**	**% singleton population variants** (AC = 1)	**% rare population variants** (MAF < 1%)	**% common population variants** (MAF ≥ 1%)
5SS-1	185,945	15.10%	-5.155	57.33%	41.75%	0.92%
5SS+1	185,945	10.68%	-5.156	65.53%	33.99%	0.48%
5SS+2	185,945	7.40%	-5.156	63.22%	35.94%	0.84%
5SS+3	185,945	14.21%	-5.156	56.91%	41.74%	1.35%
BP-3	386,209	17.14%	-5.154	53.31%	44.80%	1.89%
BP-2	386,209	12.74%	-5.155	54.59%	43.61%	1.80%
BP-1	386,209	15.40%	-5.155	54.36%	43.80%	1.84%
BP	386,209	14.54%	-5.153	54.14%	43.91%	1.95%
BP+1	386,209	18.71%	-5.069	51.43%	46.53%	2.04%
3SS-3	185,850	13.85%	-5.156	57.42%	41.30%	1.27%
3SS-2	185,850	6.38%	-5.156	66.23%	33.24%	0.53%
3SS-1	185,850	10.42%	-5.156	64.34%	35.20%	0.45%
3SS+1	185,850	15.14%	-5.155	56.97%	42.18%	0.86%
INTRON	1,000,000	17.43%	-5.023	51.04%	46.77%	2.19%
EXON	1,000,000	16.00%	-5.149	52.20%	46.41%	1.39%

Table S7: Positional comparison of cross-species conservation scores between BP region [-3, +1], 5’ss region [-1, +3], 3’ss region [-3, +1], and random intronic and exonic backgrounds.

POS	Total	GERP				PhyloP			
		Median	75th	90th	% >1	Median	75th	90th	% >1
5SS-1	185,945	4.63	5.39	5.74	86.52%	2.12	2.56	2.75	72.36%
5SS+1	185,945	5.08	5.54	5.82	94.36%	2.51	2.69	2.81	90.10%
5SS+2	185,945	5.05	5.53	5.81	93.36%	2.03	2.17	2.26	84.49%
5SS+3	185,945	2.76	4.22	5.16	72.88%	0.71	1.56	2.27	38.58%
BP-3	386,209	0.51	2.31	3.67	43.13%	0.10	0.62	1.34	14.81%
BP-2	386,209	1.02	2.89	4.24	50.21%	0.20	0.83	1.90	18.53%
BP-1	386,209	0.37	2.17	3.58	41.02%	0.08	0.57	1.26	13.58%
BP	386,209	0.42	2.57	4.09	43.23%	0.08	0.72	1.83	16.13%
BP+1	386,209	0.36	2.18	3.61	40.98%	0.07	0.55	1.22	13.08%
3SS-3	185,850	2.79	4.25	5.20	72.96%	0.72	1.59	2.28	39.13%
3SS-2	185,850	5.07	5.54	5.82	93.49%	2.03	2.18	2.27	84.61%
3SS-1	185,850	5.09	5.55	5.82	94.43%	2.51	2.70	2.81	90.07%
3SS+1	185,850	4.63	5.40	5.76	86.52%	2.12	2.58	2.75	72.24%
INTRON	1,000,000	0.10	1.18	2.53	27.60%	0.05	0.40	0.91	8.64%
EXON	1,000,000	3.61	5.13	5.67	73.24%	1.11	2.20	2.61	52.61%

Table S8: The 48 reported pathogenic BP variants underlying human inherited disorders, with experimentally confirmed molecular consequences (the same as main **Table 2**, with references added).

**Pathogenic BP variants**					**BPHunter detection**	
**Gene**	**Variant**	**Dist to 3'ss**	**Disease**	**Consequence**	**Rank**	**Hit position**
ABCC8 (42)	g.17452526T>C	-20	Hyperinsulinemic hypoglycemia	partial retention of intron-11 (73nt)	#1/2	0
ALPL (43)	g.21896765_21896784del	-33	Hypophosphatasia	complete skipping of exon-8 and exon-7/8	#1/1	-2|-1|0
BBS1 (44)	g.66287067A>T	-21	Retinitis pigmentosa	complete skipping of exon-8 and exon-7/8, partial skipping of exon-8 (30nt)	#1/1	0
BTK (45)	g.100609705T>C	-23	Agammaglobulinemia	complete skipping of exon-16	#1/1	0
C21orf2 (46)	g.45750232T>A	-23	Axial spondylometaphyseal dysplasia	complete retention of intron-6	#2/3	0
CAPN3 (47)	g.42684808del	-29	Calpainopathy	partial retention of intron-6 (389nt)	#1/1	-2
CD40LG (48)	g.135736500_135736507del	-32	X-linked hyper-IgM syndrome	complete skipping of exon-3	#1/1	-2|-1|0
CDT1 (49)	g.88873665A>G	-24	Meier-Gorlin syndrome	complete retention of intron-8, complete skipping of exon-9	#1/1	0
COL4A5 (50)	g.107849932A>G	-40	Alport syndrome	complete skipping of exon-29, partial skipping of exon-29 (43nt)	#1/1	0
COL5A1 (51)	g.137686903T>G	-25	Ehlers-Danlos syndrome type II	partial skipping of exon-33 (45nt)	#1/2	-2
COL7A1 (52)	g.48616971T>C	-23	Dystrophic epidermolysis bullosa	complete retention of intron-58 and intron-58/59, complete skipping of exon-59	#1/1	0
CPS1 (53)	g.211452758A>G	-24	Hyperammonemia	complete skipping of exon-7	#1/2	0
DYSF (54)	g.71817308A>G	-33	Limb-girdle muscular dystrophy	complete retention of intron-31	#2/2	0
ENG (55)	g.130578354A>G	-22	pulmonary arterial hypertension	complete skipping of exon-13	#1/2	-2
F8 (56)	g.154130469T>C	-27	Hemophilia A	complete skipping of exon-19	#1/1	0
F9 (57)	g.138619496A>G	-25	Hemophilia B	partial retention of intron-2 (25nt)	#1/1	0
FAS (58)	g.90770494A>G	-16	Autoimmune lymphoproliferative syndrome	complete skipping of exon-6	N.D.	
FBN2 (59)	g.127670562A>C	-26	Congenital contractural arachnodactyly	complete skipping of exon-31	#1/1	-2
FBN2 (60)	g.127671284T>C	-15	Congenital contractural arachnodactyly	complete skipping of exon-29	N.D.	
FGD1 (61)	g.54476769del	-35	Aarskog–Scott syndrome	complete skipping of exon-13	#1/1	0
HEXB (62)	g.74014605A>G	-17	Sandhoff disease	partial retention of intron-10 (37nt)	N.D.	
IKBKG (63)	g.153788599A>T	-23	Ectodermal dysplasia with primary immunodeficiencies	complete skipping of exon-5 and exon-3/4/5/6, complete retention of intron-4	#1/1	0
ITGB2 (64)	g.46321660A>C	-12	Leukocyte adhesion deficiency	partial skipping of exon-6 (149nt)	N.D.	
ITGB4 (65)	g.73732344T>A	-25	Epidermolysis bullosa with pyloric atresia	complete retention of intron-14 and intron-14/15	#1/1	-2
ITGB4 (66)	g.73748508T>A	-19	Epidermolysis bullosa with pyloric atresia	complete retention of intron-31, partial skipping of exon-32 (38nt)	#1/1	-2
KCNH2 (67)	g.150646165T>C	-28	Long QT syndrome	partial retention of intron-9 (147nt)	#1/1	0
L1CAM (68)	g.153131293T>G	-19	X-linked hydrocephalus	complete skipping of exon-19, partial retention of intron-18 (69nt)	#2/3	0
LCAT (69)	g.67976512A>G	-22	Fish-eye disease	complete retention of intron-4	#1/2	-2
LIPC (70)	g.58830518A>G	-14	Hypertriglyceridemia and cardiovascular disease	complete retention of intron-1, partial retention of intron-1 (13nt, 78nt)	N.D.	
LMX1B (71)	g.129377625_129377641del	-37	Nail patella syndrome	complete skipping of exon-2	#1/1	-2|-1|0

MLH1 (72)	g.37090369T>G	-26	Inherited cancer	complete skipping of exon-17	#2/3	-2
MLH1 (72)	g.37090371A>G	-24	Inherited cancer	complete skipping of exon-17	#2/3	0
MSH2 (73)	g.47709894A>G	-24	Inherited cancer	complete skipping of exon-16 and 3'UTR, partial retention of intron-15 (85nt, 141nt)	#1/1	0
MSH6 (72)	g.48032731T>G	-26	Inherited cancer	complete skipping of exon-5	#2/3	-2
NPC1 (74)	g.21137182T>C	-28	Niemann-Pick type C disease	complete skipping of exon-7	#1/1	0
NTRK1 (75)	g.156843392T>A	-33	Congenital insensitivity to pain with anhidrosis	partial retention of intron-7 (137nt)	#1/1	-2
RB1 (76)	g.49039315A>T	-26	Retinoblastoma	complete skipping of exon-24	#1/2	-1
SLC25A20 (77)	g.48921567A>C	-10	Carnitineacylcarnitine translocase deficiency	complete skipping of exon-3 and exon-3/4	N.D.	
TH (78)	g.31499327_31499349del	-31	Familial renal glycosuria	complete skipping of exon-8	#1/2	-2|-1|0
TSC2 (79)	g.2187017A>T	-24	Extrapyramidal movement disorder	complete skipping of exon-12, partial retention of intron-11 (36nt)	#3/3	-2
UROS (80)	g.2138031A>G	-18	Tuberous sclerosis	complete retention of intron-38, partial skipping of exon-39 (74nt)	#1/2	0
USH2A (81)	g.127477605A>C	-31	Congenital erythropoietic porphyria	partial retention of intron-9 (81nt, 246nt, 358nt, 523nt)	#1/1	-2
VMA21 (82)	g.216040529T>C	-17	Usher syndrome	partial skipping of exon-44 (39nt)	N.D.	
VMA21 (82)	g.150572076A>C	-27	Autophagic vacuolar myopathy	showed significant reduction of expression and activity	#1/1	0
VWF (83)	g.150572076A>T	-27	Autophagic vacuolar myopathy	showed significant reduction of expression and activity	#1/1	0
XPC (84)	g.6101204T>A	-20	von Willebrand disease	complete skipping of exon-38	#1/2	0
XPC (84)	g.14209889A>T	-9	Xeroderma pigmentosum	complete skipping of exon-4	N.D.	
NPC1 (74)	g.14209904T>C	-24	Xeroderma pigmentosum	complete skipping of exon-4	#1/1	0

Table S9: Genome-wide detection of BP variant candidates, and validation of their biochemical consequences from their paired WES and RNA-seq data.

Sample	Detection from WES data	Validation by paired RNA-seq data			
	# BP variants (SNVs and deletions with score ≥3, and passed quality checking)	Cell type	No/very low expression	Expressed, with mis-splicing evidence	Expressed, without mis-splicing evidence
S1	1	fibroblasts	1	0	0
S2	2	fibroblasts	1	0	1
S3	5	fibroblasts	2	2	1
S4	11	PBMC	8	2	1
S5	9	pDC	5	0	4
S6	7	neurons	4	0	3
S7	5	neurons	3	0	2
S8	3	fibroblasts/neurons	1	1	1
S9	4	EBV	1	1	2
S10	3	EBV	2	1	0
S11	5	EBV	4	0	1
S12	6	EBV	3	2	1
S13	1	EBV	1	0	0
S14	7	EBV	3	2	2
TOTAL	69		39	11	19

Table S10: Prediction of BP positions (cBP_BPHunter) in region [-3, -40] nt of 3'ss. Three machine learning methods (GBM, RF and LR) were developed by training on 198,256 adjusted eBP positions versus 1,000,000 random intronic/exonic positions, optimized by parameter tuning and threshold tuning to reach high-precision performance, and then combined to make final predictions on a majority voting basis.

Parameter optimization *(by evaluating F1 score, italics: names of parameters, *: highest F1 score)*

			learning_rate				
			0.1	0.3	0.5	0.7	1
**GBM**	*estimators*	100	0.61646	0.62917	0.63223	0.63591	0.63345
		500	0.63380	0.64049	0.64371	0.64365	0.64156
		1000	0.63954	0.64485	0.64549	0.64561	0.64552
		1500	0.64127	0.64496	0.64732	0.64714	0.64714
		2000	0.64239	0.64727	*0.64898	0.64795	0.64638
		2500	0.64374	0.64805	0.64736	0.64663	0.64593
		3000	0.64519	0.64857	0.64828	0.64790	0.64445

**RF**	*estimators*	100	0.63872
		500	0.64171
		1000	0.64261
		1500	0.64230
		2000	*0.64297
		2500	0.64268
		3000	0.64233
**LR**	*C*	0.01	0.61844
		0.1	0.61853
		1	0.61851
		10	*0.61893
		100	0.61822

Optimal parameters

	Parameter	Value	F1 Score
**GBM**	*estimators*	2000	0. 64898
	learning_rate	0.5	
	max_features	sqrt	
**RF**	*estimators*	2000	0. 64297
	bootstrap	TRUE	
	max_features	sqrt	
**LR**	*C*	10	0. 61893
	penalty	l2	
	solver	sag	

Optimal thresholds

	Threshold	Precision	Recall
**GBM**	0.95	0.95	0.04
**RF**	0.92	0.95	0.19
**LR**	0.94	0.95	0.02
**Majority Voted**		0.9952	0.075

Threshold optimization
(by precision-recall curve (PRC) on the left and receiver operating characteristic curve (ROC) on the right)

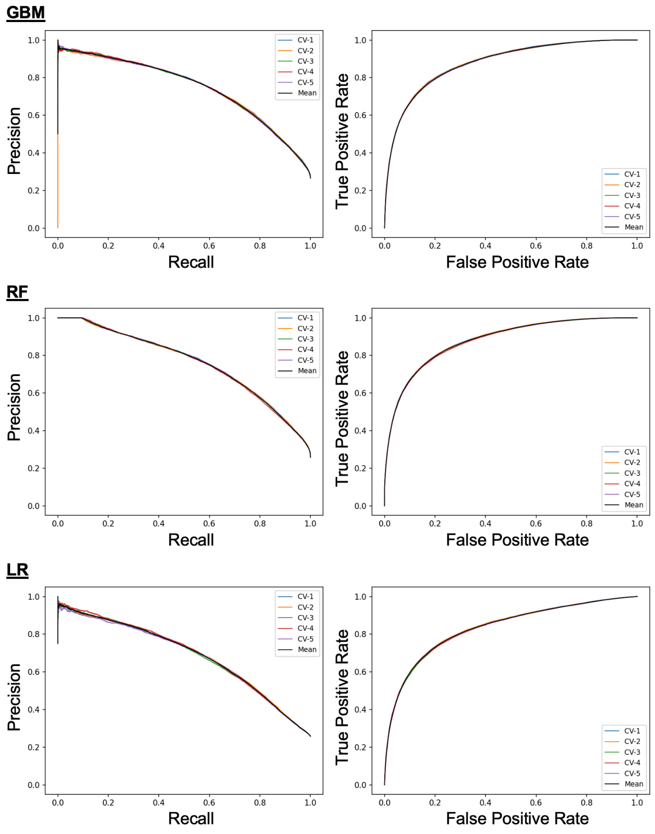

Box S1: The typical discovery narrative of the published pathogenic BP variants.

Investigators had (1) one or more families, or a cohort of patients with the same disease; (2) mostly performed targeted sequencing on the known disease-associated genes, whilst a few performed massive parallel sequencing; (3) sometimes failed to detect candidate variants in the coding regions or essential splice sites of the known disease-associated genes, and hence had extended the search to intronic variants; or sometimes studied all variants of the known disease-associated genes; (4) found one intronic variant upstream of a 3'ss, sometimes displaying an enrichment or family segregation; (5) identified the variant residing in a region matching the BP consensus sequence (YTNAY, or relaxed TNA), and consequently suspected that the variant might disrupt BP; or in some cases directly suspected the variant might disrupt BP without consensus sequence justification; and (6) performed *in vitro* expression/functional assays to reveal the mis-splicing consequences of the known disease-associated gene, therefore concluding that the intronic variant in BP sites was disease-causing.

REFERENCE

1. T. R. Mercer *et al.*, Genome-wide discovery of human splicing branchpoints. *Genome Res* **25**, 290-303 (2015).
2. A. J. Taggart *et al.*, Large-scale analysis of branchpoint usage across species and cell lines. *Genome Res* **27**, 639-649 (2017).
3. J. M. B. Pineda, R. K. Bradley, Most human introns are recognized via multiple and tissue-specific branchpoints. *Genes Dev* **32**, 577-591 (2018).
4. G. J. S. Talhouarne, J. G. Gall, Lariat intronic RNAs in the cytoplasm of vertebrate cells. *Proc Natl Acad Sci U S A* **115**, E7970-E7977 (2018).
5. M. Briese *et al.*, A systems view of spliceosomal assembly and branchpoints with iCLIP. *Nat Struct Mol Biol* **26**, 930-940 (2019).
6. D. A. Bitton *et al.*, LaSSO, a strategy for genome-wide mapping of intronic lariats and branch points using RNA-seq. *Genome Res* **24**, 1169-1179 (2014).
7. S. Y. Zhang *et al.*, Inborn Errors of RNA Lariat Metabolism in Humans with Brainstem Viral Infection. *Cell* **172**, 952-965 e918 (2018).
8. A. Dobin *et al.*, STAR: ultrafast universal RNA-seq aligner. *Bioinformatics* **29**, 15-21 (2013).
9. A. M. Bolger, M. Lohse, B. Usadel, Trimmomatic: a flexible trimmer for Illumina sequence data. *Bioinformatics* **30**, 2114-2120 (2014).
10. C. Camacho *et al.*, BLAST+: architecture and applications. *BMC Bioinformatics* **10**, 421 (2009).
11. A. Frankish *et al.*, GENCODE reference annotation for the human and mouse genomes. *Nucleic Acids Res* **47**, D766-D773 (2019).
12. Q. Zhang *et al.*, BPP: a sequence-based algorithm for branch point prediction. *Bioinformatics* **33**, 3166-3172 (2017).
13. B. Signal, B. S. Gloss, M. E. Dinger, T. R. Mercer, Machine learning annotation of human branchpoints. *Bioinformatics* **34**, 920-927 (2018).
14. J. M. Paggi, G. Bejerano, A sequence-based, deep learning model accurately predicts RNA splicing branchpoints. *RNA* **24**, 1647-1658 (2018).
15. J. Tholen, M. Razew, F. Weis, W. P. Galej, Structural basis of branch site recognition by the human spliceosome. *Science* **375**, 50-57 (2022).
16. F. Pedregosa *et al.*, Scikit-learn: Machine Learning in Python. *Journal of Machine Learning Research* **12**, 2825-2830 (2011).
17. D. C. Moyer, G. E. Larue, C. E. Hershberger, S. W. Roy, R. A. Padgett, Comprehensive database and evolutionary dynamics of U12-type introns. *Nucleic Acids Res* **48**, 7066-7078 (2020).
18. A. M. Olthof, K. C. Hyatt, R. N. Kanadia, Minor intron splicing revisited: identification of new minor intron-containing genes and tissue-dependent retention and alternative splicing of minor introns. *BMC Genomics* **20**, 686 (2019).
19. J. Morales *et al.*, A joint NCBI and EMBL-EBI transcript set for clinical genomics and research. *Nature* **604**, 310-315 (2022).
20. A. R. Quinlan, I. M. Hall, BEDTools: a flexible suite of utilities for comparing genomic features. *Bioinformatics* **26**, 841-842 (2010).
21. O. Bembom, seqLogo: Sequence logos for DNA sequence alignments. *R package* (2017).
22. J. J. Turunen, E. H. Niemela, B. Verma, M. J. Frilander, The significant other: splicing by the minor spliceosome. *Wiley Interdiscip Rev RNA* **4**, 61-76 (2013).
23. R. Lorenz *et al.*, ViennaRNA Package 2.0. *Algorithms Mol Biol* **6**, 26 (2011).
24. T. L. Bailey *et al.*, MEME SUITE: tools for motif discovery and searching. *Nucleic Acids Res* **37**, W202-208 (2009).
25. K. J. Karczewski *et al.*, The mutational constraint spectrum quantified from variation in 141,456 humans. *Nature* **581**, 434-443 (2020).
26. R. M. Kuhn, D. Haussler, W. J. Kent, The UCSC genome browser and associated tools. *Brief Bioinform* **14**, 144-161 (2013).

27. G. M. Cooper *et al.*, Distribution and intensity of constraint in mammalian genomic sequence. *Genome Res* **15**, 901-913 (2005).
28. K. S. Pollard, M. J. Hubisz, K. R. Rosenbloom, A. Siepel, Detection of nonneutral substitution rates on mammalian phylogenies. *Genome Res* **20**, 110-121 (2010).
29. P. Rentzsch, D. Witten, G. M. Cooper, J. Shendure, M. Kircher, CADD: predicting the deleteriousness of variants throughout the human genome. *Nucleic Acids Res* **47**, D886-D894 (2019).
30. P. Zhang *et al.*, PopViz: a webserver for visualizing minor allele frequencies and damage prediction scores of human genetic variations. *Bioinformatics* **34**, 4307-4309 (2018).
31. P. Zhang *et al.*, A computational approach for detecting physiological homogeneity in the midst of genetic heterogeneity. *Am J Hum Genet* **108**, 1012-1025 (2021).
32. K. Jaganathan *et al.*, Predicting Splicing from Primary Sequence with Deep Learning. *Cell* **176**, 535-548 e524 (2019).
33. J. Cheng *et al.*, MMSplice: modular modeling improves the predictions of genetic variant effects on splicing. *Genome Biol* **20**, 48 (2019).
34. P. Zhang *et al.*, SeqTailor: a user-friendly webserver for the extraction of DNA or protein sequences from next-generation sequencing data. *Nucleic Acids Res* 10.1093/nar/gkz326 (2019).
35. G. Yeo, C. B. Burge, Maximum entropy modeling of short sequence motifs with applications to RNA splicing signals. *J Comput Biol* **11**, 377-394 (2004).
36. J. L. Casanova, H. C. Su, C. H. G. Effort, A global effort to define the human genetics of protective immunity to SARS-CoV-2 infection. *Cell* 10.1016/j.cell.2020.05.016 (2020).
37. W. Ren *et al.*, Genetic landscape of hepatitis B virus-associated diffuse large B-cell lymphoma. *Blood* **131**, 2670-2681 (2018).
38. X. Ye *et al.*, Genome-wide mutational signatures revealed distinct developmental paths for human B cell lymphomas. *J Exp Med* **218** (2021).
39. K. Georgiou *et al.*, Genetic basis of PD-L1 overexpression in diffuse large B-cell lymphomas. *Blood* **127**, 3026-3034 (2016).
40. J. G. Tate *et al.*, COSMIC: the Catalogue Of Somatic Mutations In Cancer. *Nucleic Acids Res* **47**, D941-D947 (2019).
41. A. Liberzon *et al.*, The Molecular Signatures Database (MSigDB) hallmark gene set collection. *Cell Syst* **1**, 417-425 (2015).
42. P. M. Thomas *et al.*, Inactivation of the first nucleotide-binding fold of the sulfonylurea receptor, and familial persistent hyperinsulinemic hypoglycemia of infancy. *Am J Hum Genet* **59**, 510-518 (1996).
43. B. Mentrup, H. Girschick, F. Jakob, C. Hofmann, A homozygous intronic branch-point deletion in the ALPL gene causes infantile hypophosphatasia. *Bone* **94**, 75-83 (2017).
44. Z. Fadaie *et al.*, BBS1 branchpoint variant is associated with non-syndromic retinitis pigmentosa. *J Med Genet* 10.1136/jmedgenet-2020-107626 (2021).
45. S. Hashimoto *et al.*, Identification of Bruton's tyrosine kinase (Btk) gene mutations and characterization of the derived proteins in 35 X-linked agammaglobulinemia families: a nationwide study of Btk deficiency in Japan. *Blood* **88**, 561-573 (1996).
46. Z. Wang *et al.*, Axial Spondylometaphyseal Dysplasia Is Caused by C21orf2 Mutations. *PLoS One* **11**, e0150555 (2016).
47. Y. Hu *et al.*, Identification of a Novel Deep Intronic Mutation in CAPN3 Presenting a Promising Target for Therapeutic Splice Modulation. *J Neuromuscul Dis* **6**, 475-483 (2019).
48. X. Zhu, I. Chung, M. R. O'Gorman, P. R. Scholl, Coexpression of normal and mutated CD40 ligand with deletion of a putative RNA lariat branchpoint sequence in X-linked hyper-IgM syndrome. *Clin Immunol* **99**, 334-339 (2001).
49. K. M. Knapp, J. Murray, I. K. Temple, L. S. Bicknell, Successful pregnancies in an adult with Meier-Gorlin syndrome harboring biallelic CDT1 variants. *Am J Med Genet A* 10.1002/ajmg.a.62016 (2020).
50. C. Chiereghin *et al.*, Alport syndrome cold cases: Missing mutations identified by exome sequencing and functional analysis. *PLoS One* **12**, e0178630 (2017).

51. N. P. Burrows *et al.*, A point mutation in an intronic branch site results in aberrant splicing of COL5A1 and in Ehlers-Danlos syndrome type II in two British families. *Am J Hum Genet* **63**, 390-398 (1998).
52. B. Drera *et al.*, Branch point and donor splice-site COL7A1 mutations in mild recessive dystrophic epidermolysis bullosa. *Br J Dermatol* **161**, 464-467 (2009).
53. J. Isler, V. Rufenacht, C. Gemperle, G. Allegri, J. Haberle, Improvement of diagnostic yield in carbamoylphosphate synthetase 1 (CPS1) molecular genetic investigation by RNA sequencing. *JIMD Rep* **52**, 28-34 (2020).
54. M. Sinnreich, C. Therrien, G. Karpati, Lariat branch point mutation in the dysferlin gene with mild limb-girdle muscular dystrophy. *Neurology* **66**, 1114-1116 (2006).
55. R. E. Harrison *et al.*, Transforming growth factor-beta receptor mutations and pulmonary arterial hypertension in childhood. *Circulation* **111**, 435-441 (2005).
56. X. Wang, Q. Hu, N. Tang, Y. Lu, J. Deng, Deep intronic F8 c.5999-27A>G variant causes exon 19 skipping and leads to moderate hemophilia A. *Blood Coagul Fibrinolysis* **31**, 476-480 (2020).
57. R. P. Ketterling *et al.*, Reported in vivo splice-site mutations in the factor IX gene: severity of splicing defects and a hypothesis for predicting deleterious splice donor mutations. *Hum Mutat* **13**, 221-231 (1999).
58. N. Agrebi *et al.*, Rare splicing defects of FAS underly severe recessive autoimmune lymphoproliferative syndrome. *Clin Immunol* **183**, 17-23 (2017).
59. C. Maslen, D. Babcock, M. Raghunath, B. Steinmann, A rare branch-point mutation is associated with missplicing of fibrillin-2 in a large family with congenital contractural arachnodactyly. *Am J Hum Genet* **60**, 1389-1398 (1997).
60. E. A. Putnam, E. S. Park, C. M. Aalfs, R. C. Hennekam, D. M. Milewicz, Parental somatic and germ-line mosaicism for a FBN2 mutation and analysis of FBN2 transcript levels in dermal fibroblasts. *Am J Hum Genet* **60**, 818-827 (1997).
61. E. Aten *et al.*, Exome sequencing identifies a branch point variant in Aarskog-Scott syndrome. *Hum Mutat* **34**, 430-434 (2013).
62. M. Fujimaru *et al.*, Two mutations remote from an exon/intron junction in the beta-hexosaminidase beta-subunit gene affect 3'-splice site selection and cause Sandhoff disease. *Hum Genet* **103**, 462-469 (1998).
63. S. E. Jorgensen *et al.*, Ectodermal dysplasia with immunodeficiency caused by a branch-point mutation in IKBKG/NEMO. *J Allergy Clin Immunol* **138**, 1706-1709 e1704 (2016).
64. D. Roos *et al.*, Genetic analysis of patients with leukocyte adhesion deficiency: genomic sequencing reveals otherwise undetectable mutations. *Exp Hematol* **30**, 252-261 (2002).
65. T. Masunaga *et al.*, Splicing abnormality of integrin beta4 gene (ITGB4) due to nucleotide substitutions far from splice site underlies pyloric atresia-junctional epidermolysis bullosa syndrome. *J Dermatol Sci* **78**, 61-66 (2015).
66. S. Chavanas *et al.*, Splicing modulation of integrin beta4 pre-mRNA carrying a branch point mutation underlies epidermolysis bullosa with pyloric atresia undergoing spontaneous amelioration with ageing. *Hum Mol Genet* **8**, 2097-2105 (1999).
67. L. Crotti *et al.*, A KCNH2 branch point mutation causing aberrant splicing contributes to an explanation of genotype-negative long QT syndrome. *Heart Rhythm* **6**, 212-218 (2009).
68. A. Rosenthal, M. Jouet, S. Kenwrick, Aberrant splicing of neural cell adhesion molecule L1 mRNA in a family with X-linked hydrocephalus. *Nat Genet* **2**, 107-112 (1992).
69. J. A. Kuivenhoven *et al.*, An intronic mutation in a lariat branchpoint sequence is a direct cause of an inherited human disorder (fish-eye disease). *J Clin Invest* **98**, 358-364 (1996).
70. K. Brand, K. A. Dugi, J. D. Brunzell, D. N. Nevin, S. Santamarina-Fojo, A novel A-->G mutation in intron I of the hepatic lipase gene leads to alternative splicing resulting in enzyme deficiency. *J Lipid Res* **37**, 1213-1223 (1996).
71. J. D. Hamlington, M. V. Clough, J. A. Dunston, I. McIntosh, Deletion of a branch-point consensus sequence in the LMX1B gene causes exon skipping in a family with nail patella syndrome. *Eur J Hum Genet* **8**, 311-314 (2000).
72. D. M. Canson *et al.*, The splicing effect of variants at branchpoint elements in cancer genes. *Genet Med* 10.1016/j.gim.2021.09.020 (2021).

73. S. Casadei *et al.*, Characterization of splice-altering mutations in inherited predisposition to cancer. *Proc Natl Acad Sci U S A* 10.1073/pnas.1915608116 (2019).
74. E. Di Leo *et al.*, A point mutation in the lariat branch point of intron 6 of NPC1 as the cause of abnormal pre-mRNA splicing in Niemann-Pick type C disease. *Hum Mutat* **24**, 440 (2004).
75. Y. Miura *et al.*, Mutation and polymorphism analysis of the TRKA (NTRK1) gene encoding a high-affinity receptor for nerve growth factor in congenital insensitivity to pain with anhidrosis (CIPA) families. *Hum Genet* **106**, 116-124 (2000).
76. K. Zhang, I. Nowak, D. Rushlow, B. L. Gallie, D. R. Lohmann, Patterns of missplicing caused by RB1 gene mutations in patients with retinoblastoma and association with phenotypic expression. *Hum Mutat* **29**, 475-484 (2008).
77. B. Y. Hsu *et al.*, Aberrant mRNA splicing associated with coding region mutations in children with carnitine-acylcarnitine translocase deficiency. *Mol Genet Metab* **74**, 248-255 (2001).
78. R. J. Janssen *et al.*, A branch site mutation leading to aberrant splicing of the human tyrosine hydroxylase gene in a child with a severe extrapyramidal movement disorder. *Ann Hum Genet* **64**, 375-382 (2000).
79. K. Mayer, W. Ballhausen, W. Leistner, H. Rott, Three novel types of splicing aberrations in the tuberous sclerosis TSC2 gene caused by mutations apart from splice consensus sequences. *Biochim Biophys Acta* **1502**, 495-507 (2000).
80. D. F. Bishop *et al.*, Congenital erythropoietic porphyria: a novel uroporphyrinogen III synthase branchpoint mutation reveals underlying wild-type alternatively spliced transcripts. *Blood* **115**, 1062-1069 (2010).
81. S. Le Guedard-Mereuze *et al.*, Ex vivo splicing assays of mutations at noncanonical positions of splice sites in USHER genes. *Hum Mutat* **31**, 347-355 (2010).
82. N. Ramachandran *et al.*, VMA21 deficiency prevents vacuolar ATPase assembly and causes autophagic vacuolar myopathy. *Acta Neuropathol* **125**, 439-457 (2013).
83. L. Hawke *et al.*, Characterization of aberrant splicing of von Willebrand factor in von Willebrand disease: an underrecognized mechanism. *Blood* **128**, 584-593 (2016).
84. S. G. Khan *et al.*, Two essential splice lariat branchpoint sequences in one intron in a xeroderma pigmentosum DNA repair gene: mutations result in reduced XPC mRNA levels that correlate with cancer risk. *Hum Mol Genet* **13**, 343-352 (2004).
