# Supplementary material for: Genome-wide detection of human variants that disrupt intronic branchpoints
Source: Proc Natl Acad Sci U S A. 2022 Oct 28;119(44):e2211194119. doi: 10.1073/pnas.2211194119 (PMC9636908; doi:10.1073/pnas.2211194119)
Supplement: Supplementary File [file pnas.2211194119.sd06.pdf]

BPHunter: Negative BP Reporting

** We genuinely require all submitters to take responsibility for the submitted data.*
** Please name your report as: "BPHunter_Neg_Rep_MM_DD_YYYY_LASTNAME.docx", where "MM_DD_YYYY" is the date.*
** The submitters should be aware of that the collected results will be analyzed for future research, if we receive enough.*

Submission Date (MM/DD/YYYY)	

Submitter's Information

Prefix:	
First Name:	
Last Name:	
Institutional Email:	
Academic Position:	
Laboratory:	
Principle Investigator:	
Institution:	
City, State:	
Country:	

Variant Information
**add rows if more variants to be submitted*

Reference Genome (GRCh37/GRCh38):	
Number of Variants:	

Gene	CHROM	POS	REF	ALT	Type (snv, del, ins)	Zygosity (hom, het, hemi)	BP Name (given by BPHunter)	Hit position (-2, -1, 0, all)

Experimental Information
** attach the supporting evidence (figures and descriptions) starting form the next page*

Cell Type:	
Assay Type:	

<u>Supporting Evidence</u>
